# Cognitive ability in childhood predicts adolescent structural and functional brain development: A longitudinal study

**DOI:** 10.1016/j.dcn.2026.101768

**Published:** 2026-06-18

**Authors:** Phoebe Thomson, Philip Shaw, Divyangana Rakesh

**Affiliations:** a Department of Neuroimaging, Institute of Psychiatry, Psychology & Neuroscience, King’s College London, London, UK; b King's Maudsley Partnership for Children and Young People and Department of Child and Adolescent Psychiatry, Institute of Psychiatry, Psychology and Neuroscience, King's College London, London, UK

**Keywords:** Intelligence and cognitive function, Brain structure, White matter, Functional connectivity, Adolescence, Longitudinal

## Abstract

General cognitive ability in childhood has been linked to differences in brain structure and connectivity in numerous studies. However, longitudinal examinations of links between early cognitive ability and multimodal brain development are limited. Leveraging longitudinal data from 10,495 participants of the Adolescent Brain Cognitive Development study, we examined whether childhood cognitive ability was associated with differences in longitudinal development of gray and white matter structure and resting state functional connectivity (rsFC). Linear mixed-effects models tested associations between baseline cognition—matrix reasoning, fluid cognition, crystallized cognition—and trajectories of whole-brain and regional brain measures. Brain measures included cortical thickness, surface area, and volume, fractional anisotropy, mean diffusivity, and rsFC across four time points. Across modalities, higher baseline cognitive scores were associated with slower increases in measures that normatively increased over adolescence and faster decreases for those that were found to decline. Specifically, higher childhood fluid cognition was associated with faster contraction of gray matter area and volume, and higher matrix reasoning was related to slower increases in white matter fractional anisotropy. Meanwhile, lower values in all cognitive measures—early matrix reasoning, fluid cognition, and crystallized cognition—were associated with greater increases in between-network rsFC that were absent in those with higher baseline cognitive scores. Regionally, differences in gray matter structural trajectories by cognitive ability were most prominent in the frontal lobe, while effects for white matter and rsFC trajectories varied across distributed brain systems. Findings provide insight into theories of cognition and highlight possible neurobiological pathways linking early cognition to long-term outcomes.

## Highlights

• Higher childhood cognition predicted faster decreases in area and volume in adolescence.
• Cognition-related decreases in cortical structure were most prominent in frontal regions.
• Higher childhood cognition predicted slower white matter and functional maturation.
• White matter and functional connectivity effects were present across multiple brain systems.

## 1 Introduction

Early general cognitive ability in childhood is associated with several long-term outcomes including educational attainment (Deary et al., 2007), occupational status (Fergusson et al., 2005), risk for depression and substance abuse (Fries et al., 2025), and physical health (Calvin et al., 2017). Childhood cognitive ability is also related to brain structure and connectivity (Deary et al., 2022). Adolescence is a sensitive period of brain development, characterized by substantial change in neural gray and white matter structure (Lebel et al., 2019, Tamnes et al., 2017) and functional reorganization (Edde et al., 2021). The emergence of large-scale longitudinal neuroimaging datasets provides an opportunity to study how individual variability in childhood cognition may relate to changes in brain structure and function over time. This is particularly important given the paucity of longitudinal research studying the association of early cognition with brain structural and functional connectivity development. Such work could enrich theories of cognition and point to possible neurobiological bases for risk of poorer long-term outcomes across multiple functional and health domains.

Gray matter development follows a broadly consistent pattern of early increases and later decreases, but the timing of these peaks varies across studies (Norbom et al., 2021). Surface area and volume are theorized to peak in late childhood to early adolescence, while thickness peaks earlier (Kelly et al., 2024, Norbom et al., 2021). Some longitudinal work has linked these structural changes with childhood cognitive ability (Schnack et al., 2015, Shaw et al., 2006, Sowell et al., 2004). For example, a few studies show that children with higher intelligence—based on Weschler intelligence scales—have a more protracted trajectory of cortical thickening followed by accelerated rates of thinning during adolescence, particularly evident in the prefrontal and parietal cortices (Schnack et al., 2015, Shaw et al., 2006) as well as accelerated surface contraction (Schnack et al., 2015), though others do not report this association (Estrada et al., 2019). These findings align with the *Parieto-Frontal Integration Theory* (Jung and Haier, 2007), which states that general cognitive ability specifically relates to frontal and parietal regions of the brain and builds on the *neural efficiency hypothesis* (Haier et al., 1988, Neubauer and Fink, 2009) which proposes that higher general cognitive ability is associated with more efficient brain activation during cognitive tasks. A more protracted trajectory of cortical thickening followed by accelerated rates of thinning during adolescence may reflect extended windows of plasticity which allow for more efficient brain activation and higher cognition (Neubauer and Fink, 2009, Scholl et al., 2021). Despite established cross-sectional links between gray matter volume and cognition (Luders et al., 2009), less is known about how childhood cognitive ability may relate with longitudinal changes in volume over time. Thus, while there is some preliminary support suggesting early cognition predicts brain development over time, findings remain limited and are drawn from modestly sized longitudinal samples (n = 307–504) (Estrada et al., 2019, Schnack et al., 2015, Shaw et al., 2006). The emergence of large-scale developmental neuroimaging datasets, including those with longitudinal repeated imaging assessments, provides an opportunity to study how individual variability in childhood cognition may relate to changes in brain structure and function over time.

Beyond a lack of studies examining longitudinal changes in gray matter structure, examinations of relationships between structural and functional connectivity — which undergo substantial changes during development — and early cognition are limited. The development of white matter connectivity over adolescence is characterized by gradual increases in fractional anisotropy (FA) and decreases in mean diffusivity (MD), which capture underlying changes in myelination and axon density (Lebel et al., 2019). Cross-sectional evidence indicates that for those with higher cognitive ability, FA and diffusivity may plateau earlier in development (Tamnes et al., 2010), although this is speculative without longitudinal data. These findings, which implicated frontoparietal regions, as well as tracts including the anterior thalamic radiation, corticospinal tract, superior longitudinal fasciculus and corpus callosum, suggest that while frontoparietal regions and tracts are relevant, associations between cognition and the brain may be more widespread. The emerging view that general cognitive ability depends on distributed, dynamically interacting systems—captured by the *Network Neuroscience Theory* (Barbey, 2018)—emphasizes the importance of interactions among several brain systems, including frontoparietal, default mode, and somatosensory networks. However, longitudinal research is required to understand how general cognition relates to within-person changes in white matter across the brain over adolescence.

Further, within-network resting-state functional connectivity (rsFC) shows modest increases throughout childhood and adolescence (Edde et al., 2021). This holds true for most networks, particularly the default mode and visual networks (Rakesh et al., 2021, Rakesh et al., 2025, Van Duijvenvoorde et al., 2019). This is coupled with gradual increases in global rsFC between functional networks throughout development, which helps to ultimately achieve a balance between specialization and integration in the brain (Edde et al., 2021). However, these developmental trajectories of between-network rsFC vary by functional networks. For example, rsFC between higher-order cognitive networks—such as the frontoparietal, cingulo-opercular and attention networks—may increase during adolescence, while their connectivity with the default mode network decreases over time (Rakesh et al., 2021, Sanders et al., 2023). Cross-sectionally, higher general cognitive ability has been found to be associated with higher rsFC within the frontoparietal network and reduced rsFC between frontoparietal and default mode networks (DeSerisy et al., 2021, Langeslag et al., 2013; C. Li and Tian, 2014). However, there is a preponderance of cross-sectional studies, and a focus on the frontoparietal and default mode networks, despite evidence of more widespread network involvement in cognitive ability in adults (Wilcox and Barbey, 2023). More longitudinal studies that examine how early cognitive ability relates to changes in brain structural and functional connectivity development are therefore needed.

Finally, cognitive ability is a broad construct and three strands of evidence underscore the importance of treating domains of cognitive ability as related but separable constructs in longitudinal research on neurocognitive development. First, theories of cognitive ability such as the Cattell-Horn-Carroll theory separate abilities into multiple strata (McGrew, 2009). This includes a high-level general cognitive ability stratum, underpinned by broad domains including fluid reasoning, processing speed, comprehension-knowledge, working memory, and visual-spatial processing (McGrew, 2009). In practice, the tools used in cognitive research do not always neatly conform to these categories. The Wechsler Scales (Wechsler, 2014) measurement of *matrix reasoning* relies on visual-spatial abstract reasoning and problem solving, and overlaps most strongly with the fluid and visual-spatial processing categories of the Cattell-Horn-Carroll theory (Jewsbury et al., 2017, Keith and Reynolds, 2010). Meanwhile, other batteries such as the NIH Toolbox Cognition Battery aim to capture broader composites of multiple abilities (Gershon et al., 2013). Within the NIH Toolbox framework, crystallized cognition refers to accumulated knowledge and skills including reading and vocabulary, while the fluid cognition composite encompasses multiple aspects of information processing, inhibition, attention, memory, and processing speed (Jewsbury et al., 2016, Jewsbury et al., 2017). Second, statistical evidence finds these three measures considered here — matrix reasoning, NIH Toolbox crystallized cognition, NIH Toolbox fluid cognition — are only moderately correlated (range *r* = .24–.47) (Luciana et al., 2018). Finally, recent work shows shared and distinct neural correlates for fluid, crystallized, and matrix reasoning measures of cognition cross-sectionally (Hasanzadeh et al., 2025, Qiu et al., 2025). These three measures also differ in their sensitivity to genetic and environmental factors (Tucker-Drob and Briley, 2014), and differentially predict a range of adult outcomes (Schneider and Niklas, 2017, Wrulich et al., 2014) supporting their use as related but separable constructs in developmental research.

To address these literature gaps, we leveraged multimodal longitudinal data from the Adolescent Brain Cognitive Development (ABCD) study to provide an account of how cognitive ability in childhood may be associated with longitudinal brain development. This exploratory study advances knowledge in three ways. First, we explore the possibility of differential effects of different cognitive measures — fluid cognition, crystallized cognition, matrix reasoning — on neurodevelopment. Secondly, we provide a comprehensive mapping across multiple modalities — gray matter structure and white matter and functional connectivity at the whole-brain and regional/system level — and can thus identify whether cognition impacts brain development uniformly across different neural features. Finally, by leveraging the large sample size of ABCD, we provide more precise estimates of associations between cognition and brain development than have been reported to date.

## 2 Methods

### 2.1 Participants

Participants were from the ongoing ABCD study (https://abcdstudy.org/; release 6.0), a large-scale longitudinal study of development from late childhood to early adulthood in the United States, including neuroimaging, neurocognitive tests and survey data from 11,868 individuals. Written informed consent was obtained from all parents/caregivers; all children provided assent. Participants' rights were protected by institutional review boards of participating universities. Data was accessed from the NIH Brain Development Cohorts (NBDC) Data Sharing Platform. The present study used data from the baseline, 2-year, 4-year, and 6-year follow-up time points. Participants were included in analyses if they had complete information available (i.e., no missing data) at baseline for at least one cognitive measure (described below), at least one time point with usable neuroimaging data, and data on covariates (see supplement for details and Figure S1 flow diagram). This yielded an analytic sample of 10,495 participants for structural MRI (sMRI), 10,245 participants for diffusion MRI (dMRI), and 9992 participants for resting-state functional MRI (rsfMRI).

### 2.2 Measures

**General cognitive ability.** Cognitive ability was assessed using the NIH Toolbox and Wechsler Intelligence Scale for Children V (WISC) V (Wechsler, 2014). We used the NIH Toolbox Fluid composite age-corrected standard scores for fluid cognition, NIH Toolbox Crystallized composite age-corrected standard scores to assess crystallized cognition (Luciana et al., 2018), and total scaled score on the WISC-V Matrix Reasoning Test to capture abstract reasoning. We note that we use the terms *fluid cognition* and *crystallized cognition* throughout as defined by the abilities captured in the NIH Toolbox, which may differ from definitions in other batteries or taxonomies (Jewsbury et al., 2016, McGrew, 2009). WISC matrix reasoning scores are only moderately correlated with fluid and crystallized composite scores from the NIH Toolbox (*r* = .24–.29) (Luciana et al., 2018) and load onto several different aspects of cognition (Thompson et al., 2019), and therefore warrant investigation.

**Income to needs ratio.** Income-to-needs ratio (INR) at baseline was calculated by dividing the total household income by the 2017 Federal Poverty Guidelines for the respective household size (Department of Health and Human Services, 2017). Total household income at baseline was calculated as the median of the income band. INR values less than and greater than 1 indicate being below and above the poverty threshold, respectively. INR was used as a covariate in the current study given prior evidence that family income is associated with the pace of brain structural and functional development (Dufford and Kim, 2017, Rakesh et al., 2025, Tsomokos et al., 2026) and cognition at this age (Lawson et al., 2018, Rakesh et al., 2025).

### 2.3 Neuroimaging data

MRI acquisition for the ABCD study was conducted across sites using Siemens, GE, and Philips MRI scanners (Casey et al., 2018). T1-weighted images were acquired using a magnetization-prepared rapid acquisition gradient echo sequence with parameters: repetition time (TR) = 6.31–2500 ms, echo time (TE) = 2–2.9 ms, inversion time (TI) = 1060 ms, flip angle = 8°, field of view (FOV) = 256 × 256 mm, resolution = 1.0 mm isotropic. Diffusion-weighted images were acquired using a high angular resolution diffusion imaging across 96 directions at four b-values (b = 500, 6 directions; b = 1000, 15 directions; b = 2000, 15 directions; b = 3000, 60 directions) with parameters: TR = 4100–5300 ms, TE = 81.9–89 ms, FOV = 240 × 240 mm, slices = 81, resolution = 1.7 mm isotropic. Resting state functional images were collected with a gradient echo echo-planar imaging sequence and acquisition parameters: TR = 800 ms, TE = 30 ms, flip angle = 52°, FOV = 216 × 216 mm, slices = 60, resolution = 2.4 mm isotropic. Full details of MRI preprocessing procedures for the ABCD study have been documented previously (Casey et al., 2018, Hagler et al., 2019), and are described briefly in the Supplement. For each modality, imaging data at a given time point was included if the participant’s scan met quality control criteria recommended by the ABCD Data Analysis, Informatics & Resource Center (DAIRC; see supplement).

For each imaging modality, we extracted brain features both globally (i.e., whole-brain) and locally (i.e., region-wise for sMRI, network-wise for rsfMRI, and tract-wise for dMRI). Primary outcome measures of interest were global (i.e., whole-brain) average cortical thickness, total cortical surface area, total cortical volume, fractional anisotropy (FA), mean diffusivity (MD), between-network resting-state functional connectivity (rsFC), and within-network rsFC. For increased specificity, local measures were also examined at the level of regions (thickness, area, volume), tracts (FA, MD), and networks (rsFC). From sMRI, whole-brain average cortical thickness, total area, and total volume were provided by ABCD release 6.0 (ABCD Consortium, 2025). Given lack of hypotheses regarding lateralization, for each of the 34 brain regions of the Desikan-Killiany atlas (Desikan et al., 2006) we averaged estimates of area, volume, and thickness across hemispheres. For dMRI, we used values of whole-brain weighted average FA and MD, as well as the weighted average FA and MD from each of the 15 major white matter tracts from AtlasTrack (Hagler et al., 2009). For rsfMRI, we used the released pairwise between-network rsFC between each of the 66 unique network pairs from the Gordon parcellation (Gordon et al., 2016), and average within-network rsFC for each of the 12 Gordon networks. When then estimated whole-brain average between-network rsFC as the mean across all 66 pairwise average between-network rsFC values, and whole-brain average within-network rsFC as the mean across all 12 network-wise average within-network rsFC values. See Supplement for further details and complete list of regions, tracts, and networks examined.

**Quality control metrics.** Mean head motion for dMRI and rsfMRI scans were indexed by average framewise displacement (FD) (Power et al., 2014). Structural MRI cortical surface reconstruction quality was indexed by the number of topological defects calculated from the Euler number in FreeSurfer (Backhausen et al., 2022).

### 2.4 Statistical methods

**Longitudinal models of whole-brain measures.** Linear mixed-effects models (LMMs) were conducted in R (version 4.2.1) using the *lme4* package (Bates et al., 2015) to examine the association between baseline cognition and brain development, with p-values obtained using *lmerTest* (Kuznetsova et al., 2017). We first examined associations between cognitive ability and change in global brain measures. Brain measures of interest included 1) average cortical thickness, total surface area, and total volume, 2) average FA and MD, and 3) average between- and within-network rsFC.

To examine how baseline cognitive ability (n = 3 variables; modeled separately) relates with change in brain structure and connectivity over time, we modeled time varying brain variables as the outcome and an interaction term between cognitive scores and age as the main predictor. A separate model was estimated for each outcome variable. This allowed us to examine whether longitudinal trajectories of brain measures varied by baseline matrix reasoning, fluid cognition, or crystallized cognition. Fixed effect covariates included sex, baseline INR, scanner model, and mean FD (available for rsfMRI and dMRI models only). To ensure robustness to surface reconstruction quality, we covaried for the number of topological defects in sMRI models in an additional analysis (see Supplement). Given prior research indicating differences in the pace of brain development by sex (Kaczkurkin et al., 2019, Scheinost et al., 2015) and INR (Farah, 2017, Rakesh et al., 2023), and their role in general cognitive ability at this age (Lawson et al., 2018, Rakesh et al., 2025), we accounted for change associated with sex and baseline INR. Random effects included random intercepts for participant ID, family ID, and study site, and random slopes for participant ID. Random slopes for participant ID were included along with random intercepts as they improved model fit, with the full model equation being: *Brain measure ∼ Age × Cognitive ability + Age × Sex + Age × INR + MRI model + Mean FD + (Age | ID) + (1 | Site) + (1 | Family ID).* Continuous variables were standardized across all data prior to modeling, allowing comparison of effect sizes between modalities. As this was a hypothesis-generating study, no correction for multiple comparisons was applied at the global level; the reported associations are intended to generate hypotheses for future confirmatory research rather than to establish definitive inference. Identified interaction effects were probed using simples slopes using the *interactions* package (Long, 2019) (see Supplement for details). Additionally, to ascertain at what ages associations between global brain measures and baseline cognitive ability were present, we followed up significant longitudinal effects for global measures with exploratory cross-sectional analyses (see Supplement for details and model equation). Finally, in further exploratory analyses, we examined sex differences by including a 3-way interaction term in LMMs (i.e., sex × cognitive ability × age).

**Longitudinal models by region/network/tract.** For significant associations found in the above analyses, we conducted follow up specificity analyses. These models included regional measures of thickness, surface area and volume, FA and MD of AtlasTrack tracts, or connectivity between and within Gordon functional networks as time varying dependent variables (in separate models for each region, tract, or network). Analyses were conducted using the same longitudinal analytic framework described above. We corrected for multiple comparisons using false discovery rate (FDR) correction (*p* < .05) within each modality and cognitive measure. The number of comparisons varied based on the modality: n = 102 possible comparisons for gray matter structural development (34 regions for each of surface area, volume and thickness); n = 30 possible comparisons for white matter (15 tracts for each of FA and MD); 78 possible comparisons for rsFC (n = 66 between-network pairs, and n = 12 within-network connectivity variables). Of note, regional/individual connectivity models were only run if a significant association between a cognitive measure and brain metric development was observed in the global model (e.g., if associations for global cortical thickness and surface area were significant, regional brain structure results would be corrected for 68 comparisons; see Results). To examine age by general cognitive ability interactions for each region over and above whole-brain effects, supplementary analyses repeated regional surface area and volume models with total surface area or volume as a covariate, respectively.

**Sensitivity analyses.** As the 6-year follow up imaging data was incomplete at the time of analyses and participants had on average 2.5 imaging timepoints per person, models considered linear development in whole-brain average brain measures for primary analyses. For completeness, all models were rerun allowing for non-linear (i.e., quadratic) age effects and age interaction terms (see Supplement for model equations and the results). Additional sensitivity analyses repeated all models without the smallest study site that did not participate longitudinally to confirm robustness of findings (see Supplement). Sensitivity analyses were also run on sMRI models additionally including the number of topological defects calculated from Euler number as a covariate (see Supplement; Tables S18–21). Finally, given that socioeconomic status (SES) is a multidimensional construct (Duncan and Magnuson, 2012) and associations with brain structure and function partially differ based on the SES indicator (Rakesh et al., 2022, Rakesh et al., 2021), we reran all models using average parent educational attainment and neighborhood disadvantage at baseline as the SES covariate (in separate models) instead of INR (see Supplement; Tables S22–27).

## 3 Results

### 3.1 Demographic information

Demographic information and descriptive statistics for each data collection time point used for analyses are available in Table 1. Participants were 8.3–11.3 years old at baseline, and 10.2–13.9, 12.5–16.6 and 14.7–17.7 years old at the subsequent three imaging time points.

**Table 1:** Demographic information.

	**Baseline**	**2-year** **follow up**	**4-year** **follow up**	**6-year** **follow up**
Total, *n*	10133*	7165*	5716*	3769*
Age in years, *M (SD)*	9.96 (0.62)	12.00 (0.65)	14.17 (0.72)	16.10 (0.65)
Matrix reasoning, *M (SD)*	10.00 (2.95)	–	–	–
Fluid cognition, *M (SD)*	96.34 (17.26)	–	–	–
Crystallized cognition, *M (SD)*	106.49 (18.23)	–	–	–
Income-to-needs, *M (SD)*	3.69 (2.49)	–	–	–
Sex				
Male, *n* (%)	5422 (52%)	3966 (54%)	3096 (53%)	1987 (52%)
Female, *n* (%)	5034 (48%)	3431 (46%)	2729 (47%)	1814 (48%)
MRI data included				
Structural	10133	7165	5716	3769
Diffusion	9180	6800	5508	3605
rsfMRI	8469	6344	5381	3549
MRI data quality				
Structural QC, *M (SD)*	22.14 (12.73)	20.35 (10.77)	18.76 (10.86)	16.49 (8.33)
Diffusion mean FD, *M (SD)*	1.32 (0.40)	1.19 (0.38)	1.15 (0.43)	1.12 (0.31)
rsfMRI mean FD, *M (SD)*	0.23 (0.21)	0.17 (0.17)	0.12 (0.11)	0.10 (0.07)

Note: Sample size and demographic information (mean [*M*] and standard deviation [*SD*], or count [*n*] and percent [%]) are reported at each time point for the largest possible sample. This included participants who had at least one measure of general cognitive ability at baseline (i.e., Wechsler Intelligence Scale for Children - Matrix Reasoning scaled score, NIH Toolbox age-corrected crystallized score or NIH Toolbox age-corrected fluid score), in addition to sex, study site and income-to-needs at baseline, and neuroimaging data (regardless of modality) at any time point. MRI data quality was indexed by framewise displacement (FD) for diffusion and resting-state functional MRI (rsfMRI), and the number of topological defects from the Euler number for structural MRI. * The total data available were from 11868 participants at baseline, 10973 at 2-year follow-up, 9739 at 4-year follow-up and 5056 at 6-year follow up; after exclusions for missing information or imaging quality control, the analytic sample for the current study comprised 10133 (85%), 7165 (65%), 5716 (59%) and 3769 (75%) participants at each timepoint, respectively. Quality control = QC.

### 3.2 General cognitive ability in childhood and brain gray matter structural development

**Fig. 1:**
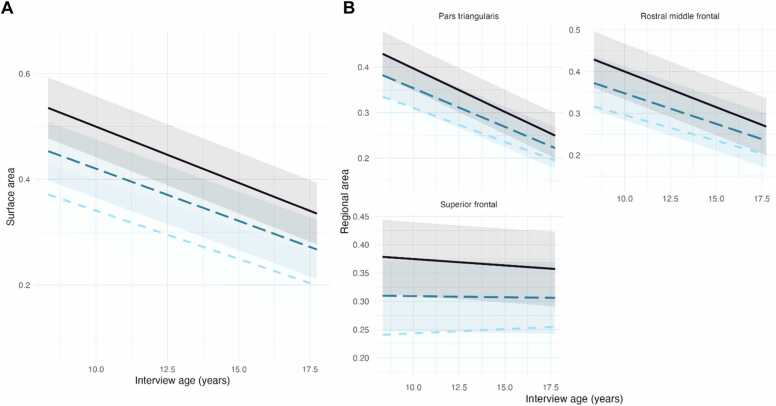
Association between the development of surface area and childhood general cognitive ability indexed by fluid cognition (NIH Toolbox) at the **A)** whole-brain and **B)** regional level. Relationships in (B) are displayed for regions for which longitudinal surface area trajectories significantly differed by fluid cognition. Best fit lines were estimated and displayed for different baseline fluid cognition values (light teal=mean − 1 SD, dark teal=mean, black=mean + 1 SD).

**Fig. 2:**
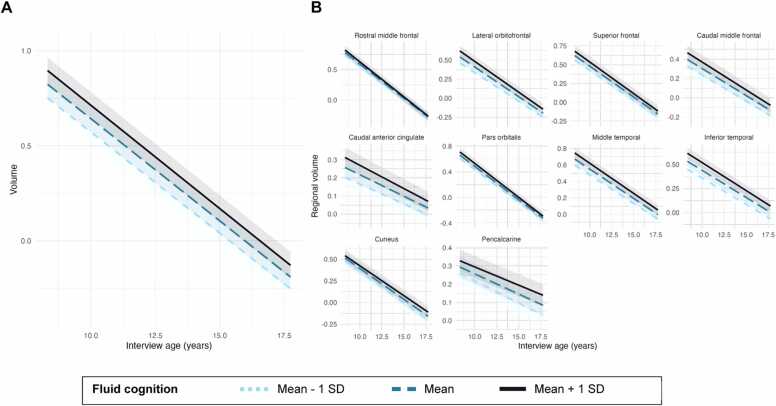
Association between the development of gray matter volume and childhood general cognitive ability indexed by fluid cognition (NIH Toolbox) at the **A)** whole-brain and **B)** regional level. Relationships in (B) are displayed for regions for which longitudinal surface area trajectories significantly differed by fluid cognition. Best fit lines were estimated and displayed for different baseline fluid cognition values (light teal=mean − 1 SD, dark teal=mean, black=mean + 1 SD).

**Fig. 3:**
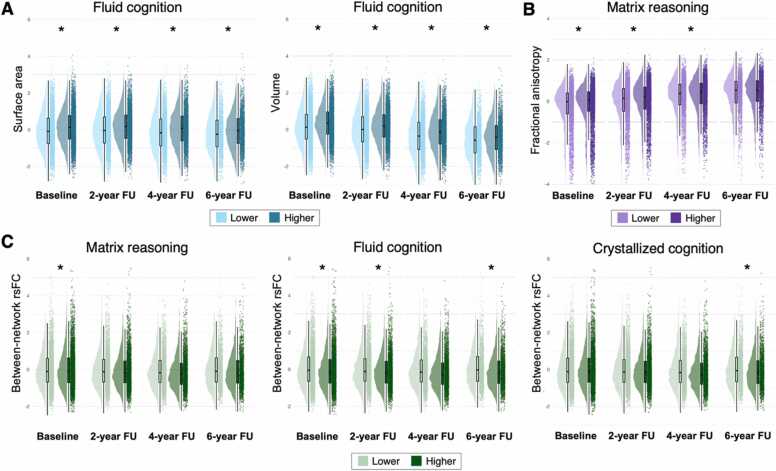
At each timepoint, cross-sectional differences in global brain measures are presented as raincloud plots for youth with lower (light color) or higher (dark color) baseline cognition. Cross-sectional differences examined included: **A)** surface area and volume by fluid cognition (NIH Toolbox); **B)** fractional anisotropy by matrix reasoning (Wechsler Intelligence Scale for Children); **C)** between-network resting-state functional connectivity (rsFC) by matrix reasoning, fluid cognition, and crystallized cognition (NIH Toolbox), respectively. Groups based on a median split by cognitive measure. * Indicates uncorrected *p* < .05. Brain measures were standardized prior to analysis; for interpretation we provide the mean (SD) of each measure: surface area = 188733 (18220), volume = 582693 (59000), fractional anisotropy = 0.499 (0.029), and between-network rsFC = −0.0001 (0.0098). FU = follow-up timepoint.

**Whole-brain gray matter structure.** Fluid cognition at baseline was significantly associated with longitudinal development of whole-brain cortical surface area (*p* = .001; see Table 2, Fig. 1a) and volume (*p* = .034; see Table 2, Fig. 2a). More specifically, those with higher baseline fluid cognition showed a faster decrease in surface area over time (β=−0.048, *p* < .001) than those with mean (β=−0.044, *p* < .001) or lower baseline fluid cognition (β=−0.041, *p* < .001; see Table S2 for simple slopes results). Similarly, those with higher baseline fluid cognition showed a faster decrease in volume (β=−0.242, *p* < .001) compared to those with mean (β=−0.240, *p* < .001) or lower baseline fluid cognition (β=−0.237, *p* < .001). Cross-sectionally, fluid cognition was positively associated with surface area and volume at all timepoints (Fig. 3, Table S3).

**Table 2:** Baseline general cognitive ability and whole brain measures of brain development.

**Modality**	**Cognitive measure**	**β**	**SE**	**df**	**t**	**p**
***Gray matter structure***						
Cortical thickness	Matrix reasoning	0.0024	0.0027	6598	0.91	.364
	Fluid cognition	0.0040	0.0027	6360	1.52	.128
	Crystallized cognition	0.0046	0.0027	6433	1.71	.088
Surface area	Matrix reasoning	0.0009	0.0010	6553	0.89	.372
	Fluid cognition	−0.0033	0.0010	6363	−3.29	**.001**
	Crystallized cognition	−0.0002	0.0010	6466	−0.18	.854
Volume	Matrix reasoning	−0.0003	0.0012	6815	−0.24	.808
	Fluid cognition	−0.0026	0.0012	6555	−2.12	**.034**
	Crystallized cognition	−0.0021	0.0013	6659	−1.66	.098
***White matter***						
Fractional anisotropy	Matrix reasoning	−0.0065	0.0028	7565	−2.33	**.020**
	Fluid cognition	0.0002	0.0028	7285	0.07	.946
	Crystallized cognition	0.0018	0.0029	7372	0.65	.518
Mean diffusivity	Matrix reasoning	0.0002	0.0033	7548	0.07	.945
	Fluid cognition	0.0054	0.0033	7270	1.61	.109
	Crystallized cognition	0.0040	0.0034	7343	1.19	.236
***Functional connectivity***						
Between-network FC	Matrix reasoning	−0.0283	0.0060	6934	−4.75	**< .001**
	Fluid cognition	−0.0161	0.0059	6764	−2.72	**.007**
	Crystallized cognition	−0.0242	0.0061	6740	−3.98	**< .001**
Within-network FC	Matrix reasoning	−0.0042	0.0050	6609	−0.83	.408
	Fluid cognition	−0.0003	0.0050	6467	−0.06	.951
	Crystallized cognition	−0.0059	0.0051	6449	−1.16	.248

Note: Statistics provided for age by baseline general cognitive ability interactions in whole-brain models of brain development. See Tables S1, S9 and S12 for full output of models with significant age by cognition interactions for matrix reasoning (Wechsler Intelligence Scale for Children), fluid cognition (NIH Toolbox) and crystallized cognition (NIH Toolbox), respectively. Bold indicates uncorrected p < .05.

Fluid cognition at baseline was not associated with cortical thickness development (*p* = .128; Table 2). There were no significant associations of baseline matrix reasoning and crystallized cognition with gray matter structural development (Table 2). After the inclusion of the non-linear age effects (which provided better fit), fluid cognition at baseline was not associated with either linear or non-linear longitudinal development of whole-brain cortical surface area or volume (see Table S4).

**Specificity analyses of gray matter by region.** For surface area, baseline fluid cognition significantly predicted change in surface area over time for three frontal brain regions (rostral middle frontal, pars triangularis, and superior frontal; see Fig. 1b, Table S5). Specifically, those with higher baseline fluid cognition showed a greater decrease in area over time compared to those with lower baseline fluid cognition scores, consistent with the global surface area model. When examining volume regionally, those with higher baseline fluid cognition showed a greater decrease in volume over time for eight frontal (rostral middle frontal, superior frontal, pars orbitalis, caudal middle frontal, caudal anterior cingulate, lateral orbitofrontal) and two temporal (middle temporal, inferior temporal) regions (see Fig. 2b, Table S5), relative to those with lower baseline fluid cognition, which was also consistent with the global volume model. In contrast, change in occipital regions (cuneus, pericalcarine) over time was reduced in those with higher baseline fluid cognition relative to those with lower scores. Results adjusted for total area or volume—available in the Supplement (Table S6, Fig. S2)—showed fewer significant effects. Findings were consistent after adjusting for the number of topological defects calculated from the Euler number (see Tables S7–8).

### 3.3 General cognitive ability in childhood and brain white matter development

**Fig. 4:**
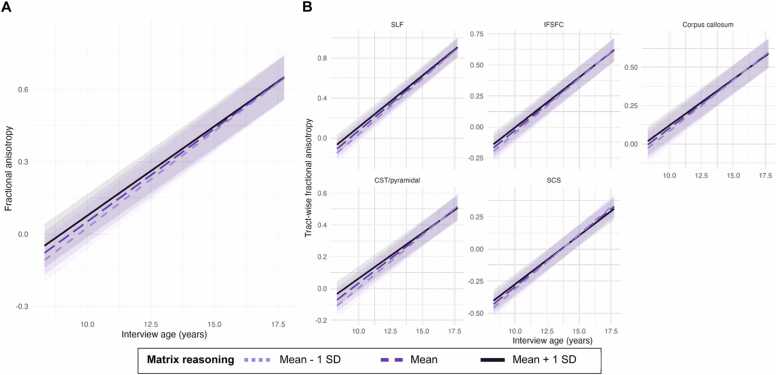
Association between the development of fractional anisotropy (FA) and childhood general cognitive ability indexed by matrix reasoning (Wechsler Intelligence Scale for Children) at the: **A)** whole-brain level and **B)** tract level. Relationships in (B) are displayed for tracts for which longitudinal FA trajectories significantly differed by matrix reasoning. Best fit lines were estimated and displayed for different baseline matrix reasoning values (light purple=mean − 1 SD, dark purple=mean, black=mean + 1 SD). SLF = superior longitudinal fasciculus. IFSFC = inferior frontal superior frontal cortex. CST = corticospinal tract. SCS = superior corticostriate.

**Whole-brain white matter.** Baseline matrix reasoning was significantly associated with FA (*p* = .020), but not MD (*p* = .945), development with age (see Fig. 4a, Table 2). Youth with higher matrix reasoning scores showed a slower increase in average FA (β=0.165, *p* < .001) compared to those with mean (β=0.171, *p* < .001) or lower baseline matrix reasoning (β=0.178, *p* < .001). Cross-sectionally, youth with higher baseline matrix reasoning showed higher FA at baseline, 2-year and 4-year follow up, but not 6-year follow up (Fig. 3, Table S10) relative to those with lower scores.

Findings for fluid (FA: *p* = .946; MD: *p* = .109) and crystallized (FA: *p* = .518; MD: *p* = .236) cognition were not significant. Consistent with primary findings, baseline matrix reasoning was significantly associated with linear FA development with age, but not non-linear age effects (which improved model fit; see Table S4).

**Fractional anisotropy by white matter tract.** Results were significant for five tracts including association (SLF, IFSFC), commissural (CC), and projection (CST/pyramidal, SCS) tracts (see Fig. 4b, Table S11). The effect of baseline matrix reasoning on FA development in these tracts was as in the average FA model (i.e., higher initial FA but slower increase over time in youth with higher baseline matrix reasoning).

### 3.4 General cognitive ability in childhood and functional brain development

**Fig. 5:**
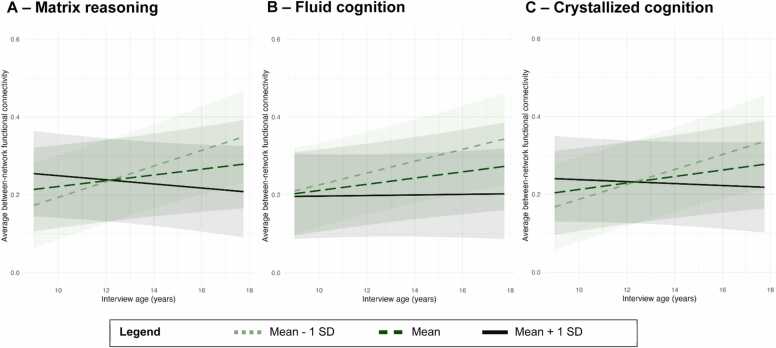
Association between the development of between-network resting-state functional connectivity (rsFC) at the whole-brain level and childhood general cognitive ability indexed by: **A)** matrix reasoning (Wechsler Intelligence Scale for Children), **B)** fluid cognition (NIH Toolbox) and **C)** crystallized cognition (NIH Toolbox). Best fit lines for between-network rsFC development were estimated and displayed for different baseline cognition values (light green=mean − 1 SD, dark green=mean, black=mean + 1 SD). Between-network rsFC standardized prior to analysis (see Fig. S3 for unstandardized plots).

**Whole-brain resting-state functional connectivity.** Scores for all three measures of general cognitive ability at baseline significantly predicted the longitudinal trajectory of whole-brain average between-network rsFC development (*p* < .007; see Fig. 5, Table 2). Children with lower baseline cognition showed a longitudinal increase in average between-network rsFC (range of β=0.032–0.041 for the three cognitive scores, *p* < .002), compared to the minimal average change observed in those with mean (β=0.014–0.016, *p* = .051–.096) and higher baseline cognition (β=−0.001–−0.014, *p* = .180–.959). Cross-sectionally, youth with higher cognitive scores showed less negative between-network rsFC relative to those with lower scores for matrix reasoning only at baseline (Fig. 3, Table S13). In contrast, relative to those with lower childhood scores, youth with higher early fluid cognition showed more negative between-network rsFC at baseline, 2-year and 6-year follow-up, and youth with higher early crystallized cognition had more negative between-network rsFC at 6-year follow-up.

No associations were significant for within-network rsFC development (range *p* = .248–.951). Results including non-linear terms remained consistent with primary findings (with better fit). For all cognitive measures, there were significant associations between cognition and linear age-related changes in between-network rsFC, but no evidence of associations between cognition and quadratic age-related changes in rsFC (see Table S4).

**Fig. 6:**
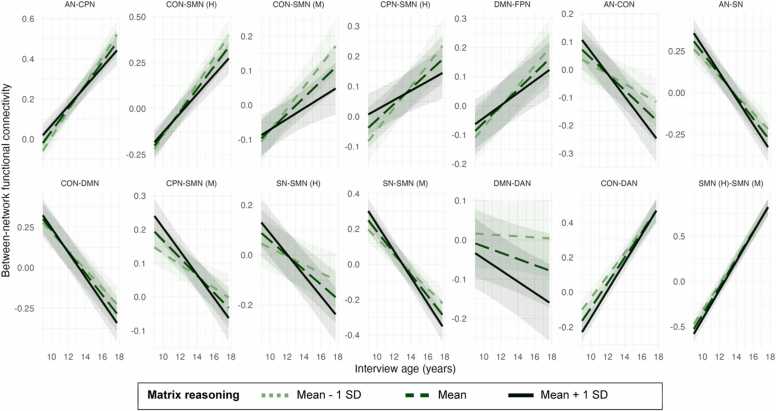
Association between the development of resting-state functional connectivity (rsFC) between pairs of networks and childhood general cognitive ability indexed by matrix reasoning (Wechsler Intelligence Scale for Children). Relationships are displayed for networks pairs between which longitudinal between-network rsFC trajectories significantly differed by cognition. Best fit lines for shown for different baseline matrix reasoning values (light green=mean − 1 SD, dark green=mean, black=mean + 1 SD). Between-network rsFC standardized prior to analysis (see Fig. S4 for unstandardized plots). AN = Auditory network. CON = Cingulo-opercular network. CPN = Cingulo-parietal network. DMN = Default mode network. DAN = Dorsal attention network. FPN = Frontoparietal network. SN = Salience network. SMN (H) = Somatomotor hand network. SMN (M) = Somatomotor mouth network.

**Fig. 7:**
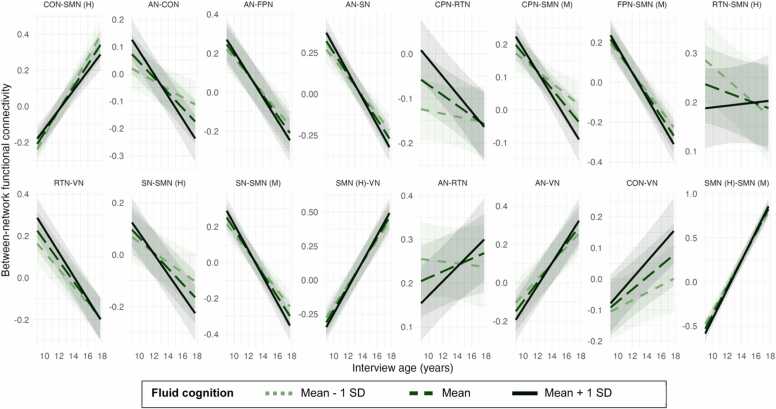
Association between the development of resting-state functional connectivity (rsFC) between pairs of networks and childhood general cognitive ability indexed by indexed by fluid cognition (NIH Toolbox). Relationships are displayed for network pairs between which longitudinal between-network rsFC trajectories significantly differed by cognition. Best fit lines shown for different baseline fluid cognition values (light green=mean − 1 SD, dark green=mean, black=mean + 1 SD). Between-network rsFC standardized prior to analysis (see Fig. S5 for unstandardized plots). AN = Auditory network. CON = Cingulo-opercular network. CPN = Cingulo-parietal network. RTN = Retrosplenial network. SN = Salience network. SMN (H) = Somatomotor hand network. SMN (M) = Somatomotor mouth network.VN = Visual network.

**Fig. 8:**
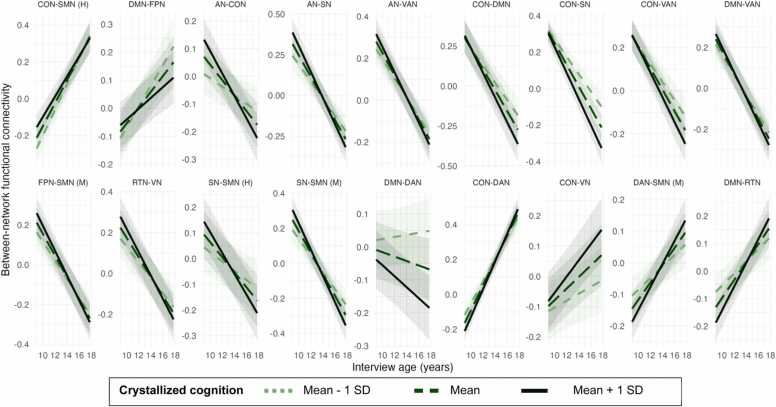
Association between the development of resting-state functional connectivity (rsFC) between pairs of networks and childhood general cognitive ability indexed by indexed by crystallized cognition (NIH Toolbox). Relationships are displayed for network pairs between which longitudinal between-network rsFC trajectories significantly differed by cognition. Best fit lines shown for different baseline crystallized cognition values (light green=mean − 1 SD, dark green=mean, black=mean + 1 SD). Between-network rsFC standardized prior to analysis (see Fig. S6 for unstandardized plots). AN = Auditory network. CON = Cingulo-opercular network. DMN = Default mode network. DAN = Dorsal attention network. FPN = Frontoparietal network. RTN = Retrosplenial network. SN = Salience network. SMN (H) = Somatomotor hand network. SMN (M) = Somatomotor mouth network. VAN = Ventral attention network. VN = Visual network.

**Functional connectivity by network pair.** Between-network rsFC developmental trajectories varied significantly by baseline cognitive ability after FDR correction for matrix reasoning (n = 14 pairs), fluid cognition (n = 16 pairs), and crystallized cognition (n = 18 pairs). Although the patterns were different across the network pairs (see Fig. 6, Fig. 7, Fig. 8, Table S14), one pattern was most consistently observed. For all identified pairs with longitudinal decreases in rsFC over the study period, youth with higher baseline cognitive ability showed more rapid decreases (i.e., became less positive or more negative) in between-network rsFC relative to those with mean or lower baseline cognitive scores. This developmental effect was observed consistently across matrix reasoning, fluid cognition, and crystallized cognition for sensorimotor and sensory (i.e., auditory) networks: AN-CON, AN-SN, CON-SMN (H), SN-SMN (H) and SN-SMN (M). For pairs with normative increases in between-network rsFC, higher matrix reasoning was most commonly associated with slower increases for some pairs, but higher fluid and crystallized cognition with faster increases (for other network pairs) compared to youth with mean or lower baseline cognitive ability.

### 3.5 Sex differences in associations between early cognitive ability and brain development

No differences between sexes were found in the association between childhood cognitive measures and brain development (range *p* = .328–.847; see Tables S15–17).

### 3.6 Sensitivity analysis adjusting for different socioeconomic indicators

Findings of associations between early cognition and brain development were unchanged when adjusting for average parent educational attainment or neighborhood disadvantage at baseline instead of income-to-needs ratio in global models; associations at the regional level were also largely consistent with primary findings (see Tables S22–27).

## 4 Discussion

In this exploratory study, we examined the association of general cognitive ability measured in childhood with longitudinal changes in brain structure and function from early to middle adolescence. Distinct cognitive measures were associated with longitudinal changes in structural measures; gray matter structural area and volume were associated with early fluid cognition, and white matter microstructure related to childhood matrix reasoning. Between-network rsFC development related to early cognitive ability across all measures—matrix reasoning, fluid cognition, and crystallized cognition. Across modalities, higher baseline cognitive scores were associated with slower increases in measures that normatively increased over adolescence and faster decreases for those that were found to decline.

Composite fluid cognition measured in childhood predicted differences in whole-brain average gray matter area and volume trajectories. Higher childhood fluid cognition was associated with more rapid decreases in global surface area and volume over the studied age period, compared to youth with lower early fluid cognition. Observed associations with early cognition were likely driven by surface area changes, given evidence that volume may share significantly more variance with area than thickness (Winkler et al., 2010). Our findings are consistent with prior work which used a composite cognitive measure (Schnack et al., 2015). In the context of the specific abilities captured by the fluid cognition measure in the NIH Toolbox (Jewsbury et al., 2016, Jewsbury et al., 2017), this suggests that childhood fluid reasoning, working memory, and processing speed abilities may be more strongly linked to proceeding changes in brain structure.

At the regional level, higher fluid cognition was associated with accelerated age-related decreases in the area and volume of frontal regions, particularly rostral middle frontal and superior frontal. These prefrontal regions have been implicated in higher-order functioning (Jung and Haier, 2007, Yuan and Raz, 2014), and developmental change in their morphology has been shown to be associated with cognitive ability (Schmitt et al., 2019, Shaw et al., 2006, Sowell et al., 2004; although see null findings in Estrada et al., 2019). Volumetric changes in the middle and inferior temporal regions also related to early fluid cognition, likely due to their involvement in memory processes (Ranganath and Ritchey, 2012). After periods of cortical growth in childhood, cortical morphogenesis shifts to a period of refinement in adolescence (e.g., synaptic pruning, dendritic simplification) (Juraska and Drzewiecki, 2020). Animal models have found that enriched environments and cognitive stimulation can advance these refinement processes via changes in neurotrophic factors, activity-dependent signaling and microglial pathways (Bian et al., 2015, Cornell et al., 2022, Novkovic et al., 2015). Children with higher cognitive ability may receive more cognitive stimulation from parents (Starr et al., 2024), which may contribute to a faster pace of neurodevelopment in adolescence. An alternative explanation for our findings is residual confounding. That is, although we adjusted for family income, and findings were largely consistent when adjusting for parent educational attainment or neighborhood disadvantage, early cognitive ability and rates of adolescent brain development may both be linked by a common underlying factor, such as genetics or specific neighborhood factors including access to green spaces (Hackman et al., 2021; Q. Li et al., 2025; Williams et al., 2023). Future longitudinal work should examine the influence of factors including genetics, cognitive stimulation and greenspace on the link between early cognitive ability and structural brain development.

Cortical thickness development did not relate to early cognitive ability, which stands in contrast to some prior studies (Schnack et al., 2015, Shaw et al., 2006). Several factors may be at play. First, our study distinguished between fluid and crystallized cognition as assessed by the NIH Toolbox while prior work examined an overall composite cognitive measure. Second, our models adjusted for family income, given evidence that socioeconomic factors shape both brain and cognitive development (Lawson et al., 2018, Rakesh and Whittle, 2021, Rosen et al., 2025, Tsomokos et al., 2026) while prior work did not. Notably, the cognitive subgroups in Shaw et al. (2006) differed in socioeconomic status, which may have contributed to the observed associations. Third, while ABCD cohort cognitive abilities may more closely match the general population, in earlier studies there was a heavy over-representation of those with above average general cognitive ability. Finally, ABCD differed from earlier studies in the scanner strength used (3 T versus 1.5 T) and the age windows covered differed, with a wider age range in earlier work affording a non-linear approach (e.g., from ∼4–29 in Shaw et al., 2006), compared to the window in ABCD of 8–18 years which meant a linear fit was appropriate. Further studies in samples maintaining a range in SES and cognitive abilities, but with an expanded age range, may consolidate findings.

While there has been prior work on longitudinal gray matter structural development, to our knowledge this is the first study to characterize associations between early cognition and longitudinal development of white matter microstructure and rsFC development. For white matter, higher childhood matrix reasoning was associated with slower increases in whole-brain FA compared to those with lower initial matrix reasoning. This extends prior longitudinal work in early childhood which suggests that higher childhood intelligence may be associated with a protracted trajectory of white matter development (Deoni et al., 2016), likely due to slower myelination and synaptogenesis in early life, followed by a prolonged period of synaptic pruning and myelination. Our findings suggest that this pattern extends to adolescence. Results were specific to matrix reasoning and were not observed for composite measures of crystallized or fluid cognition. This may be due to crystallized cognition relying on more localized white matter connectivity compared to matrix reasoning (Gazes et al., 2020). However, the absence of associations between fluid cognition and white matter development was surprising. There may be specific tracts associated with different dimensions of the fluid cognition composite (e.g., working memory, processing speed), which can be examined in further work (Fuhrmann et al., 2020). At the tract level, early matrix reasoning was associated with differences in FA trajectories of the superior longitudinal fasciculus corticospinal tract and corpus callosum, as highlighted in prior cross-sectional work (Tamnes et al., 2010), and also for the fronto-striatal/frontal association pathways. Although structurally connecting different regions, all these pathways are involved in processes including executive, language and/or motor function (Forkel et al., 2022, Ribeiro et al., 2024). Together, these findings suggest that early matrix reasoning skills may be linked with protracted global white matter development, with preliminary evidence showing that effects may be driven by tracts relevant for cognitive and motor domains. In contrast to FA, there were no significant associations for MD, which may be explained by slower changes in MD over childhood and adolescence (Lebel et al., 2019). Future work examining specific tracts and earlier developmental periods—when MD changes are more rapid—may yield different patterns.

Fluid cognition, crystallized cognition, and matrix reasoning in childhood were all associated with longitudinal increases in whole-brain between-network rsFC, but not within-network rsFC. For all cognitive measures, those with lower childhood cognitive ability showed a longitudinal increase in global between-network rsFC that was absent in youth with higher early cognitive ability. Findings align with recent cross-sectional adult work suggesting that general cognitive ability may be best predicted by global FC patterns, particularly between-network rsFC rather than within-network (Anderson and Barbey, 2023, Wilcox and Barbey, 2023). Furthermore, we found that associations between early cognition and functional trajectories were stronger than for structural measures, consistent with prior cross-sectional work (Ooi et al., 2022), and suggesting that between-network rsFC changes may be more sensitive to early cognition.

Observing a longitudinal increase in global between-network rsFC only in those with lower childhood cognitive ability was unexpected, though does not preclude changes in rsFC between specific networks. Indeed, when exploring the pairs of networks showing associations between early cognitive ability and rsFC trajectories, although there was variability across network pairs, we observed a general pattern where those with higher early cognitive ability frequently showed faster decreases in between-network rsFC for networks showing normative rsFC weakening over adolescence. In particular, connectivity between the auditory–salience, auditory–cingulo-opercular, and salience–somatomotor networks decreased over the studied period and, for all cognitive measures, the coupling between networks reduced at a faster rate for those with higher vs lower early cognitive ability. The salience and cingulo-opercular networks are linked with cognitive control and attention processes (Dosenbach et al., 2025) and their integration with other networks may decrease over development to facilitate their distinct functions (e.g., attention shifting vs attention maintenance); our findings suggest that higher childhood general cognitive ability may be linked to an acceleration in this developmental process. In addition, higher early crystallized cognition and matrix reasoning were associated with more rapid increase in negative default mode rsFC with the dorsal attention and cingulo-opercular networks, and slower increases in positive default mode rsFC with the frontoparietal network. This resulted in greater decoupling between the default mode and higher-order networks by adolescence in those with higher relative to lower cognitive ability, consistent with adult studies (Avelar-Pereira et al., 2017, Keller et al., 2015). It is unclear why a composite measure of early fluid cognition as captured by the NIH Toolbox was not also associated with default mode to higher-order network rsFC; future work can examine whether any specific abilities (e.g., working memory) show such associations.

Consolidating findings across brain markers, higher early cognitive ability was linked to accelerated decreases in global area and volume, but reduced increases in FA and between-network rsFC. Whilst acknowledging the exploratory nature of these analyses, this supports prior structural findings that early cognitive ability may be associated with a protracted growth period, followed by sharper decrease in brain measures (Deoni et al., 2016, Shaw et al., 2006), and theories that higher cognitive ability may be associated with an extended sensitive period of development (Brant et al., 2013). However, genetics, cognitive stimulation, and environmental factors are all possible drivers of extended windows of plasticity, which likely interact during development through processes such as epigenetics (Lenroot and Giedd, 2011). Further work examining the contributions of these factors is needed to establish causal mechanisms linking cognition, brain development and later outcomes.

This study had several strengths including the large sample of longitudinal neuroimaging data and reliable cognitive measures (Heaton et al., 2014), however there are some limitations to note. First, this study focused on whole-brain average measures and followed up significant effects at the local level. This was motivated by evidence that whole-brain measures have increased signal-to-noise ratio and can overcome some between-scanner variability compared to regional measures (Knussmann et al., 2022). Nevertheless, this may have overlooked effects specific to particular regions or networks which can be examined in future work. Second, imaging data at the 6-year follow-up was only partly available and thus we prioritized linear modeling for primary analyses and completed sensitivity analyses using non-linear models. This showed that associations between early fluid cognition and global area and volume changes were significant only in the linear model form. There is substantial variability between studies, but evidence suggests that area and volume could peak between 8–12 years old (Kelly et al., 2024, Norbom et al., 2021). Future longitudinal studies with sufficient timepoints prior to 8 years old are necessary to fully model non-linear developmental effects and confirm findings. Third, household socioeconomic status—associated both with cognition and brain development—has been linked to attrition in the ABCD study (Ewing et al., 2022, Rakesh et al., 2025), potentially biasing estimates. Fourth, despite adjusting for confounds such as income, there is potential residual confounding from other factors not examined including genetics, social and physical environmental factors, or psychopathology (Buczyłowska et al., 2023; Q. Li et al., 2025; McLaughlin et al., 2019; Rakesh, Elzeiny, et al., 2023; Schäfer et al., 2023; Williams et al., 2023); these factors constitute key areas of interest for future research. Fifth, although some mechanisms have been proposed to explain the association between early cognitive ability and brain development (Brant et al., 2013, Deoni et al., 2016, Lenroot and Giedd, 2011), future longitudinal work is needed to test these possible pathways. Sixth, there are multiple facets of cognitive ability ranging from fluid reasoning, comprehension-knowledge and processing speed to auditory processing (McGrew, 2009). We focused on a subset of these in this preliminary work, using composite scores to capture broader high-level effects. Future work using multiple targeted measures of each specific ability (McGrew and Wendling, 2010) would allow these effects to be studied at a more granular level better aligned with cognitive theory*.* Seven, while the present study adjusted for family income given its established association with both cognitive ability and brain development (Farah, 2017, Hu and Farah, 2026, Rakesh et al., 2023), evidence also suggests that SES moderates the relationship between cognitive abilities and the brain (Hu and Farah, 2026). Future work should therefore examine whether the relationship between cognitive ability and change in brain structure and function differs as a function of SES. Finally, this study did not examine the temporal coupling of cognitive and brain trajectories, which is an important direction for future work as it could shed light on shared developmental pathways.

In conclusion, early general cognitive ability predicted rates of longitudinal structural and functional brain development. Higher general cognitive ability in childhood was associated with slower growth rates for brain measures that normatively increased over adolescence, and more rapid decreases for brain measures typically showing an adolescent decline. Findings may be related to brain-sculpting effects of early cognitive stimulation and learning, or shared underlying factors (e.g., genetics) influencing both early cognition and later brain maturation. Future work is needed to shed light on possible mechanisms linking brain and cognitive features.

## CRediT authorship contribution statement

**Divyangana Rakesh:** Writing – review & editing, Writing – original draft, Supervision, Methodology, Funding acquisition, Conceptualization. **Phoebe Thomson:** Writing – review & editing, Writing – original draft, Visualization, Methodology, Formal analysis, Data curation, Conceptualization. **Philip Shaw:** Writing – review & editing.

## Financial disclosures

The authors report no biomedical financial interests or potential conflicts of interest.

## Declaration of Competing Interest

The authors declare that they have no known competing financial interests or personal relationships that could have appeared to influence the work reported in this paper.

## Data Availability

The authors do not have permission to share data. Data used in the preparation of this article were obtained from the Adolescent Brain Cognitive Development (ABCD) Study (https://abcdstudy.org), held in the NIH Brain Development Cohorts (NBDC) Data Sharing Platform, which is available for authorized users with approved Data Use Certification (DUC). The data used in this project are publicly available (https://abcdstudy.org/). Access to the data is granted to qualified researchers via a data use agreement. For further information on how to obtain access to this dataset, visit the NIH Brain Development Cohorts data sharing platform (https://www.nbdc-datahub.org/).
