# Supplementary material for: Cognitive ability in childhood predicts adolescent structural and functional brain development: A longitudinal study
Source: Dev Cogn Neurosci. 2026 Jun 18;80:101768. doi: 10.1016/j.dcn.2026.101768 (PMC13315900; doi:10.1016/j.dcn.2026.101768)
Supplement: Figure S1 — Supplementary material [file mmc1.docx]

**Cognitive ability in childhood predicts adolescent structural and functional brain development: A longitudinal study**

**Supplementary materials**

Table of Contents

[Additional participant information 4](#_Toc229474130)

[Data collection and preprocessing 4](#_Toc229474131)

[Formulae for statistical models 6](#_Toc229474132)

[Exploratory analyses 7](#_Toc229474133)

[Supplementary results 8](#_Toc229474134)

[Table S1. Interaction between age and baseline fluid cognition in whole brain measures of brain gray matter structural development 10](#_Toc229474135)

[Table S2. Simple slopes for global brain development by baseline general cognitive ability 12](#_Toc229474136)

[Table S3. Cross-sectional differences in average gray matter structure for youth with higher vs lower baseline general cognitive ability 13](#_Toc229474137)

[Table S4. Non-linear models of whole brain measures of brain development and associations with baseline cognitive ability 14](#_Toc229474138)

[Table S5. Interaction between age and baseline fluid cognition in models of gray matter structural development by region 16](#_Toc229474139)

[Table S6. Interaction between age and baseline fluid cognition in models of gray matter structural development by region, controlling for whole-brain total estimates 17](#_Toc229474140)

[Table S7. Interaction between age and baseline fluid cognition in global models of gray matter structural development, controlling for Euler number–derived topology defects 19](#_Toc229474141)

[Table S8. Interaction between age and baseline fluid cognition in global models of gray matter structural development by region, controlling for Euler number–derived topology defects 21](#_Toc229474142)

[Table S9. Interaction between age and baseline matrix reasoning scaled score in whole brain measures of brain white matter development 22](#_Toc229474143)

[Table S10. Cross-sectional differences in average fractional anisotropy for youth with higher vs lower baseline general cognitive ability 23](#_Toc229474144)

[Table S11. Interaction between age and baseline matrix reasoning in models of fractional anisotropy development by bilateral white matter tract 24](#_Toc229474145)

[Table S12. Interaction between age and baseline general cognitive ability in whole brain measures of brain functional connectivity development 25](#_Toc229474146)

[Table S13. Cross-sectional differences in average between-network resting-state functional connectivity for youth with higher vs lower baseline general cognitive ability 27](#_Toc229474147)

[Table S14. Interaction between age and baseline general cognitive ability in models of between-network resting-state functional connectivity development by network pair 28](#_Toc229474148)

[Table S15. Interaction between age, baseline fluid cognition and sex in whole brain gray matter structural development 32](#_Toc229474149)

[Table S16. Interaction between age, baseline matrix reasoning and sex in whole brain white matter structural development 34](#_Toc229474150)

[Table S17. Interaction between age, baseline general cognitive ability and sex in whole brain between-network functional connectivity development 35](#_Toc229474151)

[Table S18. Models of whole brain measures of brain development and associations with baseline cognitive ability, after site exclusion 37](#_Toc229474152)

[Table S19. Interaction between age and baseline fluid cognition in models of gray matter structural development by region, after site exclusion 38](#_Toc229474153)

[Table S20. Interaction between age and baseline matrix reasoning in models of fractional anisotropy development by bilateral white matter tract, after site exclusion 40](#_Toc229474154)

[Table S21. Interaction between age and baseline general cognitive ability in models of between-network resting-state functional connectivity development by network pair, after site exclusion 41](#_Toc229474155)

[Table S22. Interaction between age and baseline fluid cognition in models of gray matter structural development by region, adjusted for average parent educational attainment 45](#_Toc229474156)

[Table S23. Interaction between age and baseline fluid cognition in models of gray matter structural development by region, adjusted for neighborhood disadvantage 47](#_Toc229474157)

[Table S24. Interaction between age and baseline matrix reasoning in models of fractional anisotropy development by bilateral white matter tract, adjusted for average parent educational attainment 49](#_Toc229474158)

[Table S25. Interaction between age and baseline matrix reasoning in models of fractional anisotropy development by bilateral white matter tract, adjusted for neighborhood disadvantage 50](#_Toc229474159)

[Table S26. Interaction between age and baseline general cognitive ability in models of between-network resting-state functional connectivity development by network pair, adjusted for average parent educational attainment 51](#_Toc229474160)

[Table S27. Interaction between age and baseline general cognitive ability in models of between-network resting-state functional connectivity development by network pair, adjusted for neighborhood disadvantage 55](#_Toc229474161)

[Figure S1. Participant flow diagram. 59](#_Toc229474162)

[Figure S2. Interaction between age and baseline fluid cognition in models of surface area and volume development by region, controlling for whole-brain total estimates. 60](#_Toc229474163)

[Figure S3. Associations between cognition and global between-network rsFC development (unstandardized). 61](#_Toc229474164)

[Figure S4. Associations between matrix reasoning and pairwise between-network rsFC development (unstandardized). 62](#_Toc229474165)

[Figure S5. Associations between fluid cognition and pairwise between-network rsFC development (unstandardized). 63](#_Toc229474166)

[Figure S6. Associations between crystallized cognition and pairwise between-network rsFC development (unstandardized). 64](#_Toc229474167)

# Additional participant information

A total of 10,495 participants were included in analyses. These participants had: 1) complete information available at baseline for at least one measure of general cognitive ability; 2) sex, study site, household income and household size at baseline; 3) at least one time point with usable neuroimaging data (structural MRI [sMRI], resting-state functional MRI [rsfMRI] and/or diffusion MRI [dMRI]). Usable neuroimaging data was based on the recommended inclusion flag for images passing quality control criteria developed by the ABCD Data Analysis, Informatics & Resource Center (DAIRC). These criteria included, but were not limited to, the following: visual inspection of the raw image, checks for the completeness of preprocessing (e.g., in FreeSurfer), visual inspection of post-processed data (e.g., quality of registration), and success in obtaining derived brain metrics (see Hagler et al., 2019). For rsfMRI, there was an additional check that at least 375 frames remained after motion censoring. For most included participants (n=10133, 96.56%), the first time point with neuroimaging data passing quality control was at baseline; for a small number of participants the first time point with passing neuroimaging data was at 2-year (n=260, 2.48%), 4-year (n=81, 0.77%), or 6-year (n=21, 0.20%) follow up. On average, participants had useable data for 2.56 (± 0.99) time points. When separated by different cognitive measures at baseline, a total of 10,297 participants had Wechsler Intelligence Scale for Children (WISC) V - Matrix Reasoning scaled scores, 10,164 participants had NIH Toolbox Fluid composite age-corrected standard scores, and 10,206 participants had NIH Toolbox Crystallized composite age-corrected standard scores.

# Data collection and preprocessing

Complete details of MRI scanning and preprocessing procedures for the ABCD study have been documented (Casey et al., 2018; Hagler et al., 2019). Below we provide a summary of the key imaging parameters and preprocessing steps conducted by the ABCD Data Analysis, Informatics & Resource Center (DAIRC) prior to ABCD data release.

***Gray matter structure (whole-brain and region-wise)***

**Data collection and preprocessing.** sMRI images were acquired using T1-weighted magnetization-prepared rapid acquisition gradient echo scans with 1.0 mm isotropic resolution. Structural T1 images underwent preprocessing including distortion correction, resampling and cortical surface reconstruction using FreeSurfer (i.e., skull-stripping, white matter segmentation, surface optimization and nonlinear registration to spherical surface-based atlas). Cortical thickness, area and volume were estimated for each region in the Desikan-Killiany atlas (Desikan et al., 2006). Values across all atlas regions were averaged yielding *whole-brain average cortical thickness*, *whole-brain average surface area* and *whole-brain average cortical volume.*

**Region-wise area, volume and thickness calculation**. For each region, we averaged area, volume and thickness values for in the left and right hemisphere to provide bilateral regional estimates. This yielded *region-wise average cortical thickness*, *region-wise average surface area,* and *region-wise average* volume for 34 brain regions: banks of superior temporal sulcus, caudal anterior cingulate, caudal middle frontal, cuneus, entorhinal, fusiform, isthmus cingulate, insula, inferior parietal, inferior temporal, lingual, lateral orbitofrontal, lateral occipital, medial orbitofrontal, middle temporal, paracentral, pericalcarine, posterior cingulate, frontal pole, parahippocampal, pars orbitalis, postcentral, pars opercularis, precuneus, precentral, pars triangularis, temporal pole, rostral anterior cingulate, rostral middle frontal, superior frontal, supramarginal, superior parietal, superior temporal, and transverse temporal.

***White matter metrics (whole-brain and tract-wise)***

**Data collection and preprocessing*.*** dMRI images were acquired using a multi-shell protocol including b=500, b=1000, b=2000, and b=3000, with 96 diffusion directions and 1.7mm^3^ resolution. Preprocessing of dMRI images included eddy current correction, head motion correction, distortion correction and resampling. Diffusion tensor imaging analysis using all gradient strengths and directions (i.e., full shell) was then conducted. White matter tracts were labelled using AtlasTrack (Hagler et al., 2009), and weighted average fractional anisotropy (FA) and mean diffusivity (MD) were calculated across all tracts, yielding measures of *whole-brain average FA,* and *whole-brain average MD*. Weighted FA and MD were also calculated for each AtlasTrack tract separately.

**Tract-wise fractional anisotropy and mean diffusivity calculation.** For projection and association tracts, we averaged FA and MD values for the left and right of each tract to provide a single bilateral tract estimate. This yielded *tract-wise average FA* and *tract-wise average MD* values for 15 white matter tracts: corpus callosum (CC), forceps major, forceps minor, anterior thalamic radiations, cingulum (cingulate), cingulum (parahippocampal), corticospinal tract/pyramidal tract (CST), fornix, inferior fronto-occipital fasciculus, inferior frontal superior frontal cortex (IFSFC), inferior longitudinal fasciculus, superior corticostriate (SCS), striatal inferior frontal cortex, superior longitudinal fasciulus (SLF), and uncinate fasciculus. Note: For tracts defined in AtlasTrack both as a whole (e.g., SLF) and in parts (e.g., parietal SLF and temporal SLF), only the full tracts were used.

***Resting-state functional connectivity (whole-brain and network-wise)***

**Data collection and preprocessing.** Participants completed 4–5 rsfMRI scans (eyes open; 5 minutes per scan) to allow for a minimum of 8 minutes of low-motion data. rsfMRI data were preprocessed including normalization, head motion correction, temporal filtering and projection onto the cortical surface. The Gordon parcellation (Gordon et al., 2016) was used to define 333 brain regions, belonging to one of 12 functional networks: auditory (AN), cingulo opercular (CON), cingulo parietal (CPN), dorsal attention (DAN), default mode (DMN), frontoparietal (FPN), retrosplenial temporal (RTN), sensorimotor hand (SMN [H]), sensorimotor mouth (SMN [M]), salience (SN), ventral attention (VAN), and visual (VN). The timeseries correlation between each pair of regions was calculated and Fisher transformed to represent region-wise resting-state functional connectivity (rsFC). *Pairwise between-network rsFC* was estimated for each possible network pair as the average rsFC between all pairs of regions belonging the two networks, resulting in between-network rsFC for 66 unique network pairs. *Network-wise average within-network rsFC* was estimated as rsFC between all pairs of regions within a network, resulting in within-network rsFC for 12 networks.

**Functional connectivity calculation.** Using these network-wise measures, we calculated *whole-brain average between-network rsFC* and *whole-brain average within-network rsFC.* Whole-brain average between-network rsFC was estimated as the mean across the 66 pairwise average between-network rsFC values. Whole-brain average within-network rsFC was calculated as the mean across the 12 network-wise average within-network rsFC values.

# Formulae for statistical models

For primary analyses, linear mixed effects models (LMMs) were defined by the following formula: *Brain measure ~ Age × Cognitive ability + Age × Sex + Age × INR + MRI model + Mean FD + (Age | ID) + (1 | Site) + (1 | Family ID). Note: INR = income-to-needs ratio, FD = framewise displacement*. For exploratory analyses testing whether the relationship between *Cognitive ability* and brain development varied by sex, LMMs followed the formula: *Brain measure ~ Age × Cognitive ability × Sex + Age × INR + MRI model + Mean FD + (Age | ID) + (1 | Site) + (1 | Family ID).* Finally, sensitivity analyses examining non-linear brain development trajectories with age used LMMs of the form: *Brain measure ~ Age^2^ × Cognitive ability + Age^2^ × Sex + Age^2^ × INR + Age × Cognitive ability + Age × Sex + Age × INR + MRI model + Mean FD + (Age | ID) + (1 | Site) + (1 | Family ID)*. Across all formulae, mean FD was only available for dMRI or rsfMRI models and thus excluded as a covariate in sMRI models, as described in the main text. However, sensitivity analyses were run on sMRI models additionally including the number of topological defects calculated from Euler number as a covariate (see results below).

# Exploratory analyses

***Simple slopes analysis***

For any significant interaction effects identified between cognitive scores and age in predicting brain measures, simple slopes analysis was conducted using the *interactions* package (Long, 2019). This examined conditional effects of age on brain measures at different levels of cognitive ability. Uncorrected p-values were reported in the text with p<0.05 considered significant, but false discovery rate (FDR) corrected p-values by modality are provided in Table S2.

***Cross-sectional differences***

To ascertain at what ages associations between global brain measures and baseline cognitive ability were present, we followed up significant longitudinal effects for global measures with exploratory cross-sectional analyses. These analyses tested differences between youth with lower and higher baseline cognitive ability in average brain measures at each time point. Participants were separated into lower and higher baseline cognition groups using a median split. Participants with baseline cognitive ability equal to the median were randomly sorted and then assigned to the lower or higher baseline cognition group to ensure balanced group sizes. LMMs were conducted in each timepoint separately, testing for a main effect of baseline cognition group on global brain measures. Models included covariates for age, sex, INR, scanner model and mean FD, and random intercepts for study site and family ID, with the formula: *Brain measure ~ Cognition group + Age + Sex + INR + MRI model + Mean FD + (1 | Site) + (1 | Family ID).* For these exploratory analyses, uncorrected p < .05 was considered significant.

***Sensitivity analysis adjusting for different socioeconomic indicators***

Sensitivity analyses reran all models using average parent educational attainment or neighborhood disadvantage at baseline as the socioeconomic covariate instead of INR. Parent educational attainment at baseline was operationalized as the average educational attainment across the primary and secondary caregiver in years (or by the educational attainment of one caregiver if only one caregiver was available). Neighborhood disadvantage at baseline was indexed by the area deprivation index (Kind et al., 2014; Singh, 2003), a composite measure based on 17 sub-scores of local family income, education, employment, and home value and quality. Area deprivation index national percentile values for each participant were estimated using their primary address, and then subtracted from 101, yielding a measure where lower values represent higher disadvantage.

# Supplementary results

***Baseline fluid cognition and regional gray matter structural development relative to whole-brain estimates***

Regional area and volume results varied after controlling for global area and volume in their respective models. For surface area, no associations for frontal regions remained significant, however age-related changes in three occipital regions were significantly associated with baseline fluid cognition independent of total brain area (see Table S6 and Figure S2). Associations between baseline fluid cognition and change in volume with age remained present for the rostral middle frontal, middle temporal, pericalcarine and cuneus regions; for other regions (predominantly frontal) the relationships between baseline fluid cognition and volume decreases over time were no longer significant.

***Baseline fluid cognition and regional gray matter structural development additionally controlling for Euler number-derived topology defects***

After additionally controlling for the number of topological defects calculated from the Euler number in FreeSurfer, all associations between baseline fluid cognition and development of surface area and volume were consistent with main findings, at both the global and regional level (see Table S7 and S8).

***Cognitive ability and brain development associations after exclusion of smallest study site***

As one site (Icahn School of Medicine at Mount Sinai) included < 30 participants after exclusions and did not participate longitudinally, analyses were repeated after the removal of data from this site. All associations between early cognitive ability and global brain development remained consistent with main findings after this site exclusion (see Table S18). For surface area and volume, all associations between fluid cognition and regional brain development remained significant except for surface area in the superior frontal region (see Table S19). For dMRI, all associations between baseline matrix reasoning and fractional anisotropy were significant after site exclusion (see Table S20). For rsfMRI, associations between baseline cognition and change in between-network rsFC with age remained present for all network pairs except default mode network-dorsal attention network rsFC for matrix reasoning (see Table S21).

# Table S1. Interaction between age and baseline fluid cognition in whole brain measures of brain gray matter structural development

| **MRI measure** | **Cognitive measure** | **Variable** | ***β*** | ***SE*** | ***df*** | ***t*** | ***p*** |
| --- | --- | --- | --- | --- | --- | --- | --- |
| Cortical surface area | Fluid cognition | Intercept | 0.3690 | 0.0283 | 26 | 13.06 | <.001 |
|  |  | Age | -0.0441 | 0.0014 | 6833 | -32.05 | <.001 |
|  |  | Cognitive ability | 0.0758 | 0.0083 | 9677 | 9.16 | <.001 |
|  |  | Sex (Female vs Male) | -0.9866 | 0.0161 | 9964 | -61.18 | <.001 |
|  |  | Baseline INR | 0.1722 | 0.0094 | 8223 | 18.41 | <.001 |
|  |  | **Age x Cognitive ability** | **-0.0033** | **0.0010** | **6363** | **-3.29** | **.001** |
|  |  | Age x Sex | -0.0585 | 0.0019 | 6423 | -30.20 | <.001 |
|  |  | Age x Baseline INR | 0.0040 | 0.0010 | 6501 | 4.01 | <.001 |
|  |  | Achieva dStream* | -0.1982 | 0.0355 | 909 | -5.59 | <.001 |
|  |  | DISCOVERY MR750* | 0.4260 | 0.0193 | 2180 | 22.09 | <.001 |
|  |  | Ingenia* | -0.2544 | 0.0461 | 1154 | -5.52 | <.001 |
|  |  | MAGNETOM Prisma* | -0.0599 | 0.0267 | 12540 | -2.24 | .025 |
|  |  | MAGNETOM Prisma Fit* | -0.0710 | 0.0105 | 12860 | -6.76 | <.001 |
|  |  | Orchestra SDK | 0.5107 | 0.1564 | 15140 | 3.27 | .001 |
|  |  | Prisma* | 0.0300 | 0.0061 | 14500 | 4.93 | <.001 |
|  |  | SIGNA Premier* | 0.3674 | 0.0215 | 3062 | 17.10 | <.001 |
|  |  | SIGNA UHP* | 0.3408 | 0.0214 | 3045 | 15.96 | <.001 |
| Cortical volume | Fluid cognition | Intercept | 0.3730 | 0.0345 | 24 | 10.81 | <.001 |
|  |  | Age | -0.2396 | 0.0017 | 7037 | -142.80 | <.001 |
|  |  | Cognitive ability | 0.0676 | 0.0079 | 9501 | 8.52 | <.001 |
|  |  | Sex (Female vs Male) | -0.9282 | 0.0155 | 9879 | -59.89 | <.001 |
|  |  | Baseline INR | 0.2031 | 0.0091 | 8578 | 22.36 | <.001 |
|  |  | **Age x Cognitive ability** | **-0.0026** | **0.0012** | **6555** | **-2.12** | **0.03** |
|  |  | Age x Sex | -0.0925 | 0.0024 | 6621 | -39.13 | <.001 |
|  |  | Age x Baseline INR | 0.0043 | 0.0012 | 6705 | 3.47 | <.001 |
|  |  | Achieva dStream* | -0.2022 | 0.0430 | 998 | -4.70 | <.001 |
|  |  | DISCOVERY MR750* | 0.1373 | 0.0235 | 2436 | 5.85 | <.001 |
|  |  | Ingenia* | -0.1224 | 0.0560 | 1229 | -2.18 | 0.03 |
|  |  | MAGNETOM Prisma* | -0.0449 | 0.0326 | 12560 | -1.38 | 0.17 |
|  |  | MAGNETOM Prisma Fit* | -0.0436 | 0.0128 | 12930 | -3.40 | 0.00 |
|  |  | Orchestra SDK | 0.0414 | 0.1921 | 15240 | 0.22 | 0.83 |
|  |  | Prisma* | 0.0408 | 0.0074 | 14710 | 5.49 | <.001 |
|  |  | SIGNA Premier* | 0.1950 | 0.0261 | 3253 | 7.46 | <.001 |
|  |  | SIGNA UHP* | 0.1009 | 0.0260 | 3298 | 3.88 | <.001 |

Note. Bold indicates age x cognitive ability interaction of primary interest, where general cognitive ability at baseline was indexed by NIH Toolbox Fluid cognition scores. * Estimates are shown for each MRI model type compared to the Prisma Fit model as the reference group. INR = income-to-needs ratio.

# Table S2. Simple slopes for global brain development by baseline general cognitive ability

| **Modality** | **Cognitive measure** | **Condition** | ***Estimate*** | ***SE*** | ***t*** | ***p*** | ***p_FDR_*** |
| --- | --- | --- | --- | --- | --- | --- | --- |
| ***Gray matter structure*** |  |  |  |  |  |  |  |
| Surface area | Fluid cognition | Mean - 1 SD | -0.041 | 0.002 | -24.40 | **<.001** | **<.001** |
|  |  | Mean | -0.044 | 0.001 | -32.20 | **<.001** | **<.001** |
|  |  | Mean + 1 SD | -0.048 | 0.002 | -27.73 | **<.001** | **<.001** |
| Volume | Fluid cognition | Mean - 1 SD | -0.237 | 0.002 | -115.76 | **<.001** | **<.001** |
|  |  | Mean | -0.240 | 0.002 | -142.92 | **<.001** | **<.001** |
|  |  | Mean + 1 SD | -0.242 | 0.002 | -115.85 | **<.001** | **<.001** |
| ***White matter*** |  |  |  |  |  |  |  |
| Fractional anisotropy | Matrix | Mean - 1 SD | 0.178 | 0.005 | 38.27 | **<.001** | **<.001** |
|  |  | Mean | 0.171 | 0.004 | 45.06 | **<.001** | **<.001** |
|  |  | Mean + 1 SD | 0.165 | 0.005 | 35.01 | **<.001** | **<.001** |
| ***Functional connectivity*** | |  |  |  |  |  |  |
| Between-network functional connectivity | Matrix reasoning | Mean - 1 SD | 0.041 | 0.010 | 4.11 | **<.001** | **<.001** |
|  |  | Mean | 0.014 | 0.008 | 1.66 | .096 | .144 |
|  |  | Mean + 1 SD | -0.014 | 0.010 | -1.34 | .180 | .231 |
|  | Fluid cognition | Mean - 1 SD | 0.032 | 0.010 | 3.16 | **.002** | **.006** |
|  |  | Mean | 0.016 | 0.008 | 1.94 | .052 | .094 |
|  |  | Mean + 1 SD | 0.001 | 0.010 | 0.05 | .959 | .959 |
|  | Crystallized cognition | Mean - 1 SD | 0.040 | 0.010 | 3.88 | **<.001** | **<.001** |
|  |  | Mean | 0.016 | 0.008 | 1.95 | .051 | .094 |
|  |  | Mean + 1 SD | -0.008 | 0.010 | -0.76 | .446 | .502 |

Note. Conditional effects estimates indicate the strength of the association between age and global brain measures (i.e., change in brain measures over time) at different values of baseline general cognitive ability (mean - 1 SD, mean, and mean + 1 SD). Bold = significant at false-discovery rate corrected p-value (pFDR) < .05, corrected by imaging modality.

# Table S3. Cross-sectional differences in average gray matter structure for youth with higher vs lower baseline general cognitive ability

| **Time point** | ***Estimate*** | ***SE*** | ***t*** | ***p*** |
| --- | --- | --- | --- | --- |
| ***Surface area*** | | | | |
| **Baseline** | **0.1357** | **0.0164** | **8.25** | **<.001** |
| **2-year follow up** | **0.1304** | **0.0197** | **6.63** | **<.001** |
| **4-year follow up** | **0.1746** | **0.0223** | **7.83** | **<.001** |
| **6-year follow up** | **0.1338** | **0.0273** | **4.91** | **<.001** |
| ***Volume*** | | | | |
| **Baseline** | **0.1252** | **0.0160** | **7.81** | **<.001** |
| **2-year follow up** | **0.1193** | **0.0189** | **6.32** | **<.001** |
| **4-year follow up** | **0.1624** | **0.0212** | **7.66** | **<.001** |
| **6-year follow up** | **0.1185** | **0.0256** | **4.64** | **<.001** |

Note. Cross-sectional differences in gray matter structure for lower vs higher baseline general cognitive ability groups based on fluid cognition (NIH Toolbox). Participants were separated into lower and higher baseline groups using a median split. Bold indicates uncorrected p< 0.05.

# **Table S4. Non-linear models of whole brain measures of brain development and associations with baseline cognitive ability**

|  |  | | **Linear** | | | | | | | **Non-linear** | | | | | |  |
| --- | --- | --- | --- | --- | --- | --- | --- | --- | --- | --- | --- | --- | --- | --- | --- | --- |
| **Modality** | **MRI measure** | | ***β*** | ***SE*** | | ***df*** | ***t*** | ***p*** | | ***β*** | ***SE*** | ***df*** | ***t*** | ***p*** | |  |
| ***Matrix Reasoning*** | |  | | |  | | | |  |  | | | | |  | |
| rsfMRI | Between network FC | | **-0.0296** | **0.0061** | | **8430** | **-4.87** | **<.001** | | 0.0024 | 0.0057 | 16136 | 0.42 | 0.674 | |  |
|  | Within network FC | | -0.0039 | 0.0051 | | 8007 | -0.76 | 0.449 | | -0.0017 | 0.0048 | 15461 | -0.35 | 0.723 | |  |
| dMRI | Fractional anisotropy | | **-0.0067** | **0.0028** | | **8470** | **-2.37** | **0.018** | | 0.0005 | 0.0025 | 15807 | 0.20 | 0.843 | |  |
|  | Mean diffusivity | | 0.0006 | 0.0034 | | 8341 | 0.17 | 0.868 | | -0.0025 | 0.0029 | 15759 | -0.88 | 0.380 | |  |
| sMRI | Cortical thickness | | 0.0029 | 0.0027 | | 7713 | 1.09 | 0.277 | | -0.0026 | 0.0025 | 16472 | -1.04 | 0.300 | |  |
|  | Surface area | | 0.0014 | 0.001 | | 7413 | 1.45 | 0.148 | | -0.0003 | 0.0008 | 15982 | -0.41 | 0.683 | |  |
|  | Volume | | 0.0005 | 0.0012 | | 7678 | 0.40 | 0.687 | | **-0.0021** | **0.001** | **16030** | **-2.01** | **0.044** | |  |
| ***Fluid cognition*** | |  | | |  | | | |  |  | | | | |  | |
| rsfMRI | Between network FC | | **-0.0195** | **0.0061** | | **8473** | **-3.20** | **0.001** | | 0.0008 | 0.0057 | 16031 | 0.14 | 0.885 | |  |
|  | Within network FC | | 0.0027 | 0.0052 | | 8050 | 0.51 | 0.608 | | -0.0078 | 0.0048 | 15353 | -1.63 | 0.104 | |  |
| dMRI | Fractional anisotropy | | -0.0001 | 0.0028 | | 8420 | -0.03 | 0.974 | | -0.0027 | 0.0025 | 15595 | -1.08 | 0.281 | |  |
|  | Mean diffusivity | | 0.0041 | 0.0034 | | 8311 | 1.21 | 0.225 | | -0.0014 | 0.0029 | 15526 | -0.50 | 0.620 | |  |
| sMRI | Cortical thickness | | 0.0046 | 0.0027 | | 7701 | 1.69 | 0.092 | | -0.0007 | 0.0025 | 16282 | -0.29 | 0.770 | |  |
|  | Surface area | | 0.0002 | 0.001 | | 7416 | 0.18 | 0.856 | | 0.0002 | 0.0008 | 15713 | 0.25 | 0.801 | |  |
|  | Volume | | 0.0009 | 0.0012 | | 7624 | 0.75 | 0.453 | | -0.0004 | 0.001 | 15778 | -0.36 | 0.720 | |  |
| ***Crystallized cognition*** | |  | | |  | | | |  |  | | | | |  | |
| rsfMRI | Between network FC | | **-0.0259** | **0.0063** | | **8620** | **-4.14** | **<.001** | | -0.0008 | 0.0059 | 16021 | -0.14 | 0.888 | |  |
|  | Within network FC | | -0.0046 | 0.0053 | | 8211 | -0.87 | 0.386 | | -0.0036 | 0.0049 | 15355 | -0.73 | 0.466 | |  |
| dMRI | Fractional anisotropy | | 0.0027 | 0.0029 | | 8550 | 0.91 | 0.361 | | **-0.0055** | **0.0025** | **15504** | **-2.20** | **0.028** | |  |
|  | Mean diffusivity | | 0.0043 | 0.0034 | | 8407 | 1.25 | 0.210 | | -0.0047 | 0.0029 | 15386 | -1.60 | 0.109 | |  |
| sMRI | Cortical thickness | | 0.0048 | 0.0028 | | 8015 | 1.71 | 0.087 | | 0.0004 | 0.0025 | 16330 | 0.16 | 0.873 | |  |
|  | Surface area | | 0.0016 | 0.001 | | 7682 | 1.62 | 0.106 | | 0.0002 | 0.0008 | 15731 | 0.27 | 0.790 | |  |
|  | Volume | | -0.0004 | 0.0012 | | 7895 | -0.35 | 0.724 | | 0.0006 | 0.0011 | 15795 | 0.60 | 0.551 | |  |

Note. Statistics provided for age by baseline general cognitive ability (linear) and age^2^ by cognitive ability (non-linear) interactions in whole-brain models of brain development. General cognitive ability at baseline measured by matrix reasoning (Wechsler Intelligence Scale for Children), fluid cognition (NIH Toolbox) and crystallized cognition (NIH Toolbox), respectively. Bold indicates uncorrected p < .05. dMRI = diffusion MRI. rsfMRI = resting-state functional MRI. sMRI = structural MRI.

# Table S5. Interaction between age and baseline fluid cognition in models of gray matter structural development by region

| **Region** | **Surface area** | | | | | | **Volume** | | | | | |
| --- | --- | --- | --- | --- | --- | --- | --- | --- | --- | --- | --- | --- |
|  | ***β*** | ***SE*** | ***t*** | ***p*** | ***p_FDR_*** |  | ***β*** | ***SE*** | ***t*** | ***p*** | ***p_FDR_*** |  |
| Banks of superior temporal sulcus | -0.0018 | 0.0015 | -1.22 | .224 | .292 |  | -0.0024 | 0.0016 | -1.48 | .140 | .226 |  |
| Caudal anterior cingulate | -0.0026 | 0.0012 | -2.19 | .028 | .100 |  | -0.0045 | 0.0013 | -3.37 | .001 | .005 | * |
| Caudal middle frontal | -0.0031 | 0.0016 | -1.93 | .054 | .128 |  | -0.0043 | 0.0016 | -2.69 | .007 | .025 | * |
| Cuneus | 0.0031 | 0.0012 | 2.63 | .009 | .063 |  | 0.0049 | 0.0014 | 3.51 | .001 | .004 | * |
| Entorhinal | -0.0012 | 0.0022 | -0.57 | .566 | .621 |  | 0.0028 | 0.0025 | 1.14 | .256 | .378 |  |
| Fusiform | -0.0017 | 0.0011 | -1.58 | .114 | .193 |  | -0.001 | 0.0013 | -0.78 | .437 | .572 |  |
| Isthmus cingulate | -0.0024 | 0.0012 | -1.97 | .049 | .128 |  | -0.0009 | 0.0013 | -0.73 | .468 | .590 |  |
| Insula | -0.002 | 0.0023 | -0.86 | .388 | .440 |  | -0.0003 | 0.0021 | -0.17 | .869 | .923 |  |
| Inferior parietal | -0.0028 | 0.0013 | -2.22 | .027 | .100 |  | -0.0029 | 0.0013 | -2.21 | .027 | .083 |  |
| Inferior temporal | -0.0015 | 0.0012 | -1.28 | .201 | .274 |  | -0.0039 | 0.0013 | -3.05 | .002 | .011 | * |
| Lingual | 0.0003 | 0.001 | 0.26 | .795 | .795 |  | 0.0018 | 0.0012 | 1.59 | .113 | .201 |  |
| Lateral orbitofrontal | -0.0054 | 0.0021 | -2.54 | .011 | .063 |  | -0.0063 | 0.002 | -3.25 | .001 | .007 | * |
| Lateral occipital | 0.0004 | 0.001 | 0.43 | .668 | .697 |  | 0.0017 | 0.0012 | 1.40 | .163 | .251 |  |
| Medial orbitofrontal | -0.003 | 0.0023 | -1.31 | .189 | .268 |  | -0.0039 | 0.0023 | -1.70 | .089 | .175 |  |
| Middle temporal | -0.0021 | 0.0014 | -1.55 | .121 | .196 |  | -0.0047 | 0.0013 | -3.68 | .000 | .004 | * |
| Paracentral | -0.002 | 0.0014 | -1.40 | .163 | .252 |  | -0.0011 | 0.0019 | -0.59 | .559 | .678 |  |
| Pericalcarine | 0.0018 | 0.0011 | 1.62 | .106 | .190 |  | 0.0048 | 0.0014 | 3.49 | .001 | .004 | * |
| Posterior cingulate | -0.0018 | 0.001 | -1.72 | .086 | .171 |  | -0.001 | 0.0012 | -0.83 | .409 | .556 |  |
| Frontal pole | -0.0043 | 0.0025 | -1.73 | .083 | .171 |  | -0.0056 | 0.0028 | -2.04 | .042 | .109 |  |
| Parahippocampal | 0.0018 | 0.0019 | 0.97 | .334 | .392 |  | 0.0006 | 0.002 | 0.31 | .758 | .888 |  |
| Pars orbitalis | -0.0035 | 0.0014 | -2.48 | .013 | .065 |  | -0.0045 | 0.0016 | -2.76 | .006 | .022 | * |
| Postcentral | -0.0021 | 0.0015 | -1.35 | .177 | .262 |  | 0.0003 | 0.0015 | 0.20 | .844 | .923 |  |
| Pars opercularis | -0.0025 | 0.0015 | -1.65 | .099 | .186 |  | -0.0026 | 0.0017 | -1.56 | .118 | .201 |  |
| Precuneus | -0.0022 | 0.001 | -2.18 | .029 | .100 |  | 0.00003 | 0.0013 | 0.02 | .980 | .980 |  |
| Precentral | -0.0021 | 0.0017 | -1.20 | .232 | .292 |  | -0.0001 | 0.0017 | -0.07 | .941 | .969 |  |
| Pars triangularis | -0.0046 | 0.0014 | -3.18 | .002 | .025 | * | -0.0026 | 0.0016 | -1.68 | .092 | .175 |  |
| Temporal pole | -0.0047 | 0.0023 | -2.04 | .041 | .128 |  | -0.0005 | 0.003 | -0.16 | .869 | .923 |  |
| Rostral anterior cingulate | -0.0032 | 0.0016 | -1.99 | .046 | .128 |  | -0.0031 | 0.0018 | -1.74 | .081 | .175 |  |
| Rostral middle frontal | -0.0053 | 0.0016 | -3.33 | .001 | .025 | * | -0.006 | 0.0017 | -3.60 | .000 | .004 | * |
| Superior frontal | -0.0042 | 0.0014 | -2.90 | .004 | .042 | * | -0.0047 | 0.0016 | -2.95 | .003 | .013 | * |
| Supramarginal | -0.004 | 0.0015 | -2.59 | .010 | .063 |  | -0.0032 | 0.0015 | -2.12 | .034 | .096 |  |
| Superior parietal | -0.0016 | 0.0017 | -0.97 | .330 | .392 |  | 0.0031 | 0.0018 | 1.70 | .090 | .175 |  |
| Superior temporal | -0.0025 | 0.0013 | -1.91 | .057 | .128 |  | -0.0013 | 0.0013 | -1.02 | .310 | .439 |  |
| Transverse temporal | 0.0006 | 0.0014 | 0.42 | .677 | .697 |  | 0.0028 | 0.0015 | 1.83 | .068 | .165 |  |

Note. Statistics provided for age by baseline cognitive ability interactions in regional models of surface area and volume development. Fluid cognition indexed by NIH Toolbox. * Significant at false-discovery rate corrected p-value (p_FDR_)<.050 over 68 comparisons (34 regions each for area and volume).

# Table S6. Interaction between age and baseline fluid cognition in models of gray matter structural development by region, controlling for whole-brain total estimates

| **Region** | **Surface area** | | | | | **Volume** | | | | | | |
| --- | --- | --- | --- | --- | --- | --- | --- | --- | --- | --- | --- | --- |
|  | ***β*** | ***SE*** | ***t*** | ***p*** | ***p_FDR_*** |  | ***β*** | ***SE*** | ***t*** | ***p*** | ***p_FDR_*** |  |
| Banks of superior temporal sulcus | 0.0004 | 0.0012 | 0.30 | .763 | .851 |  | -0.0009 | 0.0014 | -0.66 | .511 | .695 |  |
| Caudal anterior cingulate | -0.0008 | 0.0011 | -0.70 | .481 | .695 |  | -0.0031 | 0.0012 | -2.52 | .012 | .067 |  |
| Caudal middle frontal | -0.0005 | 0.0013 | -0.38 | .705 | .841 |  | -0.0025 | 0.0013 | -1.88 | .060 | .178 |  |
| Cuneus | 0.0047 | 0.0011 | 4.17 | <.001 | .001 | * | 0.0062 | 0.0012 | 5.01 | <.001 | <.001 | * |
| Entorhinal | -0.0001 | 0.0021 | -0.05 | .961 | .972 |  | 0.0038 | 0.0025 | 1.56 | .120 | .313 |  |
| Fusiform | 0.0007 | 0.0009 | 0.75 | .455 | .695 |  | 0.0008 | 0.0011 | 0.73 | .468 | .695 |  |
| Isthmus cingulate | -0.0008 | 0.0012 | -0.66 | .507 | .695 |  | 0.0005 | 0.0012 | 0.41 | .685 | .841 |  |
| Insula | 0.0001 | 0.0023 | 0.06 | .951 | .972 |  | 0.0015 | 0.002 | 0.77 | .439 | .695 |  |
| Inferior parietal | -0.0003 | 0.0009 | -0.32 | .751 | .851 |  | -0.0012 | 0.0009 | -1.31 | .189 | .458 |  |
| Inferior temporal | 0.0011 | 0.0009 | 1.23 | .221 | .496 |  | -0.0019 | 0.0009 | -1.99 | .047 | .163 |  |
| Lingual | 0.0019 | 0.0009 | 1.97 | .049 | .163 |  | 0.0032 | 0.001 | 3.24 | .001 | .012 | * |
| Lateral orbitofrontal | -0.0025 | 0.002 | -1.25 | .213 | .496 |  | -0.0039 | 0.0016 | -2.46 | .014 | .073 |  |
| Lateral occipital | 0.0025 | 0.0008 | 3.15 | .002 | .013 | * | 0.0035 | 0.0009 | 3.75 | <.001 | .003 | * |
| Medial orbitofrontal | -0.0002 | 0.0022 | -0.10 | .918 | .960 |  | -0.0018 | 0.002 | -0.91 | .363 | .649 |  |
| Middle temporal | 0.0006 | 0.0009 | 0.64 | .524 | .699 |  | -0.0026 | 0.0008 | -3.13 | .002 | .013 | * |
| Paracentral | 0.0003 | 0.0013 | 0.24 | .810 | .888 |  | 0.0011 | 0.0015 | 0.75 | .454 | .695 |  |
| Pericalcarine | 0.0031 | 0.0011 | 2.79 | .005 | .033 | * | 0.0059 | 0.0013 | 4.50 | <.001 | <.001 | * |
| Posterior cingulate | -0.00003 | 0.001 | -0.03 | .972 | .972 |  | 0.0006 | 0.001 | 0.67 | .500 | .695 |  |
| Frontal pole | -0.0018 | 0.0024 | -0.76 | .445 | .695 |  | -0.0041 | 0.0027 | -1.53 | .125 | .315 |  |
| Parahippocampal | 0.0035 | 0.0018 | 1.94 | .053 | .163 |  | 0.0018 | 0.0019 | 0.96 | .336 | .618 |  |
| Pars orbitalis | -0.0012 | 0.0012 | -0.96 | .336 | .618 |  | -0.0025 | 0.0013 | -1.95 | .051 | .163 |  |
| Postcentral | 0.0007 | 0.0013 | 0.53 | .595 | .750 |  | 0.0023 | 0.0011 | 2.07 | .038 | .163 |  |
| Pars opercularis | -0.0003 | 0.0013 | -0.21 | .837 | .903 |  | -0.0008 | 0.0014 | -0.59 | .553 | .723 |  |
| Precuneus | -0.0001 | 0.0009 | -0.11 | .909 | .960 |  | 0.002 | 0.001 | 2.04 | .041 | .163 |  |
| Precentral | 0.0005 | 0.0014 | 0.39 | .696 | .841 |  | 0.0022 | 0.0012 | 1.76 | .078 | .211 |  |
| Pars triangularis | -0.0024 | 0.0012 | -1.98 | .047 | .163 |  | -0.0011 | 0.0013 | -0.83 | .408 | .695 |  |
| Temporal pole | -0.0027 | 0.0022 | -1.20 | .230 | .496 |  | 0.001 | 0.0029 | 0.33 | .742 | .851 |  |
| Rostral anterior cingulate | -0.0009 | 0.0016 | -0.56 | .574 | .736 |  | -0.0013 | 0.0017 | -0.78 | .435 | .695 |  |
| Rostral middle frontal | -0.0025 | 0.0013 | -1.95 | .051 | .163 |  | -0.0037 | 0.0013 | -2.94 | .003 | .022 | * |
| Superior frontal | -0.0012 | 0.0011 | -1.12 | .263 | .525 |  | -0.0023 | 0.0011 | -2.11 | .035 | .163 |  |
| Supramarginal | -0.0015 | 0.0012 | -1.19 | .234 | .496 |  | -0.0014 | 0.0012 | -1.17 | .243 | .501 |  |
| Superior parietal | 0.001 | 0.0014 | 0.72 | .471 | .695 |  | 0.005 | 0.0014 | 3.51 | <.001 | .006 | * |
| Superior temporal | 0.0003 | 0.0009 | 0.34 | .733 | .851 |  | 0.0009 | 0.0008 | 1.02 | .305 | .593 |  |
| Transverse temporal | 0.0024 | 0.0013 | 1.84 | .065 | .185 |  | 0.0045 | 0.0013 | 3.37 | .001 | .009 | * |

Note. Statistics provided for age by baseline general cognitive ability interactions in regional models of surface area and volume development, after controlling for whole brain average surface area and volume, respectively. Fluid cognition indexed by NIH Toolbox. * Significant at false-discovery rate corrected p-value (*p_FDR_*) < .050 over 68 comparisons (34 regions each for area and volume).

# Table S7. Interaction between age and baseline fluid cognition in global models of gray matter structural development, controlling for Euler number–derived topology defects

| **MRI measure** | **Cognitive measure** | **Variable** | ***β*** | ***SE*** | ***df*** | ***t*** | ***p*** |
| --- | --- | --- | --- | --- | --- | --- | --- |
| Cortical surface area | Fluid cognition | Intercept | 0.3757 | 0.0282 | 26 | 13.30 | <.001 |
|  |  | Age | -0.0363 | 0.0013 | 6945 | -27.57 | <.001 |
|  |  | Cognitive ability | 0.0776 | 0.0082 | 9665 | 9.45 | <.001 |
|  |  | Sex (Female vs Male) | -0.9758 | 0.0160 | 9959 | -60.94 | <.001 |
|  |  | Baseline INR | 0.1721 | 0.0093 | 8236 | 18.52 | <.001 |
|  |  | **Age x Cognitive ability** | -0.0035 | 0.0010 | 6285 | -3.70 | <.001 |
|  |  | Age x Sex | -0.0582 | 0.0018 | 6345 | -31.73 | <.001 |
|  |  | Age x Baseline INR | 0.0038 | 0.0010 | 6425 | 3.93 | <.001 |
|  |  | Achieva dStream* | -0.2322 | 0.0341 | 1059 | -6.82 | <.001 |
|  |  | DISCOVERY MR750* | 0.3807 | 0.0185 | 2534 | 20.57 | <.001 |
|  |  | Ingenia* | -0.2318 | 0.0442 | 1344 | -5.24 | <.001 |
|  |  | MAGNETOM Prisma* | -0.0671 | 0.0254 | 12217 | -2.64 | .008 |
|  |  | MAGNETOM Prisma Fit* | -0.0733 | 0.0100 | 12558 | -7.33 | <.001 |
|  |  | Orchestra SDK | 0.5088 | 0.1491 | 14966 | 3.41 | .001 |
|  |  | Prisma* | 0.0294 | 0.0058 | 14428 | 5.07 | <.001 |
|  |  | SIGNA Premier* | 0.3367 | 0.0206 | 3501 | 16.37 | <.001 |
|  |  | SIGNA UHP* | 0.3111 | 0.0204 | 3483 | 15.22 | <.001 |
|  |  | Number of topology defects  (from Euler number) | 0.0490 | 0.0012 | 15997 | 40.85 | <.001 |
| Cortical volume | Fluid cognition | Intercept | 0.3676 | 0.0338 | 25 | 10.87 | <.001 |
|  |  | Age | -0.2463 | 0.0017 | 7312 | -147.85 | <.001 |
|  |  | Cognitive ability | 0.0662 | 0.0080 | 9499 | 8.29 | <.001 |
|  |  | Sex (Female vs Male) | -0.9375 | 0.0156 | 9881 | -60.16 | <.001 |
|  |  | Baseline INR | 0.2033 | 0.0091 | 8555 | 22.27 | <.001 |
|  |  | **Age x Cognitive ability** | -0.0024 | 0.0012 | 6637 | -2.00 | .046 |
|  |  | Age x Sex | -0.0928 | 0.0023 | 6702 | -39.94 | <.001 |
|  |  | Age x Baseline INR | 0.0045 | 0.0012 | 6784 | 3.77 | <.001 |
|  |  | Achieva dStream* | -0.1768 | 0.0420 | 1029 | -4.21 | <.001 |
|  |  | DISCOVERY MR750* | 0.1754 | 0.0229 | 2499 | 7.64 | <.001 |
|  |  | Ingenia* | -0.1398 | 0.0547 | 1268 | -2.56 | .011 |
|  |  | MAGNETOM Prisma* | -0.0384 | 0.0318 | 12930 | -1.21 | .227 |
|  |  | MAGNETOM Prisma Fit* | -0.0421 | 0.0125 | 13285 | -3.37 | .001 |
|  |  | Orchestra SDK | 0.0475 | 0.1872 | 15391 | 0.25 | .800 |
|  |  | Prisma* | 0.0417 | 0.0073 | 14828 | 5.74 | <.001 |
|  |  | SIGNA Premier* | 0.2200 | 0.0255 | 3327 | 8.62 | <.001 |
|  |  | SIGNA UHP* | 0.1258 | 0.0254 | 3373 | 4.96 | <.001 |
|  |  | Number of topology defects  (from Euler number) | -0.0420 | 0.0015 | 16172 | -28.18 | <.001 |

Note. Statistics provided for global models of surface area and volume development, after controlling for the number of topology defects calculated from the Euler number from FreeSurfer. Bold indicates age x cognitive ability interaction of primary interest, where general cognitive ability at baseline was indexed by NIH Toolbox Fluid cognition scores. * Estimates are shown for each MRI model type compared to the Prisma Fit model as the reference group. INR = income-to-needs ratio.

# Table S8. Interaction between age and baseline fluid cognition in global models of gray matter structural development by region, controlling for Euler number–derived topology defects

| **Region** | ***β*** | ***SE*** | ***t*** | ***p*** | ***p_FDR_*** |  |  | |  |
| --- | --- | --- | --- | --- | --- | --- | --- | --- | --- |
| ***Surface area*** |  | | | | |  |  |  |  |
| Pars triangularis | -0.0048 | 0.0014 | -3.33 | .001 | .015 | * |  |  |  |
| Rostral middle frontal | -0.0055 | 0.0016 | -3.43 | .001 | .015 | * |  |  |  |
| Superior frontal | -0.0044 | 0.0014 | -3.16 | .002 | .018 | * |  |  |  |
| Supramarginal | -0.0042 | 0.0015 | -2.89 | .004 | .033 | * |  |  |  |
| ***Volume*** |  | | | | |  |  | | |
| Caudal anterior cingulate | -0.0044 | 0.0013 | -3.32 | .001 | .006 | * |  |  |  |
| Caudal middle frontal | -0.0043 | 0.0016 | -2.67 | .008 | .026 | * |  |  |  |
| Cuneus | 0.0050 | 0.0014 | 3.66 | <.001 | .003 | * |  |  |  |
| Inferior temporal | -0.0037 | 0.0012 | -2.94 | .003 | .014 | * |  |  |  |
| Lateral orbitofrontal | -0.0060 | 0.0019 | -3.17 | .002 | .009 | * |  |  |  |
| Middle temporal | -0.0045 | 0.0012 | -3.62 | <.001 | .003 | * |  |  |  |
| Pericalcarine | 0.0050 | 0.0014 | 3.67 | <.001 | .003 | * |  |  |  |
| Pars orbitalis | -0.0044 | 0.0016 | -2.72 | .007 | .025 | * |  |  |  |
| Rostral middle frontal | -0.0059 | 0.0017 | -3.54 | <.001 | .003 | * |  |  |  |
| Superior frontal | -0.0047 | 0.0016 | -2.95 | .003 | .014 | * |  |  |  |

Note. Statistics provided for age by baseline general cognitive ability interactions in regional models of surface area and volume development, after controlling for the number of topology defects calculated from the Euler number from FreeSurfer. Fluid cognition indexed by NIH Toolbox. * Significant at false-discovery rate corrected p-value (*p_FDR_*) < .050 over 68 comparisons (34 regions each for area and volume).

# Table S9. Interaction between age and baseline matrix reasoning scaled score in whole brain measures of brain white matter development

| **MRI measure** | **Cognitive measure** | **Variable** | ***β*** | ***SE*** | ***df*** | ***t*** | ***p*** |
| --- | --- | --- | --- | --- | --- | --- | --- |
| Fractional anisotropy | Matrix reasoning | Intercept | 0.2198 | 0.0456 | 27 | 4.82 | <.001 |
|  |  | Age | 0.1716 | 0.0038 | 8133 | 45.09 | <.001 |
|  |  | Cognitive ability | 0.0176 | 0.0052 | 9166 | 3.37 | .001 |
|  |  | Sex (Female vs Male) | 0.0349 | 0.0101 | 9803 | 3.44 | .001 |
|  |  | Baseline INR | 0.0201 | 0.0059 | 8604 | 3.42 | .001 |
|  |  | Mean FD | -0.2534 | 0.0037 | 19756 | -67.64 | <.001 |
|  |  | **Age x Cognitive ability** | **-0.0065** | **0.0028** | **7565** | **-2.33** | **.020** |
|  |  | Age x Sex | 0.0083 | 0.0053 | 7435 | 1.57 | .117 |
|  |  | Age x Baseline INR | 0.0109 | 0.0028 | 7609 | 3.96 | <.001 |
|  |  | Achieva dStream* | -1.8654 | 0.0775 | 280 | -24.07 | <.001 |
|  |  | DISCOVERY MR750* | -0.3832 | 0.0486 | 430 | -7.89 | <.001 |
|  |  | Ingenia* | -2.4591 | 0.1171 | 184 | -21.00 | <.001 |
|  |  | MAGNETOM Prisma* | 0.1199 | 0.0766 | 11888 | 1.57 | .118 |
|  |  | MAGNETOM Prisma Fit* | -0.0770 | 0.0284 | 12393 | -2.71 | .007 |
|  |  | Prisma* | 0.1886 | 0.0160 | 14288 | 11.79 | <.001 |
|  |  | SIGNA Premier* | -0.3815 | 0.0551 | 677 | -6.92 | <.001 |
|  |  | SIGNA UHP* | -0.0407 | 0.0541 | 648 | -0.75 | .452 |

Note. Bold indicates age x cognitive ability interaction of primary interest, where cognitive ability at baseline was indexed by Wechsler Intelligence Scale for Children - Matrix Reasoning score. ^ The age x cognitive ability x sex interaction was not significant for the fractional anisotropy model. * Estimates are shown for each MRI model type compared to the Prisma Fit model as the reference group. INR = income-to-needs ratio. Mean FD = mean framewise displacement.

# Table S10. Cross-sectional differences in average fractional anisotropy for youth with higher vs lower baseline general cognitive ability

| **Time point** | ***Estimate*** | ***SE*** | ***t*** | ***p*** |
| --- | --- | --- | --- | --- |
| **Baseline** | **0.0443** | **0.0113** | **3.916** | **<.001** |
| **2-year follow up** | **0.0267** | **0.0127** | **2.102** | **.036** |
| **4-year follow up** | **0.0337** | **0.0141** | **2.393** | **.017** |
| 6-year follow up | 0.0253 | 0.0172 | 1.47 | .142 |

Note. Cross-sectional differences in between-network resting-state functional connectivity for lower vs higher baseline general cognitive ability groups indexed by matrix reasoning (Wechsler Intelligence Scale for Children). Participants were separated into lower and higher baseline groups using a median split. Bold indicates uncorrected p< 0.05.

# Table S11. Interaction between age and baseline matrix reasoning in models of fractional anisotropy development by bilateral white matter tract

| **White matter tract** | ***β*** | ***SE*** | ***t*** | ***p*** | ***p_FDR_*** |  |
| --- | --- | --- | --- | --- | --- | --- |
| Anterior thalamic radiations | -0.0021 | 0.0039 | -0.54 | .591 | .682 |  |
| Cingulum (cingulate) | -0.0036 | 0.0031 | -1.16 | .246 | .369 |  |
| Cingulum (parahippocampal) | -0.0002 | 0.003 | -0.07 | .941 | .941 |  |
| Corticospinal tract or pyramidal tract | -0.0103 | 0.0033 | -3.15 | .002 | .010 | * |
| Fornix | -0.0057 | 0.0035 | -1.63 | .103 | .222 |  |
| Inferior fronto-occipital fasciculus | -0.0004 | 0.0026 | -0.15 | .883 | .941 |  |
| Inferior frontal superior frontal cortex | -0.0076 | 0.0029 | -2.57 | .010 | .032 | * |
| Inferior longitudinal fasciculus | -0.0036 | 0.0028 | -1.29 | .199 | .331 |  |
| Superior corticostriate | -0.0108 | 0.0032 | -3.34 | .001 | .010 | * |
| Striatal inferior frontal cortex | 0.0016 | 0.0027 | 0.61 | .539 | .674 |  |
| Superior longitudinal fasciculus | -0.0088 | 0.0028 | -3.10 | .002 | .010 | * |
| Uncinate fasciculus | -0.0022 | 0.0029 | -0.76 | .445 | .606 |  |
| Corpus callosum | -0.0067 | 0.0026 | -2.55 | .011 | .032 | * |
| Forceps major | -0.0055 | 0.0025 | -2.22 | .026 | .066 |  |
| Forceps minor | -0.0038 | 0.0029 | -1.33 | .185 | .331 |  |

Note. Statistics provided for age by baseline general cognitive ability interactions in tract-wise models of fractional anisotropy development. Matrix reasoning indexed by Wechsler Intelligence Scale for Children. * Significant at false-discovery rate corrected p-value (*p_FDR_*) < .05 over 15 white matter tracts.

# Table S12. Interaction between age and baseline general cognitive ability in whole brain measures of brain functional connectivity development

| **MRI measure** | **Cognitive measure** | **Variable** | ***β*** | ***SE*** | ***df*** | ***t*** | ***p*** |
| --- | --- | --- | --- | --- | --- | --- | --- |
| Between-network resting-state functional connectivity | Matrix reasoning | Intercept | 0.2983 | 0.0539 | 29 | 5.54 | <.001 |
|  |  | Age | 0.0174 | 0.0083 | 7861 | 2.09 | .037 |
|  |  | Cognitive ability | -0.0038 | 0.008 | 8961 | -0.47 | .640 |
|  |  | Sex (Female vs Male) | -0.2919 | 0.0153 | 8928 | -19.05 | <.001 |
|  |  | Baseline INR | -0.0152 | 0.0086 | 7871 | -1.76 | .078 |
|  |  | Mean FD | 0.3336 | 0.0113 | 19208 | 29.39 | <.001 |
|  |  | **Age x Cognitive ability** | **-0.0283** | **0.006** | **6934** | **-4.75** | **<.001** |
|  |  | Age x Sex | 0.0001 | 0.0113 | 6803 | 0.01 | .996 |
|  |  | Age x Baseline INR | -0.0313 | 0.0059 | 6990 | -5.32 | <.001 |
|  |  | Achieva dStream* | -0.1243 | 0.122 | 61 | -1.02 | .313 |
|  |  | DISCOVERY MR750* | -0.2308 | 0.0772 | 89 | -2.99 | .004 |
|  |  | Ingenia* | 0.3457 | 0.1788 | 43 | 1.93 | .060 |
|  |  | MAGNETOM Prisma* | 0.075 | 0.163 | 9791 | 0.46 | .645 |
|  |  | MAGNETOM Prisma Fit* | 0.0767 | 0.062 | 10459 | 1.24 | .216 |
|  |  | Prisma* | -0.0378 | 0.0333 | 3552 | -1.13 | .257 |
|  |  | SIGNA Premier* | -0.1996 | 0.0964 | 201 | -2.07 | .040 |
|  |  | SIGNA UHP* | -0.0677 | 0.0928 | 184 | -0.73 | .467 |
| Between-network resting-state functional connectivity | Fluid cognition | Intercept | 0.2884 | 0.0535 | 29 | 5.39 | <.001 |
|  |  | Age | 0.0187 | 0.0084 | 7723 | 2.24 | .025 |
|  |  | Cognitive ability | -0.0324 | 0.008 | 9029 | -4.03 | <.001 |
|  |  | Sex (Female vs Male) | -0.2821 | 0.0154 | 8842 | -18.29 | <.001 |
|  |  | Baseline INR | -0.0107 | 0.0086 | 7702 | -1.24 | .214 |
|  |  | Mean FD | 0.3282 | 0.0114 | 19068 | 28.72 | <.001 |
|  |  | **Age x Cognitive ability** | **-0.0161** | **0.0059** | **6764** | **-2.72** | **.007** |
|  |  | Age x Sex | -0.0015 | 0.0113 | 6720 | -0.14 | .893 |
|  |  | Age x Baseline INR | -0.0345 | 0.0059 | 6852 | -5.86 | <.001 |
|  |  | Achieva dStream* | -0.101 | 0.1226 | 58 | -0.82 | .414 |
|  |  | DISCOVERY MR750* | -0.204 | 0.0764 | 90 | -2.67 | .009 |
|  |  | Ingenia* | 0.4248 | 0.1701 | 52 | 2.50 | .016 |
|  |  | MAGNETOM Prisma* | 0.0437 | 0.1581 | 9558 | 0.28 | .782 |
|  |  | MAGNETOM Prisma Fit* | 0.0679 | 0.0626 | 10335 | 1.08 | .279 |
|  |  | Prisma* | -0.0456 | 0.0335 | 3367 | -1.36 | .174 |
|  |  | SIGNA Premier* | -0.1586 | 0.0952 | 202 | -1.67 | .097 |
|  |  | SIGNA UHP* | -0.0376 | 0.0923 | 191 | -0.41 | .684 |
| Between-network resting-state functional connectivity | Crystallized cognition | Intercept | 0.2909 | 0.0539 | 29 | 5.40 | <.001 |
|  |  | Age | 0.0193 | 0.0084 | 7743 | 2.31 | .021 |
|  |  | Cognitive ability | -0.0018 | 0.0083 | 8780 | -0.22 | .830 |
|  |  | Sex (Female vs Male) | -0.2837 | 0.0154 | 8866 | -18.43 | .000 |
|  |  | Baseline INR | -0.0166 | 0.0089 | 7818 | -1.87 | .061 |
|  |  | Mean FD | 0.3302 | 0.0114 | 19090 | 29.05 | <.001 |
|  |  | **Age x Cognitive ability** | **-0.0242** | **0.0061** | **6740** | **-3.98** | **<.001** |
|  |  | Age x Sex | -0.0034 | 0.0113 | 6738 | -0.30 | .760 |
|  |  | Age x Baseline INR | -0.0297 | 0.0061 | 6857 | -4.88 | <.001 |
|  |  | Achieva dStream* | -0.0954 | 0.1232 | 58 | -0.77 | .442 |
|  |  | DISCOVERY MR750* | -0.2134 | 0.0767 | 91 | -2.78 | .007 |
|  |  | Ingenia* | 0.4157 | 0.171 | 52 | 2.43 | .018 |
|  |  | MAGNETOM Prisma* | 0.0521 | 0.1581 | 9573 | 0.33 | .742 |
|  |  | MAGNETOM Prisma Fit* | 0.0763 | 0.0626 | 10353 | 1.22 | .223 |
|  |  | Prisma* | -0.0493 | 0.0334 | 3506 | -1.48 | .140 |
|  |  | SIGNA Premier* | -0.1628 | 0.0955 | 204 | -1.70 | .090 |
|  |  | SIGNA UHP* | -0.0462 | 0.0924 | 192 | -0.50 | .618 |

Note. Bold indicates age x cognitive ability interaction of primary interest, where cognitive ability at baseline was indexed by Wechsler Intelligence Scale for Children - Matrix Reasoning, NIH Toolbox Fluid scores, and NIH Toolbox Crystallized scores. * Estimates are shown for each MRI model type compared to the Prisma Fit model as the reference group. INR = income-to-needs ratio. Mean FD = mean framewise displacement.

# Table S13. Cross-sectional differences in average between-network resting-state functional connectivity for youth with higher vs lower baseline general cognitive ability

| **Time point** | ***Estimate*** | ***SE*** | ***t*** | ***p*** |
| --- | --- | --- | --- | --- |
| ***Matrix Reasoning*** | | | | |
| **Baseline** | **0.0628** | **0.0210** | **2.99** | **.003** |
| 2-year follow up | 0.0060 | 0.0235 | 0.26 | .799 |
| 4-year follow up | -0.0364 | 0.0251 | -1.45 | .148 |
| 6-year follow up | -0.0568 | 0.0327 | -1.74 | .082 |
| ***Fluid Cognition*** | | | | |
| **Baseline** | **-0.0428** | **0.0213** | **-2.01** | **.045** |
| **2-year follow up** | **-0.0649** | **0.0237** | **-2.74** | **.006** |
| **4-year follow up** | -0.0499 | 0.0256 | -1.95 | .051 |
| **6-year follow up** | **-0.1125** | **0.0330** | **-3.41** | **.001** |
| ***Crystallized Cognition*** | | | | |
| Baseline | 0.0366 | 0.0220 | 1.67 | .096 |
| 2-year follow up | 0.0022 | 0.0244 | 0.09 | .927 |
| 4-year follow up | -0.0289 | 0.0262 | -1.10 | .271 |
| **6-year follow up** | **-0.1007** | **0.0338** | **-2.98** | **.003** |

Note. Cross-sectional differences in between-network resting-state functional connectivity for lower vs higher baseline general cognitive ability groups. General cognitive ability at baseline measured by matrix reasoning (Wechsler Intelligence Scale for Children), fluid cognition (NIH Toolbox) and crystallized cognition (NIH Toolbox), respectively. Participants were separated into lower and higher baseline cognition groups using a median split for each cognitive measure. Bold indicates uncorrected p < 0.05.

# Table S14. Interaction between age and baseline general cognitive ability in models of between-network resting-state functional connectivity development by network pair

| **Network pair** | **WISC Matrix** | | | | | | **Fluid** | | | | | | **Crystallized** | | | | | |  |
| --- | --- | --- | --- | --- | --- | --- | --- | --- | --- | --- | --- | --- | --- | --- | --- | --- | --- | --- | --- |
|  | ***β*** | ***SE*** | ***t*** | ***p*** | ***p_FDR_*** |  | ***β*** | ***SE*** | ***t*** | ***p*** | ***p_FDR_*** |  | ***β*** | ***SE*** | ***t*** | ***p*** | ***p_FDR_*** |  |  |
| AN-CON | -0.0255 | 0.0064 | -4.01 | .000 | .001 | * | -0.0293 | 0.0063 | -4.64 | .000 | .000 | * | -0.0280 | 0.0065 | -4.34 | .000 | .000 | * |  |
| AN-CPN | -0.0199 | 0.0063 | -3.15 | .002 | .016 | * | -0.0134 | 0.0063 | -2.12 | .034 | .139 |  | -0.0022 | 0.0065 | -0.33 | .739 | .801 |  |  |
| AN-DMN | -0.0126 | 0.0061 | -2.08 | .038 | .140 |  | -0.0071 | 0.0060 | -1.18 | .239 | .445 |  | -0.0110 | 0.0062 | -1.79 | .074 | .181 |  |  |
| AN-DAN | 0.0014 | 0.0065 | 0.21 | .835 | .879 |  | -0.0104 | 0.0065 | -1.61 | .107 | .269 |  | 0.0137 | 0.0066 | 2.06 | .039 | .136 |  |  |
| AN-FPN | -0.0101 | 0.0059 | -1.71 | .087 | .224 |  | -0.0159 | 0.0059 | -2.68 | .007 | .043 | * | -0.0107 | 0.0061 | -1.77 | .077 | .182 |  |  |
| AN-RTN | 0.0017 | 0.0061 | 0.28 | .776 | .853 |  | 0.0208 | 0.0061 | 3.41 | .001 | .008 | * | 0.0077 | 0.0062 | 1.23 | .218 | .360 |  |  |
| AN-SN | -0.0262 | 0.0062 | -4.22 | .000 | .001 | * | -0.0264 | 0.0062 | -4.27 | .000 | .001 | * | -0.0310 | 0.0063 | -4.89 | .000 | .000 | * |  |
| AN-SMN (H) | -0.0021 | 0.0061 | -0.35 | .727 | .846 |  | 0.0054 | 0.0060 | 0.89 | .374 | .573 |  | -0.0079 | 0.0062 | -1.28 | .202 | .350 |  |  |
| AN-SMN (M) | 0.0024 | 0.0062 | 0.38 | .703 | .844 |  | 0.0043 | 0.0062 | 0.69 | .491 | .672 |  | -0.0069 | 0.0063 | -1.10 | .273 | .409 |  |  |
| AN-VAN | -0.0132 | 0.0060 | -2.21 | .027 | .121 |  | -0.0010 | 0.0059 | -0.16 | .872 | .941 |  | -0.0158 | 0.0061 | -2.60 | .009 | .038 | * |  |
| AN-VN | 0.0086 | 0.0062 | 1.39 | .166 | .332 |  | 0.0196 | 0.0061 | 3.20 | .001 | .012 | * | 0.0123 | 0.0063 | 1.96 | .050 | .143 |  |  |
| CON-CPN | 0.0066 | 0.0058 | 1.13 | .257 | .466 |  | 0.0089 | 0.0058 | 1.54 | .124 | .296 |  | 0.0014 | 0.0059 | 0.23 | .818 | .840 |  |  |
| CON-DMN | -0.0204 | 0.0058 | -3.54 | .000 | .005 | * | -0.0120 | 0.0057 | -2.08 | .037 | .146 |  | -0.0256 | 0.0059 | -4.35 | .000 | .000 | * |  |
| CON-DAN | 0.0165 | 0.0059 | 2.79 | .005 | .039 | * | -0.0001 | 0.0059 | -0.02 | .987 | .993 |  | 0.0180 | 0.0060 | 2.98 | .003 | .017 | * |  |
| CON-FPN | -0.0018 | 0.0058 | -0.31 | .755 | .853 |  | -0.0047 | 0.0058 | -0.80 | .424 | .612 |  | -0.0032 | 0.0059 | -0.54 | .590 | .677 |  |  |
| CON-RTN | -0.0019 | 0.0060 | -0.32 | .748 | .853 |  | 0.0011 | 0.0059 | 0.19 | .848 | .941 |  | -0.0115 | 0.0061 | -1.89 | .059 | .153 |  |  |
| CON-SN | -0.0132 | 0.0060 | -2.20 | .028 | .121 |  | -0.0101 | 0.0060 | -1.69 | .092 | .255 |  | -0.0266 | 0.0061 | -4.36 | .000 | .000 | * |  |
| CON-SMN (H) | -0.0206 | 0.0062 | -3.35 | .001 | .009 | * | -0.0208 | 0.0061 | -3.39 | .001 | .008 | * | -0.0165 | 0.0063 | -2.63 | .009 | .038 | * |  |
| CON-SMN (M) | -0.0181 | 0.0065 | -2.78 | .005 | .039 | * | -0.0031 | 0.0065 | -0.47 | .636 | .788 |  | -0.0047 | 0.0066 | -0.71 | .479 | .599 |  |  |
| CON-VAN | -0.0101 | 0.0056 | -1.82 | .070 | .201 |  | -0.0061 | 0.0056 | -1.09 | .274 | .464 |  | -0.0170 | 0.0057 | -3.00 | .003 | .017 | * |  |
| CON-VN | 0.0062 | 0.0064 | 0.97 | .331 | .548 |  | 0.0163 | 0.0064 | 2.55 | .011 | .050 | * | 0.0171 | 0.0065 | 2.62 | .009 | .038 | * |  |
| CPN-DMN | -0.0010 | 0.0055 | -0.19 | .853 | .879 |  | 0.0017 | 0.0054 | 0.31 | .755 | .893 |  | -0.0059 | 0.0056 | -1.06 | .289 | .419 |  |  |
| **Network pair** | **WISC Matrix** | | | | | | **Fluid** | | | | | | **Crystallized** | | | | | | |
|  | ***β*** | ***SE*** | ***t*** | ***p*** | ***p_FDR_*** |  | ***β*** | ***SE*** | ***t*** | ***p*** | ***p_FDR_*** |  | ***β*** | ***SE*** | ***t*** | ***p*** | ***p_FDR_*** |  |  |
| CPN-DAN | 0.0066 | 0.0057 | 1.15 | .251 | .466 |  | -0.0014 | 0.0057 | -0.24 | .809 | .941 |  | 0.0012 | 0.0058 | 0.20 | .841 | .852 |  |  |
| CPN-FPN | -0.0035 | 0.0058 | -0.59 | .553 | .751 |  | -0.0086 | 0.0058 | -1.49 | .138 | .298 |  | -0.0089 | 0.0059 | -1.51 | .131 | .268 |  |  |
| CPN-RTN | -0.0095 | 0.0055 | -1.72 | .086 | .224 |  | -0.0181 | 0.0055 | -3.28 | .001 | .010 | * | 0.0015 | 0.0056 | 0.27 | .789 | .832 |  |  |
| CPN-SN | 0.0011 | 0.0057 | 0.20 | .842 | .879 |  | 0.0103 | 0.0056 | 1.83 | .067 | .202 |  | 0.0015 | 0.0058 | 0.27 | .789 | .832 |  |  |
| CPN-SMN (H) | -0.0227 | 0.0064 | -3.54 | .000 | .005 | * | -0.0160 | 0.0064 | -2.49 | .013 | .055 |  | -0.0065 | 0.0066 | -0.99 | .324 | .443 |  |  |
| CPN-SMN (M) | -0.0195 | 0.0065 | -3.00 | .003 | .024 | * | -0.0200 | 0.0065 | -3.08 | .002 | .016 | * | -0.0127 | 0.0066 | -1.91 | .056 | .150 |  |  |
| CPN-VAN | -0.0035 | 0.0062 | -0.57 | .568 | .751 |  | 0.0007 | 0.0061 | 0.11 | .910 | .941 |  | -0.0040 | 0.0063 | -0.64 | .522 | .612 |  |  |
| CPN-VN | 0.0090 | 0.0055 | 1.64 | .101 | .238 |  | 0.0025 | 0.0054 | 0.45 | .652 | .794 |  | 0.0088 | 0.0056 | 1.58 | .115 | .249 |  |  |
| DMN-DAN | -0.0145 | 0.0055 | -2.62 | .009 | .049 | * | -0.0060 | 0.0055 | -1.08 | .279 | .464 |  | -0.0222 | 0.0056 | -3.94 | .000 | .001 | * |  |
| DMN-FPN | -0.0155 | 0.0058 | -2.69 | .007 | .047 | * | 0.0006 | 0.0058 | 0.10 | .917 | .941 |  | -0.0199 | 0.0059 | -3.39 | .001 | .006 | * |  |
| DMN-RTN | 0.0041 | 0.0058 | 0.70 | .483 | .712 |  | 0.0095 | 0.0058 | 1.64 | .102 | .269 |  | 0.0234 | 0.0059 | 3.97 | .000 | .001 | * |  |
| DMN-SN | -0.0105 | 0.0057 | -1.84 | .066 | .201 |  | -0.0011 | 0.0056 | -0.19 | .850 | .941 |  | -0.0058 | 0.0058 | -1.01 | .313 | .443 |  |  |
| DMN-SMN (H) | -0.0072 | 0.0059 | -1.20 | .229 | .446 |  | -0.0048 | 0.0059 | -0.81 | .417 | .612 |  | -0.0028 | 0.0061 | -0.46 | .647 | .721 |  |  |
| DMN-SMN (M) | -0.0121 | 0.0063 | -1.93 | .053 | .181 |  | -0.0128 | 0.0063 | -2.04 | .041 | .153 |  | -0.0145 | 0.0064 | -2.27 | .023 | .087 |  |  |
| DMN-VAN | -0.0129 | 0.0055 | -2.36 | .018 | .088 |  | -0.0089 | 0.0054 | -1.63 | .103 | .269 |  | -0.0143 | 0.0056 | -2.57 | .010 | .039 | * |  |
| DMN-VN | 0.0142 | 0.0058 | 2.43 | .015 | .078 |  | -0.0007 | 0.0058 | -0.12 | .905 | .941 |  | 0.0067 | 0.0060 | 1.13 | .260 | .397 |  |  |
| DAN-FPN | 0.0053 | 0.0055 | 0.96 | .337 | .548 |  | 0.0083 | 0.0055 | 1.52 | .128 | .296 |  | 0.0068 | 0.0056 | 1.22 | .224 | .360 |  |  |
| DAN-RTN | 0.0082 | 0.0058 | 1.42 | .156 | .320 |  | -0.0072 | 0.0058 | -1.24 | .214 | .417 |  | -0.0089 | 0.0059 | -1.51 | .130 | .268 |  |  |
| DAN-SN | 0.0057 | 0.0058 | 0.98 | .328 | .548 |  | -0.0032 | 0.0057 | -0.56 | .578 | .756 |  | -0.0116 | 0.0059 | -1.98 | .048 | .143 |  |  |
| DAN-SMN (H) | -0.0036 | 0.0062 | -0.58 | .561 | .751 |  | -0.0087 | 0.0063 | -1.38 | .168 | .352 |  | 0.0110 | 0.0064 | 1.71 | .087 | .193 |  |  |
| DAN-SMN (M) | 0.0022 | 0.0064 | 0.35 | .724 | .846 |  | 0.0084 | 0.0063 | 1.33 | .184 | .367 |  | 0.0202 | 0.0065 | 3.13 | .002 | .012 | * |  |
| DAN-VAN | -0.0022 | 0.0055 | -0.39 | .695 | .844 |  | -0.0027 | 0.0055 | -0.50 | .618 | .778 |  | -0.0059 | 0.0056 | -1.06 | .290 | .419 |  |  |
| DAN-VN | -0.0101 | 0.0060 | -1.68 | .092 | .224 |  | -0.0038 | 0.0059 | -0.64 | .525 | .706 |  | -0.0086 | 0.0061 | -1.41 | .159 | .310 |  |  |
| FPN-RTN | -0.0042 | 0.0062 | -0.68 | .499 | .718 |  | -0.0110 | 0.0061 | -1.78 | .075 | .216 |  | 0.0047 | 0.0063 | 0.75 | .450 | .576 |  |  |
| **Network pair** | **WISC Matrix** | | | | | | **Fluid** | | | | | | **Crystallized** | | | | | | |
|  | ***β*** | ***SE*** | ***t*** | ***p*** | ***p_FDR_*** |  | ***β*** | ***SE*** | ***t*** | ***p*** | ***p_FDR_*** |  | ***β*** | ***SE*** | ***t*** | ***p*** | ***p_FDR_*** |  |  |
| FPN-SN | 0.0012 | 0.0056 | 0.21 | .835 | .879 |  | 0.0062 | 0.0056 | 1.11 | .267 | .464 |  | -0.0070 | 0.0057 | -1.23 | .219 | .360 |  |  |
| FPN-SMN (H) | -0.0110 | 0.0060 | -1.83 | .068 | .201 |  | -0.0113 | 0.0060 | -1.89 | .058 | .190 |  | -0.0081 | 0.0061 | -1.32 | .187 | .337 |  |  |
| FPN-SMN (M) | -0.0135 | 0.0062 | -2.16 | .031 | .125 |  | -0.0162 | 0.0062 | -2.60 | .009 | .048 | * | -0.0173 | 0.0064 | -2.72 | .007 | .034 | * |  |
| FPN-VAN | 0.0003 | 0.0057 | 0.05 | .963 | .963 |  | 0.0047 | 0.0056 | 0.83 | .406 | .609 |  | -0.0027 | 0.0058 | -0.47 | .639 | .721 |  |  |
| FPN-VN | 0.0005 | 0.0059 | 0.08 | .934 | .946 |  | -0.0113 | 0.0059 | -1.93 | .054 | .183 |  | 0.0079 | 0.0060 | 1.31 | .190 | .337 |  |  |
| RTN-SN | -0.0028 | 0.0060 | -0.46 | .642 | .821 |  | -0.0012 | 0.0060 | -0.20 | .843 | .941 |  | 0.0074 | 0.0061 | 1.21 | .226 | .360 |  |  |
| RTN-SMN (H) | 0.0113 | 0.0063 | 1.79 | .074 | .207 |  | 0.0162 | 0.0063 | 2.57 | .010 | .050 | * | 0.0111 | 0.0064 | 1.72 | .086 | .193 |  |  |
| RTN-SMN (M) | -0.0042 | 0.0063 | -0.66 | .506 | .718 |  | 0.0035 | 0.0063 | 0.55 | .582 | .756 |  | 0.0003 | 0.0064 | 0.05 | .962 | .962 |  |  |
| RTN-VAN | -0.0088 | 0.0060 | -1.48 | .139 | .301 |  | 0.0062 | 0.0059 | 1.04 | .297 | .483 |  | -0.0021 | 0.0061 | -0.35 | .728 | .800 |  |  |
| RTN-VN | -0.0046 | 0.0056 | -0.82 | .412 | .630 |  | -0.0165 | 0.0055 | -2.99 | .003 | .020 | * | -0.0214 | 0.0057 | -3.78 | .000 | .001 | * |  |
| SN-SMN (H) | -0.0283 | 0.0064 | -4.40 | .000 | .000 | * | -0.0228 | 0.0064 | -3.56 | .000 | .007 | * | -0.0251 | 0.0066 | -3.83 | .000 | .001 | * |  |
| SN-SMN (M) | -0.0299 | 0.0065 | -4.57 | .000 | .000 | * | -0.0229 | 0.0065 | -3.52 | .000 | .007 | * | -0.0298 | 0.0067 | -4.47 | .000 | .000 | * |  |
| SN-VAN | 0.0023 | 0.0056 | 0.40 | .690 | .844 |  | -0.0040 | 0.0056 | -0.71 | .475 | .662 |  | -0.0039 | 0.0058 | -0.68 | .499 | .608 |  |  |
| SN-VN | 0.0019 | 0.0063 | 0.30 | .766 | .853 |  | -0.0001 | 0.0063 | -0.01 | .993 | .993 |  | 0.0092 | 0.0064 | 1.43 | .154 | .308 |  |  |
| SMN (H)-SMN (M) | 0.0150 | 0.0057 | 2.64 | .008 | .049 | * | 0.0216 | 0.0057 | 3.81 | .000 | .004 | * | 0.0117 | 0.0058 | 2.02 | .043 | .140 |  |  |
| SMN (H)-VAN | 0.0011 | 0.0062 | 0.18 | .857 | .879 |  | 0.0093 | 0.0062 | 1.50 | .133 | .296 |  | 0.0063 | 0.0063 | 0.99 | .322 | .443 |  |  |
| SMN (H)-VN | 0.0125 | 0.0060 | 2.09 | .037 | .140 |  | 0.0165 | 0.0060 | 2.76 | .006 | .037 | * | 0.0056 | 0.0061 | 0.91 | .361 | .477 |  |  |
| SMN (M)-VAN | -0.0074 | 0.0065 | -1.15 | .250 | .466 |  | 0.0034 | 0.0064 | 0.54 | .593 | .758 |  | -0.0056 | 0.0066 | -0.85 | .393 | .511 |  |  |
| SMN (M)-VN | 0.0107 | 0.0063 | 1.69 | .091 | .224 |  | 0.0071 | 0.0063 | 1.14 | .256 | .464 |  | 0.0127 | 0.0064 | 1.98 | .048 | .143 |  |  |
| VAN-VN | 0.0066 | 0.0061 | 1.08 | .279 | .495 |  | 0.0118 | 0.0060 | 1.96 | .050 | .179 |  | 0.0039 | 0.0062 | 0.64 | .524 | .612 |  |  |

Note. Statistics provided for age by baseline general cognitive ability interactions in models of resting-state functional connectivity development by network pair. * Significant at false-discovery rate corrected p-value (pFDR) < .050 over 66 network pairs for each cognitive measure. AN = Auditory network. CON = Cingulo-opercular network. CPN = Cingulo-parietal network. DMN = Default mode network. DAN = Dorsal attention network. FPN = Frontoparietal network. RTN = Retrosplenial network. SN = Salience network. SMN (H) = Somatomotor hand network. SMN (M) = Somatomotor mouth network. VAN = Ventral attention network. VN = Visual network.

# Table S15. Interaction between age, baseline fluid cognition and sex in whole brain gray matter structural development

| **MRI measure** | **Cognitive measure** | **Variable** | ***β*** | ***SE*** | ***df*** | ***t*** | ***p*** |
| --- | --- | --- | --- | --- | --- | --- | --- |
| Cortical surface area | Fluid cognition | Intercept | 0.3691 | 0.0283 | 26 | 13.03 | <.001 |
|  |  | Age | -0.0440 | 0.0014 | 6840 | -32.00 | <.001 |
|  |  | Cognitive ability | 0.0599 | 0.0111 | 9568 | 5.40 | <.001 |
|  |  | Sex (Female vs Male) | -0.9883 | 0.0161 | 9965 | -61.23 | <.001 |
|  |  | Baseline INR | 0.1714 | 0.0094 | 8236 | 18.32 | <.001 |
|  |  | **Age x Cognitive ability** | **-0.0043** | **0.0014** | **6325** | **-3.17** | **.002** |
|  |  | Age x Sex | -0.0588 | 0.0019 | 6494 | -30.15 | <.001 |
|  |  | Age x Baseline INR | 0.0339 | 0.0157 | 9188 | 2.16 | .031 |
|  |  | Cognitive ability x Sex | 0.0040 | 0.0010 | 6497 | 3.95 | <.001 |
|  |  | Age x Cognitive ability x Sex^ | 0.0021 | 0.0020 | 6448 | 1.07 | .283 |
|  |  | Achieva dStream* | -0.1992 | 0.0355 | 916.4 | -5.62 | <.001 |
|  |  | DISCOVERY MR750* | 0.4263 | 0.0193 | 2200 | 22.10 | <.001 |
|  |  | Ingenia* | -0.2547 | 0.0461 | 1164 | -5.53 | <.001 |
|  |  | MAGNETOM Prisma* | -0.0597 | 0.0267 | 12530 | -2.23 | .026 |
|  |  | MAGNETOM Prisma Fit* | -0.0709 | 0.0105 | 12860 | -6.75 | <.001 |
|  |  | Orchestra SDK* | 0.5114 | 0.1564 | 15140 | 3.27 | .001 |
|  |  | Prisma* | 0.0300 | 0.0061 | 14500 | 4.94 | <.001 |
|  |  | SIGNA Premier* | 0.3677 | 0.0215 | 3087 | 17.12 | <.001 |
|  |  | SIGNA UHP* | 0.3413 | 0.0214 | 3071 | 15.98 | <.001 |
| Cortical volume | Fluid cognition | Intercept | 0.3730 | 0.0346 | 24 | 10.80 | <.001 |
|  |  | Age | -0.2396 | 0.0017 | 7045 | -142.70 | <.001 |
|  |  | Cognitive ability | 0.0532 | 0.0106 | 9390 | 5.00 | <.001 |
|  |  | Sex (Female vs Male) | -0.9298 | 0.0155 | 9882 | -59.94 | <.001 |
|  |  | Baseline INR | 0.2024 | 0.0091 | 8587 | 22.28 | <.001 |
|  |  | **Age x Cognitive ability** | **-0.0035** | **0.0016** | **6516** | **-2.13** | **.033** |
|  |  | Age x Sex | -0.0928 | 0.0024 | 6695 | -39.01 | <.001 |
|  |  | Age x Baseline INR | 0.0308 | 0.0151 | 8958 | 2.04 | .041 |
|  |  | Cognitive ability x Sex | 0.0042 | 0.0012 | 6701 | 3.42 | .001 |
|  |  | Age x Cognitive ability x Sex^ | 0.0020 | 0.0024 | 6643 | 0.83 | .406 |
|  |  | Achieva dStream* | -0.2034 | 0.0430 | 1004 | -4.73 | <.001 |
|  |  | DISCOVERY MR750* | 0.1376 | 0.0235 | 2452 | 5.86 | <.001 |
|  |  | Ingenia* | -0.1227 | 0.0561 | 1237 | -2.19 | .029 |
|  |  | MAGNETOM Prisma* | -0.0446 | 0.0326 | 12560 | -1.37 | .170 |
|  |  | MAGNETOM Prisma Fit* | -0.0434 | 0.0128 | 12920 | -3.39 | .001 |
|  |  | Orchestra SDK* | 0.0420 | 0.1921 | 15240 | 0.22 | .827 |
|  |  | Prisma* | 0.0409 | 0.0074 | 14710 | 5.50 | <.001 |
|  |  | SIGNA Premier* | 0.1953 | 0.0261 | 3272 | 7.47 | <.001 |
|  |  | SIGNA UHP* | 0.1014 | 0.0260 | 3318 | 3.90 | <.001 |

Note. Bold indicates age x cognitive ability interaction of primary interest, where cognitive ability was indexed by NIH Toolbox Fluid cognition composite score at baseline. ^ The age x cognitive ability x sex interaction was not significant for between-network resting state functional connectivity or fractional anisotropy models. * Estimates are shown for each MRI model type compared to the Prisma Fit model as the reference group. INR = income-to-needs ratio. Mean FD = mean framewise displacement.

# Table S16. Interaction between age, baseline matrix reasoning and sex in whole brain white matter structural development

| **MRI measure** | **Cognitive measure** | **Variable** | ***β*** | ***SE*** | ***df*** | ***t*** | ***p*** |
| --- | --- | --- | --- | --- | --- | --- | --- |
| Fractional anisotropy | Matrix reasoning | Intercept | 0.2195 | 0.0455 | 27 | 4.82 | .000 |
|  |  | Age | 0.1718 | 0.0038 | 8146 | 45.09 | <.001 |
|  |  | Cognitive ability | 0.0084 | 0.0070 | 9169 | 1.21 | .228 |
|  |  | Sex (Female vs Male) | 0.0333 | 0.0102 | 9829 | 3.27 | .001 |
|  |  | Baseline INR | 0.0199 | 0.0059 | 8605 | 3.39 | .001 |
|  |  | Mean FD | -0.2535 | 0.0037 | 19760 | -67.66 | <.001 |
|  |  | **Age x Cognitive ability** | **-0.0088** | **0.0037** | **7630** | **-2.35** | **.019** |
|  |  | Age x Sex | 0.0076 | 0.0053 | 7493 | 1.42 | .156 |
|  |  | Age x Baseline INR | 0.0200 | 0.0100 | 9026 | 1.99 | .046 |
|  |  | Cognitive ability x Sex | 0.0108 | 0.0028 | 7608 | 3.92 | .000 |
|  |  | Age x Cognitive ability x Sex^ | 0.0051 | 0.0054 | 7584 | 0.94 | .347 |
|  |  | Achieva dStream* | -1.8660 | 0.0775 | 280 | -24.08 | <.001 |
|  |  | DISCOVERY MR750* | -0.3821 | 0.0486 | 429 | -7.87 | .000 |
|  |  | Ingenia* | -2.4570 | 0.1171 | 184 | -20.99 | <.001 |
|  |  | MAGNETOM Prisma* | 0.1206 | 0.0766 | 11890 | 1.57 | .115 |
|  |  | MAGNETOM Prisma Fit* | -0.0766 | 0.0284 | 12400 | -2.70 | .007 |
|  |  | Prisma* | 0.1889 | 0.0160 | 14290 | 11.80 | <.001 |
|  |  | SIGNA Premier* | -0.3801 | 0.0551 | 677 | -6.90 | .000 |
|  |  | SIGNA UHP* | -0.0395 | 0.0541 | 648 | -0.73 | .466 |

Note. Bold indicates age x cognitive ability interaction of primary interest, where cognitive ability was indexed by NIH Toolbox Fluid cognition composite score at baseline. ^ The age x cognitive ability x sex interaction was not significant for between-network resting state functional connectivity or fractional anisotropy models. * Estimates are shown for each MRI model type compared to the Prisma Fit model as the reference group. INR = income-to-needs ratio. Mean FD = mean framewise displacement.

# Table S17. Interaction between age, baseline general cognitive ability and sex in whole brain between-network functional connectivity development

| **MRI measure** | **Cognitive measure** | **Variable** | ***β*** | ***SE*** | ***df*** | ***t*** | ***p*** |
| --- | --- | --- | --- | --- | --- | --- | --- |
| Between-network resting-state functional connectivity | Matrix reasoning | Intercept | 0.2981 | 0.0538 | 29 | 5.54 | <.001 |
|  |  | Age | 0.0176 | 0.0083 | 7876 | 2.11 | .035 |
|  |  | Cognitive ability | -0.0118 | 0.0108 | 8969 | -1.10 | .273 |
|  |  | Sex (Female vs Male) | -0.2936 | 0.0154 | 8975 | -19.08 | <.001 |
|  |  | Baseline INR | -0.0154 | 0.0086 | 7873 | -1.79 | .074 |
|  |  | Mean FD | 0.3336 | 0.0114 | 19210 | 29.39 | <.001 |
|  |  | **Age x Cognitive ability** | **-0.0310** | **0.0081** | **7127** | **-3.84** | **.000** |
|  |  | Age x Sex | -0.0008 | 0.0113 | 6862 | -0.07 | .944 |
|  |  | Age x Baseline INR | 0.0174 | 0.0154 | 8956 | 1.13 | .258 |
|  |  | Cognitive ability x Sex | -0.0314 | 0.0059 | 6989 | -5.34 | <.001 |
|  |  | Age x Cognitive ability x Sex^ | 0.0060 | 0.0116 | 6963 | 0.52 | .607 |
|  |  | Achieva dStream* | -0.1251 | 0.1220 | 61 | -1.03 | .309 |
|  |  | DISCOVERY MR750* | -0.2297 | 0.0772 | 88 | -2.98 | .004 |
|  |  | Ingenia* | 0.3478 | 0.1787 | 43 | 1.95 | .058 |
|  |  | MAGNETOM Prisma* | 0.0758 | 0.1630 | 9788 | 0.47 | .642 |
|  |  | MAGNETOM Prisma Fit* | 0.0771 | 0.0621 | 10460 | 1.24 | .214 |
|  |  | Prisma* | -0.0373 | 0.0333 | 3543 | -1.12 | .263 |
|  |  | SIGNA Premier* | -0.1980 | 0.0964 | 200 | -2.06 | .041 |
|  |  | SIGNA UHP* | -0.0665 | 0.0928 | 183 | -0.72 | .475 |
| Between-network resting-state functional connectivity | Fluid cognition | Intercept | 0.2886 | 0.0535 | 29 | 5.40 | <.001 |
|  |  | Age | 0.0193 | 0.0084 | 7748 | 2.31 | .021 |
|  |  | Cognitive ability | -0.0315 | 0.0108 | 8973 | -2.91 | .004 |
|  |  | Sex (Female vs Male) | -0.2827 | 0.0155 | 8906 | -18.25 | <.001 |
|  |  | Baseline INR | -0.0106 | 0.0086 | 7709 | -1.23 | .217 |
|  |  | Mean FD | 0.3283 | 0.0114 | 19070 | 28.73 | <.001 |
|  |  | **Age x Cognitive ability** | **-0.0243** | **0.0081** | **6798** | **-3.01** | **.003** |
|  |  | Age x Sex | -0.0037 | 0.0114 | 6842 | -0.32 | .748 |
|  |  | Age x Baseline INR | -0.0013 | 0.0154 | 8976 | -0.08 | .935 |
|  |  | Cognitive ability x Sex | -0.0349 | 0.0059 | 6850 | -5.93 | <.001 |
|  |  | Age x Cognitive ability x Sex^ | 0.0174 | 0.0116 | 6834 | 1.49 | .136 |
|  |  | Achieva dStream* | -0.1023 | 0.1226 | 58 | -0.83 | .408 |
|  |  | DISCOVERY MR750* | -0.2035 | 0.0764 | 90 | -2.66 | .009 |
|  |  | Ingenia* | 0.4232 | 0.1700 | 52 | 2.49 | .016 |
|  |  | MAGNETOM Prisma* | 0.0454 | 0.1581 | 9557 | 0.29 | .774 |
|  |  | MAGNETOM Prisma Fit* | 0.0689 | 0.0626 | 10340 | 1.10 | .272 |
|  |  | Prisma* | -0.0454 | 0.0335 | 3366 | -1.35 | .176 |
|  |  | SIGNA Premier* | -0.1576 | 0.0952 | 202 | -1.65 | .100 |
|  |  | SIGNA UHP* | -0.0355 | 0.0923 | 192 | -0.39 | .701 |
| Between-network resting-state functional connectivity | Crystallized cognition | Intercept | 0.2910 | 0.0539 | 29 | 5.40 | <.001 |
|  |  | Age | 0.0201 | 0.0084 | 7780 | 2.39 | .017 |
|  |  | Cognitive ability | -0.0041 | 0.0111 | 8820 | -0.37 | .713 |
|  |  | Sex (Female vs Male) | -0.2845 | 0.0155 | 8915 | -18.41 | <.001 |
|  |  | Baseline INR | -0.0166 | 0.0089 | 7818 | -1.87 | .061 |
|  |  | Mean FD | 0.3303 | 0.0114 | 19090 | 29.06 | <.001 |
|  |  | **Age x Cognitive ability** | **-0.0297** | **0.0083** | **7009** | **-3.58** | **<.001** |
|  |  | Age x Sex | -0.0048 | 0.0114 | 6824 | -0.42 | .674 |
|  |  | Age x Baseline INR | 0.0051 | 0.0152 | 8866 | 0.33 | .738 |
|  |  | Cognitive ability x Sex | -0.0298 | 0.0061 | 6857 | -4.88 | <.001 |
|  |  | Age x Cognitive ability x Sex^ | 0.0112 | 0.0114 | 6813 | 0.98 | .328 |
|  |  | Achieva dStream* | -0.0951 | 0.1232 | 58 | -0.77 | .443 |
|  |  | DISCOVERY MR750* | -0.2123 | 0.0767 | 91 | -2.77 | .007 |
|  |  | Ingenia* | 0.4142 | 0.1710 | 52 | 2.42 | .019 |
|  |  | MAGNETOM Prisma* | 0.0552 | 0.1581 | 9579 | 0.35 | .727 |
|  |  | MAGNETOM Prisma Fit* | 0.0768 | 0.0627 | 10360 | 1.23 | .221 |
|  |  | Prisma* | -0.0489 | 0.0334 | 3504 | -1.46 | .144 |
|  |  | SIGNA Premier* | -0.1616 | 0.0956 | 204 | -1.69 | .092 |
|  |  | SIGNA UHP* | -0.0449 | 0.0924 | 192 | -0.49 | .628 |

Note. Bold indicates age x cognitive ability interaction of primary interest, where cognitive ability at baseline was indexed by Wechsler Intelligence Scale for Children - Matrix Reasoning, NIH Toolbox Fluid scores, and NIH Toolbox Crystallized scores. ^ The age x cognitive ability x sex interaction was not significant for between-network resting state functional connectivity models. * Estimates are shown for each MRI model type compared to the Prisma Fit model as the reference group. INR = income-to-needs ratio. Mean FD = mean framewise displacement.

# **Table S18. Models of whole brain measures of brain development and associations with baseline cognitive ability, after site exclusion**

| **Modality** | **MRI measure** | | ***β*** | ***SE*** | ***df*** | ***t*** | ***p*** |
| --- | --- | --- | --- | --- | --- | --- | --- |
| ***Matrix Reasoning*** | |  | | |  | | |
| rsfMRI | Between network FC | | **-0.0287** | **0.0060** | **6899** | **-4.82** | **.000** |
|  | Within network FC | | -0.0042 | 0.0050 | 6591 | -0.84 | .401 |
| dMRI | Fractional anisotropy | | **-0.0065** | **0.0028** | **7541** | **-2.31** | **.021** |
|  | Mean diffusivity | | 0.0003 | 0.0034 | 7527 | 0.09 | .927 |
| sMRI | Cortical thickness | | 0.0025 | 0.0027 | 6583 | 0.92 | .357 |
|  | Surface area | | 0.0009 | 0.0010 | 6528 | 0.92 | .356 |
|  | Volume | | -0.0002 | 0.0012 | 6800 | -0.20 | .840 |
| ***Fluid cognition*** | |  | | |  | | |
| rsfMRI | Between network FC | | **-0.0170** | **0.0059** | **6740** | **-2.85** | **.004** |
|  | Within network FC | | -0.0003 | 0.0050 | 6458 | -0.06 | .954 |
| dMRI | Fractional anisotropy | | 0.0003 | 0.0028 | 7266 | 0.13 | .900 |
|  | Mean diffusivity | | 0.0056 | 0.0033 | 7253 | 1.67 | .094 |
| sMRI | Cortical thickness | | 0.0043 | 0.0027 | 6351 | 1.62 | .104 |
|  | Surface area | | **-0.0033** | **0.0010** | **6344** | **-3.25** | **.001** |
|  | Volume | | **-0.0025** | **0.0012** | **6546** | **-2.05** | **.040** |
| ***Crystallized cognition*** | |  | | |  | | |
| rsfMRI | Between network FC | | **-0.0249** | **0.0061** | **6726** | **-4.10** | **.000** |
|  | Within network FC | | -0.0063 | 0.0051 | 6450 | -1.21 | .225 |
| dMRI | Fractional anisotropy | | 0.0020 | 0.0029 | 7349 | 0.70 | .483 |
|  | Mean diffusivity | | 0.0042 | 0.0034 | 7323 | 1.23 | .219 |
| sMRI | Cortical thickness | | 0.0047 | 0.0027 | 6430 | 1.72 | .086 |
|  | Surface area | | 0.0000 | 0.0010 | 6452 | -0.03 | .976 |
|  | Volume | | -0.0021 | 0.0013 | 6654 | -1.63 | .103 |

Note: Statistics provided for age by baseline general cognitive ability interactions in whole-brain models of brain development after exclusion of the smallest study site that did not participate longitudinally. General cognitive ability at baseline measured by matrix reasoning (Wechsler Intelligence Scale for Children), fluid cognition (NIH Toolbox) and crystallized cognition (NIH Toolbox), respectively. Bold indicates uncorrected p < .05. dMRI = diffusion MRI. rsfMRI = resting-state functional MRI. sMRI = structural MRI.

# Table S19. Interaction between age and baseline fluid cognition in models of gray matter structural development by region, **after site exclusion**

| **Region** | **Surface area** | | | | | **Volume** | | | | | | |
| --- | --- | --- | --- | --- | --- | --- | --- | --- | --- | --- | --- | --- |
|  | ***β*** | ***SE*** | ***t*** | ***p*** | ***p_FDR_*** |  | ***β*** | ***SE*** | ***t*** | ***p*** | ***p_FDR_*** |  |
| Banks of superior temporal sulcus | -0.0019 | 0.0015 | -1.23 | .219 | .286 |  | -0.0024 | 0.0016 | -1.47 | .143 | .223 |  |
| Caudal anterior cingulate | -0.0027 | 0.0012 | -2.25 | .024 | .094 |  | -0.0045 | 0.0013 | -3.40 | .001 | .005 | * |
| Caudal middle frontal | -0.0030 | 0.0016 | -1.83 | .068 | .153 |  | -0.0043 | 0.0016 | -2.66 | .008 | .027 | * |
| Cuneus | 0.0031 | 0.0012 | 2.62 | .009 | .060 |  | 0.0049 | 0.0014 | 3.52 | <.001 | .005 | * |
| Entorhinal | -0.0012 | 0.0022 | -0.54 | .592 | .649 |  | 0.0030 | 0.0025 | 1.22 | .223 | .330 |  |
| Fusiform | -0.0016 | 0.0011 | -1.52 | .130 | .210 |  | -0.0009 | 0.0013 | -0.69 | .491 | .618 |  |
| Isthmus cingulate | -0.0023 | 0.0012 | -1.95 | .051 | .124 |  | -0.0009 | 0.0013 | -0.69 | .490 | .618 |  |
| Insula | -0.0021 | 0.0023 | -0.89 | .373 | .437 |  | -0.0003 | 0.0021 | -0.14 | .888 | .943 |  |
| Inferior parietal | -0.0029 | 0.0013 | -2.24 | .025 | .094 |  | -0.0028 | 0.0013 | -2.14 | .033 | .101 |  |
| Inferior temporal | -0.0016 | 0.0012 | -1.34 | .182 | .264 |  | -0.0039 | 0.0013 | -3.03 | .002 | .012 | * |
| Lingual | 0.0003 | 0.0010 | 0.27 | .791 | .791 |  | 0.0018 | 0.0012 | 1.55 | .121 | .215 |  |
| Lateral orbitofrontal | -0.0055 | 0.0021 | -2.56 | .010 | .060 |  | -0.0064 | 0.0020 | -3.25 | .001 | .007 | * |
| Lateral occipital | 0.0004 | 0.0010 | 0.44 | .661 | .703 |  | 0.0018 | 0.0012 | 1.46 | .145 | .223 |  |
| Medial orbitofrontal | -0.0030 | 0.0023 | -1.30 | .194 | .264 |  | -0.0038 | 0.0023 | -1.68 | .092 | .184 |  |
| Middle temporal | -0.0023 | 0.0014 | -1.65 | .099 | .179 |  | -0.0046 | 0.0013 | -3.63 | <.001 | .005 | * |
| Paracentral | -0.0020 | 0.0014 | -1.37 | .171 | .264 |  | -0.0010 | 0.0019 | -0.54 | .589 | .715 |  |
| Pericalcarine | 0.0018 | 0.0011 | 1.60 | .109 | .185 |  | 0.0048 | 0.0014 | 3.45 | .001 | .005 | * |
| Posterior cingulate | -0.0017 | 0.0011 | -1.64 | .100 | .179 |  | -0.0009 | 0.0012 | -0.71 | .477 | .618 |  |
| Frontal pole | -0.0044 | 0.0025 | -1.78 | .074 | .158 |  | -0.0056 | 0.0028 | -2.04 | .042 | .109 |  |
| Parahippocampal | 0.0019 | 0.0019 | 1.02 | .310 | .377 |  | 0.0008 | 0.0020 | 0.38 | .701 | .822 |  |
| Pars orbitalis | -0.0035 | 0.0014 | -2.50 | .012 | .060 |  | -0.0045 | 0.0016 | -2.75 | .006 | .023 | * |
| Postcentral | -0.0020 | 0.0016 | -1.30 | .192 | .264 |  | 0.0003 | 0.0015 | 0.19 | .849 | .943 |  |
| Pars opercularis | -0.0025 | 0.0015 | -1.66 | .097 | .179 |  | -0.0025 | 0.0017 | -1.53 | .126 | .215 |  |
| Precuneus | -0.0021 | 0.0010 | -2.01 | .044 | .124 |  | 0.0001 | 0.0013 | 0.11 | .915 | .943 |  |
| Precentral | -0.0020 | 0.0017 | -1.12 | .263 | .331 |  | 0.0000 | 0.0017 | -0.01 | .992 | .992 |  |
| Pars triangularis | -0.0046 | 0.0015 | -3.14 | .002 | .029 | * | -0.0025 | 0.0016 | -1.62 | .105 | .199 |  |
| Temporal pole | -0.0048 | 0.0023 | -2.06 | .040 | .123 |  | -0.0004 | 0.0030 | -0.12 | .903 | .943 |  |
| Rostral anterior cingulate | -0.0033 | 0.0016 | -2.06 | .040 | .123 |  | -0.0031 | 0.0018 | -1.73 | .084 | .181 |  |
| Rostral middle frontal | -0.0053 | 0.0016 | -3.29 | .001 | .029 | * | -0.0059 | 0.0017 | -3.55 | <.001 | .005 | * |
| Superior frontal | -0.0041 | 0.0014 | -2.82 | .005 | .055 |  | -0.0047 | 0.0016 | -2.94 | .003 | .014 | * |
| Supramarginal | -0.0039 | 0.0015 | -2.55 | .011 | .060 |  | -0.0032 | 0.0015 | -2.08 | .038 | .108 |  |
| Superior parietal | -0.0014 | 0.0016 | -0.85 | .393 | .445 |  | 0.0031 | 0.0018 | 1.72 | .085 | .181 |  |
| Superior temporal | -0.0026 | 0.0013 | -1.97 | .049 | .124 |  | -0.0013 | 0.0013 | -0.95 | .343 | .485 |  |
| Transverse temporal | 0.0006 | 0.0014 | 0.40 | .692 | .713 |  | 0.0029 | 0.0015 | 1.88 | .060 | .145 |  |

Note. Statistics provided for age by baseline general cognitive ability interactions in regional models of surface area and volume development, after exclusion of smallest study site that did not participate longitudinally. Fluid cognition indexed by NIH Toolbox. * Significant at false-discovery rate corrected p-value (*p_FDR_*) < .050 over 68 comparisons (34 regions each for area and volume).

# Table S20. Interaction between age and baseline matrix reasoning in models of fractional anisotropy development by bilateral white matter tract, **after site exclusion**

| **White matter tract** | ***β*** | ***SE*** | ***t*** | ***p*** | ***p_FDR_*** |  |
| --- | --- | --- | --- | --- | --- | --- |
| Anterior thalamic radiations | -0.0019 | 0.0039 | -0.50 | .618 | .713 |  |
| Cingulum (cingulate) | -0.0035 | 0.0031 | -1.14 | .254 | .381 |  |
| Cingulum (parahippocampal) | -0.0002 | 0.0030 | -0.05 | .960 | .960 |  |
| Corticospinal tract or pyramidal tract | -0.0102 | 0.0033 | -3.12 | .002 | .011 | * |
| Fornix | -0.0058 | 0.0035 | -1.63 | .103 | .220 |  |
| Inferior fronto-occipital fasciculus | -0.0003 | 0.0026 | -0.12 | .907 | .960 |  |
| Inferior frontal superior frontal cortex | -0.0075 | 0.0029 | -2.54 | .011 | .033 | * |
| Inferior longitudinal fasciculus | -0.0036 | 0.0028 | -1.28 | .200 | .333 |  |
| Superior corticostriate | -0.0106 | 0.0032 | -3.29 | .001 | .011 | * |
| Striatal inferior frontal cortex | 0.0016 | 0.0027 | 0.60 | .551 | .689 |  |
| Superior longitudinal fasciculus | -0.0087 | 0.0028 | -3.07 | .002 | .011 | * |
| Uncinate fasciculus | -0.0023 | 0.0029 | -0.78 | .433 | .590 |  |
| Corpus callosum | -0.0067 | 0.0026 | -2.57 | .010 | .033 | * |
| Forceps major | -0.0055 | 0.0025 | -2.24 | .025 | .063 |  |
| Forceps minor | -0.0039 | 0.0029 | -1.35 | .176 | .330 |  |

Note. Statistics provided for age by baseline general cognitive ability interactions in tract-wise models of fractional anisotropy development, after exclusion of smallest study site that did not participate longitudinally. Matrix reasoning indexed by Wechsler Intelligence Scale for Children. * Significant at false-discovery rate corrected p-value (*p_FDR_*) < .05 over 15 white matter tracts.

# Table S21. Interaction between age and baseline general cognitive ability in models of between-network resting-state functional connectivity development by network pair, after site exclusion

| **Network pair** | **WISC Matrix** | | | | | | **Fluid** | | | | | | **Crystallized** | | | | | |  |
| --- | --- | --- | --- | --- | --- | --- | --- | --- | --- | --- | --- | --- | --- | --- | --- | --- | --- | --- | --- |
|  | ***β*** | ***SE*** | ***t*** | ***p*** | ***p_FDR_*** |  | ***β*** | ***SE*** | ***t*** | ***p*** | ***p_FDR_*** |  | ***β*** | ***SE*** | ***t*** | ***p*** | ***p_FDR_*** |  |  |
| AN-CON | -0.0257 | 0.0064 | -4.05 | .000 | .001 | * | -0.0300 | 0.0063 | -4.74 | .000 | .000 | * | -0.0280 | 0.0065 | -4.33 | .000 | .000 | * |  |
| AN-CPN | -0.0203 | 0.0063 | -3.20 | .001 | .014 | * | -0.0140 | 0.0063 | -2.22 | .027 | .110 |  | -0.0028 | 0.0065 | -0.43 | .665 | .751 |  |  |
| AN-DMN | -0.0123 | 0.0061 | -2.02 | .043 | .158 |  | -0.0069 | 0.0061 | -1.15 | .251 | .439 |  | -0.0106 | 0.0062 | -1.72 | .086 | .204 |  |  |
| AN-DAN | 0.0013 | 0.0065 | 0.20 | .845 | .878 |  | -0.0106 | 0.0065 | -1.64 | .101 | .262 |  | 0.0140 | 0.0066 | 2.11 | .035 | .119 |  |  |
| AN-FPN | -0.0104 | 0.0060 | -1.74 | .083 | .217 |  | -0.0158 | 0.0059 | -2.66 | .008 | .047 | * | -0.0102 | 0.0061 | -1.68 | .094 | .211 |  |  |
| AN-RTN | 0.0019 | 0.0061 | 0.31 | .757 | .843 |  | 0.0206 | 0.0061 | 3.38 | .001 | .008 | * | 0.0071 | 0.0063 | 1.14 | .254 | .390 |  |  |
| AN-SN | -0.0266 | 0.0062 | -4.27 | .000 | .001 | * | -0.0269 | 0.0062 | -4.34 | .000 | .001 | * | -0.0307 | 0.0063 | -4.84 | .000 | .000 | * |  |
| AN-SMN (H) | -0.0019 | 0.0061 | -0.32 | .749 | .843 |  | 0.0051 | 0.0061 | 0.84 | .399 | .590 |  | -0.0083 | 0.0062 | -1.34 | .179 | .325 |  |  |
| AN-SMN (M) | 0.0021 | 0.0062 | 0.33 | .739 | .843 |  | 0.0043 | 0.0062 | 0.70 | .483 | .673 |  | -0.0070 | 0.0063 | -1.11 | .269 | .396 |  |  |
| AN-VAN | -0.0132 | 0.0060 | -2.22 | .027 | .115 |  | -0.0010 | 0.0059 | -0.17 | .861 | .947 |  | -0.0155 | 0.0061 | -2.55 | .011 | .044 | * |  |
| AN-VN | 0.0082 | 0.0062 | 1.33 | .184 | .367 |  | 0.0195 | 0.0061 | 3.18 | .001 | .013 | * | 0.0113 | 0.0063 | 1.80 | .072 | .178 |  |  |
| CON-CPN | 0.0065 | 0.0058 | 1.12 | .262 | .476 |  | 0.0087 | 0.0058 | 1.50 | .134 | .311 |  | 0.0015 | 0.0059 | 0.25 | .803 | .835 |  |  |
| CON-DMN | -0.0203 | 0.0058 | -3.52 | .000 | .006 | * | -0.0122 | 0.0058 | -2.13 | .034 | .124 |  | -0.0263 | 0.0059 | -4.46 | .000 | .000 | * |  |
| CON-DAN | 0.0163 | 0.0059 | 2.76 | .006 | .042 | * | 0.0001 | 0.0059 | 0.02 | .984 | .984 |  | 0.0185 | 0.0060 | 3.06 | .002 | .016 | * |  |
| CON-FPN | -0.0021 | 0.0058 | -0.36 | .716 | .843 |  | -0.0051 | 0.0058 | -0.87 | .382 | .584 |  | -0.0036 | 0.0060 | -0.61 | .542 | .648 |  |  |
| CON-RTN | -0.0019 | 0.0060 | -0.31 | .756 | .843 |  | 0.0011 | 0.0059 | 0.18 | .858 | .947 |  | -0.0120 | 0.0061 | -1.97 | .049 | .146 |  |  |
| CON-SN | -0.0134 | 0.0060 | -2.24 | .025 | .115 |  | -0.0102 | 0.0060 | -1.72 | .086 | .241 |  | -0.0267 | 0.0061 | -4.37 | .000 | .000 | * |  |
| CON-SMN (H) | -0.0207 | 0.0062 | -3.36 | .001 | .009 | * | -0.0210 | 0.0062 | -3.40 | .001 | .008 | * | -0.0162 | 0.0063 | -2.57 | .010 | .044 | * |  |
| CON-SMN (M) | -0.0186 | 0.0065 | -2.85 | .004 | .034 | * | -0.0032 | 0.0065 | -0.50 | .619 | .778 |  | -0.0047 | 0.0066 | -0.71 | .478 | .592 |  |  |
| CON-VAN | -0.0100 | 0.0056 | -1.80 | .071 | .204 |  | -0.0063 | 0.0056 | -1.13 | .259 | .439 |  | -0.0171 | 0.0057 | -3.00 | .003 | .016 | * |  |
| CON-VN | 0.0064 | 0.0064 | 0.99 | .321 | .535 |  | 0.0165 | 0.0064 | 2.57 | .010 | .048 | * | 0.0174 | 0.0065 | 2.66 | .008 | .040 | * |  |
| CPN-DMN | -0.0012 | 0.0055 | -0.21 | .832 | .878 |  | 0.0013 | 0.0055 | 0.23 | .816 | .947 |  | -0.0071 | 0.0056 | -1.28 | .201 | .340 |  |  |
| **Network pair** | **WISC Matrix** | | | | | | **Fluid** | | | | | | **Crystallized** | | | | | | |
|  | ***β*** | ***SE*** | ***t*** | ***p*** | ***p_FDR_*** |  | ***β*** | ***SE*** | ***t*** | ***p*** | ***p_FDR_*** |  | ***β*** | ***SE*** | ***t*** | ***p*** | ***p_FDR_*** |  |  |
| CPN-DAN | 0.0064 | 0.0057 | 1.13 | .259 | .476 |  | -0.0008 | 0.0057 | -0.14 | .885 | .947 |  | 0.0024 | 0.0058 | 0.42 | .677 | .751 |  |  |
| CPN-FPN | -0.0037 | 0.0058 | -0.63 | .529 | .738 |  | -0.0086 | 0.0058 | -1.48 | .139 | .311 |  | -0.0085 | 0.0059 | -1.44 | .149 | .298 |  |  |
| CPN-RTN | -0.0096 | 0.0055 | -1.73 | .083 | .217 |  | -0.0181 | 0.0055 | -3.27 | .001 | .010 | * | 0.0013 | 0.0057 | 0.23 | .821 | .842 |  |  |
| CPN-SN | 0.0011 | 0.0057 | 0.20 | .842 | .878 |  | 0.0099 | 0.0057 | 1.76 | .079 | .228 |  | 0.0010 | 0.0058 | 0.17 | .866 | .877 |  |  |
| CPN-SMN (H) | -0.0230 | 0.0064 | -3.59 | .000 | .005 | * | -0.0162 | 0.0064 | -2.53 | .011 | .050 | * | -0.0066 | 0.0066 | -1.00 | .317 | .442 |  |  |
| CPN-SMN (M) | -0.0197 | 0.0065 | -3.02 | .003 | .022 | * | -0.0205 | 0.0065 | -3.15 | .002 | .013 | * | -0.0129 | 0.0067 | -1.93 | .054 | .150 |  |  |
| CPN-VAN | -0.0035 | 0.0062 | -0.58 | .565 | .747 |  | 0.0006 | 0.0062 | 0.09 | .926 | .976 |  | -0.0045 | 0.0063 | -0.71 | .478 | .592 |  |  |
| CPN-VN | 0.0093 | 0.0055 | 1.69 | .090 | .220 |  | 0.0028 | 0.0054 | 0.51 | .611 | .778 |  | 0.0093 | 0.0056 | 1.67 | .095 | .211 |  |  |
| DMN-DAN | -0.0143 | 0.0055 | -2.59 | .010 | .053 |  | -0.0063 | 0.0055 | -1.14 | .253 | .439 |  | -0.0229 | 0.0056 | -4.07 | .000 | .001 | * |  |
| DMN-FPN | -0.0155 | 0.0058 | -2.68 | .007 | .048 | * | 0.0002 | 0.0058 | 0.03 | .976 | .984 |  | -0.0202 | 0.0059 | -3.43 | .001 | .005 | * |  |
| DMN-RTN | 0.0040 | 0.0058 | 0.69 | .490 | .721 |  | 0.0097 | 0.0058 | 1.67 | .095 | .255 |  | 0.0234 | 0.0059 | 3.96 | .000 | .001 | * |  |
| DMN-SN | -0.0104 | 0.0057 | -1.82 | .069 | .204 |  | -0.0008 | 0.0057 | -0.15 | .884 | .947 |  | -0.0065 | 0.0058 | -1.13 | .260 | .390 |  |  |
| DMN-SMN (H) | -0.0072 | 0.0060 | -1.21 | .226 | .430 |  | -0.0048 | 0.0059 | -0.80 | .421 | .608 |  | -0.0025 | 0.0061 | -0.41 | .684 | .751 |  |  |
| DMN-SMN (M) | -0.0118 | 0.0063 | -1.88 | .060 | .196 |  | -0.0134 | 0.0063 | -2.13 | .033 | .124 |  | -0.0141 | 0.0064 | -2.20 | .028 | .103 |  |  |
| DMN-VAN | -0.0130 | 0.0055 | -2.37 | .018 | .086 |  | -0.0088 | 0.0055 | -1.62 | .106 | .266 |  | -0.0140 | 0.0056 | -2.51 | .012 | .047 | * |  |
| DMN-VN | 0.0144 | 0.0058 | 2.49 | .013 | .066 |  | -0.0008 | 0.0059 | -0.14 | .886 | .947 |  | 0.0064 | 0.0060 | 1.07 | .283 | .409 |  |  |
| DAN-FPN | 0.0054 | 0.0055 | 0.98 | .328 | .535 |  | 0.0083 | 0.0055 | 1.52 | .130 | .311 |  | 0.0066 | 0.0056 | 1.17 | .242 | .390 |  |  |
| DAN-RTN | 0.0081 | 0.0058 | 1.40 | .163 | .334 |  | -0.0072 | 0.0058 | -1.24 | .215 | .419 |  | -0.0083 | 0.0059 | -1.40 | .161 | .306 |  |  |
| DAN-SN | 0.0056 | 0.0058 | 0.98 | .329 | .535 |  | -0.0035 | 0.0058 | -0.61 | .544 | .719 |  | -0.0118 | 0.0059 | -2.01 | .045 | .140 |  |  |
| DAN-SMN (H) | -0.0039 | 0.0063 | -0.61 | .539 | .738 |  | -0.0085 | 0.0063 | -1.35 | .177 | .373 |  | 0.0116 | 0.0064 | 1.79 | .073 | .178 |  |  |
| DAN-SMN (M) | 0.0020 | 0.0064 | 0.32 | .752 | .843 |  | 0.0083 | 0.0063 | 1.31 | .191 | .383 |  | 0.0197 | 0.0065 | 3.04 | .002 | .016 | * |  |
| DAN-VAN | -0.0021 | 0.0055 | -0.39 | .697 | .843 |  | -0.0032 | 0.0055 | -0.58 | .561 | .729 |  | -0.0064 | 0.0056 | -1.15 | .252 | .390 |  |  |
| DAN-VN | -0.0102 | 0.0060 | -1.71 | .086 | .217 |  | -0.0039 | 0.0060 | -0.66 | .512 | .700 |  | -0.0082 | 0.0061 | -1.34 | .179 | .325 |  |  |
| FPN-RTN | -0.0042 | 0.0062 | -0.67 | .502 | .725 |  | -0.0110 | 0.0062 | -1.78 | .075 | .226 |  | 0.0048 | 0.0063 | 0.76 | .447 | .582 |  |  |
| **Network pair** | **WISC Matrix** | | | | | | **Fluid** | | | | | | **Crystallized** | | | | | | |
|  | ***β*** | ***SE*** | ***t*** | ***p*** | ***p_FDR_*** |  | ***β*** | ***SE*** | ***t*** | ***p*** | ***p_FDR_*** |  | ***β*** | ***SE*** | ***t*** | ***p*** | ***p_FDR_*** |  |  |
| FPN-SN | 0.0013 | 0.0056 | 0.23 | .815 | .878 |  | 0.0058 | 0.0056 | 1.03 | .303 | .502 |  | -0.0067 | 0.0057 | -1.18 | .239 | .390 |  |  |
| FPN-SMN (H) | -0.0113 | 0.0060 | -1.88 | .060 | .196 |  | -0.0113 | 0.0060 | -1.88 | .060 | .191 |  | -0.0080 | 0.0062 | -1.29 | .196 | .340 |  |  |
| FPN-SMN (M) | -0.0136 | 0.0063 | -2.18 | .029 | .120 |  | -0.0160 | 0.0062 | -2.57 | .010 | .048 | * | -0.0166 | 0.0064 | -2.59 | .010 | .044 | * |  |
| FPN-VAN | 0.0004 | 0.0057 | 0.06 | .948 | .960 |  | 0.0047 | 0.0056 | 0.84 | .401 | .590 |  | -0.0019 | 0.0058 | -0.33 | .741 | .781 |  |  |
| FPN-VN | 0.0003 | 0.0059 | 0.05 | .960 | .960 |  | -0.0115 | 0.0059 | -1.96 | .050 | .177 |  | 0.0077 | 0.0060 | 1.29 | .198 | .340 |  |  |
| RTN-SN | -0.0027 | 0.0060 | -0.44 | .659 | .843 |  | -0.0011 | 0.0060 | -0.18 | .857 | .947 |  | 0.0070 | 0.0061 | 1.14 | .255 | .390 |  |  |
| RTN-SMN (H) | 0.0115 | 0.0063 | 1.81 | .070 | .204 |  | 0.0162 | 0.0063 | 2.56 | .010 | .048 | * | 0.0103 | 0.0065 | 1.60 | .109 | .231 |  |  |
| RTN-SMN (M) | -0.0039 | 0.0063 | -0.62 | .537 | .738 |  | 0.0030 | 0.0063 | 0.48 | .630 | .779 |  | 0.0002 | 0.0065 | 0.03 | .980 | .980 |  |  |
| RTN-VAN | -0.0089 | 0.0060 | -1.49 | .136 | .303 |  | 0.0058 | 0.0059 | 0.98 | .326 | .509 |  | -0.0033 | 0.0061 | -0.54 | .591 | .678 |  |  |
| RTN-VN | -0.0044 | 0.0056 | -0.78 | .433 | .649 |  | -0.0168 | 0.0055 | -3.03 | .002 | .017 | * | -0.0212 | 0.0057 | -3.74 | .000 | .002 | * |  |
| SN-SMN (H) | -0.0287 | 0.0064 | -4.45 | .000 | .000 | * | -0.0230 | 0.0064 | -3.58 | .000 | .006 | * | -0.0249 | 0.0066 | -3.79 | .000 | .001 | * |  |
| SN-SMN (M) | -0.0303 | 0.0065 | -4.64 | .000 | .000 | * | -0.0232 | 0.0065 | -3.57 | .000 | .006 | * | -0.0290 | 0.0067 | -4.34 | .000 | .000 | * |  |
| SN-VAN | 0.0023 | 0.0056 | 0.40 | .687 | .843 |  | -0.0036 | 0.0056 | -0.64 | .521 | .700 |  | -0.0034 | 0.0058 | -0.59 | .557 | .648 |  |  |
| SN-VN | 0.0016 | 0.0063 | 0.25 | .799 | .878 |  | -0.0005 | 0.0063 | -0.07 | .942 | .980 |  | 0.0091 | 0.0065 | 1.41 | .160 | .306 |  |  |
| SMN (H)-SMN (M) | 0.0151 | 0.0057 | 2.65 | .008 | .048 | * | 0.0216 | 0.0057 | 3.82 | .000 | .004 | * | 0.0110 | 0.0058 | 1.90 | .058 | .155 |  |  |
| SMN (H)-VAN | 0.0011 | 0.0062 | 0.17 | .866 | .889 |  | 0.0088 | 0.0062 | 1.42 | .154 | .334 |  | 0.0060 | 0.0064 | 0.95 | .344 | .471 |  |  |
| SMN (H)-VN | 0.0125 | 0.0060 | 2.08 | .038 | .148 |  | 0.0164 | 0.0060 | 2.73 | .006 | .041 | * | 0.0045 | 0.0061 | 0.73 | .467 | .592 |  |  |
| SMN (M)-VAN | -0.0079 | 0.0065 | -1.22 | .222 | .430 |  | 0.0030 | 0.0064 | 0.47 | .639 | .779 |  | -0.0060 | 0.0066 | -0.90 | .367 | .494 |  |  |
| SMN (M)-VN | 0.0106 | 0.0063 | 1.68 | .093 | .220 |  | 0.0073 | 0.0063 | 1.15 | .249 | .439 |  | 0.0118 | 0.0064 | 1.83 | .068 | .176 |  |  |
| VAN-VN | 0.0061 | 0.0061 | 1.01 | .312 | .535 |  | 0.0114 | 0.0060 | 1.89 | .059 | .191 |  | 0.0024 | 0.0062 | 0.39 | .696 | .754 |  |  |

Note. Statistics provided for age by baseline general cognitive ability interactions in models of resting-state functional connectivity development by network pair, after exclusion of smallest study site that did not participate longitudinally. * Significant at false-discovery rate corrected p-value (pFDR) < .050 over 66 network pairs for each cognitive measure. AN = Auditory network. CON = Cingulo-opercular network. CPN = Cingulo-parietal network. DMN = Default mode network. DAN = Dorsal attention network. FPN = Frontoparietal network. RTN = Retrosplenial network. SN = Salience network. SMN (H) = Somatomotor hand network. SMN (M) = Somatomotor mouth network. VAN = Ventral attention network. VN = Visual network.

# Table S22. Interaction between age and baseline fluid cognition in models of gray matter structural development by region, adjusted for average parent educational attainment

| **Region** | **Surface area** | | | | | | **Volume** | | | | | |
| --- | --- | --- | --- | --- | --- | --- | --- | --- | --- | --- | --- | --- |
|  | ***β*** | ***SE*** | ***t*** | ***p*** | ***p_FDR_*** |  | ***β*** | ***SE*** | ***t*** | ***p*** | ***p_FDR_*** |  |
| Global | -0.0044 | 0.0010 | -4.46 | <.001 | - |  | -0.0035 | 0.0012 | -2.94 | .003 | - |  |
| Banks of superior temporal sulcus | -0.0032 | 0.0015 | -2.15 | .032 | .049 | * | -0.0036 | 0.0016 | -2.30 | .021 | .043 | * |
| Caudal anterior cingulate | -0.0027 | 0.0011 | -2.39 | .017 | .038 | * | -0.0044 | 0.0013 | -3.41 | .001 | .003 | * |
| Caudal middle frontal | -0.0044 | 0.0016 | -2.83 | .005 | .020 | * | -0.0056 | 0.0016 | -3.54 | .000 | .003 | * |
| Cuneus | 0.0032 | 0.0011 | 2.79 | .005 | .020 | * | 0.0040 | 0.0013 | 2.95 | .003 | .010 | * |
| Entorhinal | -0.0017 | 0.0021 | -0.80 | .424 | .497 |  | 0.0031 | 0.0024 | 1.31 | .191 | .283 |  |
| Fusiform | -0.0023 | 0.0010 | -2.22 | .026 | .045 | * | -0.0018 | 0.0013 | -1.36 | .174 | .268 |  |
| Isthmus cingulate | -0.0028 | 0.0012 | -2.33 | .020 | .039 | * | -0.0022 | 0.0013 | -1.68 | .094 | .152 |  |
| Insula | -0.0013 | 0.0023 | -0.59 | .557 | .592 |  | 0.0004 | 0.0020 | 0.20 | .843 | .920 |  |
| Inferior parietal | -0.0045 | 0.0012 | -3.65 | .000 | .003 | * | -0.0044 | 0.0013 | -3.49 | .000 | .003 | * |
| Inferior temporal | -0.0026 | 0.0012 | -2.25 | .024 | .043 | * | -0.0039 | 0.0013 | -3.08 | .002 | .008 | * |
| Lingual | 0.0000 | 0.0010 | 0.04 | .972 | .980 |  | 0.0005 | 0.0011 | 0.47 | .641 | .727 |  |
| Lateral orbitofrontal | -0.0054 | 0.0021 | -2.61 | .009 | .026 | * | -0.0068 | 0.0019 | -3.59 | .000 | .003 | * |
| Lateral occipital | 0.0000 | 0.0010 | -0.03 | .980 | .980 |  | 0.0008 | 0.0012 | 0.64 | .522 | .612 |  |
| Medial orbitofrontal | -0.0032 | 0.0022 | -1.42 | .154 | .188 |  | -0.0043 | 0.0022 | -1.95 | .051 | .097 |  |
| Middle temporal | -0.0035 | 0.0014 | -2.60 | .009 | .026 | * | -0.0046 | 0.0012 | -3.75 | .000 | .003 | * |
| Paracentral | -0.0028 | 0.0014 | -1.96 | .050 | .071 |  | -0.0018 | 0.0018 | -0.98 | .326 | .411 |  |
| Pericalcarine | 0.0019 | 0.0011 | 1.74 | .083 | .104 |  | 0.0040 | 0.0013 | 2.97 | .003 | .010 | * |
| Posterior cingulate | -0.0025 | 0.0010 | -2.46 | .014 | .034 | * | -0.0022 | 0.0012 | -1.92 | .055 | .098 |  |
| Frontal pole | -0.0052 | 0.0024 | -2.18 | .030 | .048 | * | -0.0069 | 0.0027 | -2.56 | .011 | .026 | * |
| Parahippocampal | 0.0014 | 0.0019 | 0.74 | .457 | .518 |  | -0.0002 | 0.0019 | -0.11 | .911 | .938 |  |
| Pars orbitalis | -0.0042 | 0.0014 | -3.10 | .002 | .011 | * | -0.0054 | 0.0016 | -3.36 | .001 | .003 | * |
| Postcentral | -0.0032 | 0.0015 | -2.09 | .037 | .055 |  | 0.0002 | 0.0015 | 0.17 | .866 | .920 |  |
| Pars opercularis | -0.0035 | 0.0015 | -2.37 | .018 | .038 | * | -0.0039 | 0.0016 | -2.39 | .017 | .038 | * |
| Precuneus | -0.0030 | 0.0010 | -3.04 | .002 | .011 | * | -0.0014 | 0.0013 | -1.06 | .287 | .390 |  |
| Precentral | -0.0039 | 0.0017 | -2.31 | .021 | .039 | * | -0.0015 | 0.0016 | -0.93 | .353 | .428 |  |
| Pars triangularis | -0.0054 | 0.0014 | -3.80 | .000 | .003 | * | -0.0040 | 0.0015 | -2.59 | .009 | .025 | * |
| Temporal pole | -0.0060 | 0.0022 | -2.67 | .008 | .026 | * | 0.0000 | 0.0030 | 0.02 | .987 | .987 |  |
| Rostral anterior cingulate | -0.0028 | 0.0016 | -1.82 | .069 | .090 |  | -0.0021 | 0.0017 | -1.22 | .222 | .315 |  |
| Rostral middle frontal | -0.0056 | 0.0016 | -3.50 | .000 | .003 | * | -0.0061 | 0.0016 | -3.71 | .000 | .003 | * |
| Superior frontal | -0.0051 | 0.0014 | -3.59 | .000 | .003 | * | -0.0056 | 0.0016 | -3.64 | .000 | .003 | * |
| Supramarginal | -0.0055 | 0.0015 | -3.62 | .000 | .003 | * | -0.0041 | 0.0015 | -2.74 | .006 | .017 | * |
| Superior parietal | -0.0030 | 0.0016 | -1.89 | .059 | .081 |  | 0.0018 | 0.0018 | 1.01 | .314 | .410 |  |
| Superior temporal | -0.0033 | 0.0013 | -2.58 | .010 | .026 | * | -0.0022 | 0.0013 | -1.71 | .087 | .147 |  |
| Transverse temporal | 0.0008 | 0.0014 | 0.61 | .545 | .592 |  | 0.0036 | 0.0015 | 2.36 | .018 | .039 | * |

Note. Statistics provided for age by baseline cognitive ability interactions in regional models of surface area and volume development adjusted for mean parent education at baseline. All regional associations between fluid cognition and brain development observed in primary analyses were significant after adjusting for parent educational attainment instead of income-to-needs ratio as the socioeconomic indicator. Fluid cognition indexed by NIH Toolbox. * Significant at false-discovery rate corrected p-value (p_FDR_)<.050 over 68 comparisons (34 regions each for area and volume).

# Table S23. Interaction between age and baseline fluid cognition in models of gray matter structural development by region, adjusted for neighborhood disadvantage

| **Region** | **Surface area** | | | | | | **Volume** | | | | | |
| --- | --- | --- | --- | --- | --- | --- | --- | --- | --- | --- | --- | --- |
|  | ***β*** | ***SE*** | ***t*** | ***p*** | ***p_FDR_*** |  | ***β*** | ***SE*** | ***t*** | ***p*** | ***p_FDR_*** |  |
| Global | -0.0031 | 0.0010 | -3.07 | .002 | - |  | -0.0026 | 0.0012 | -2.10 | .036 | - |  |
| Banks of superior temporal sulcus | -0.0016 | 0.0015 | -1.10 | .273 | .344 |  | -0.0021 | 0.0016 | -1.36 | .175 | .249 |  |
| Caudal anterior cingulate | -0.0026 | 0.0012 | -2.24 | .025 | .107 |  | -0.0046 | 0.0013 | -3.51 | .000 | .004 | * |
| Caudal middle frontal | -0.0036 | 0.0016 | -2.26 | .024 | .107 |  | -0.0045 | 0.0016 | -2.82 | .005 | .016 | * |
| Cuneus | 0.0034 | 0.0011 | 2.94 | .003 | .038 | * | 0.0049 | 0.0013 | 3.64 | .000 | .003 | * |
| Entorhinal | -0.0006 | 0.0021 | -0.29 | .773 | .797 |  | 0.0029 | 0.0024 | 1.20 | .231 | .315 |  |
| Fusiform | -0.0015 | 0.0011 | -1.40 | .162 | .251 |  | -0.0006 | 0.0013 | -0.44 | .660 | .724 |  |
| Isthmus cingulate | -0.0022 | 0.0012 | -1.84 | .065 | .196 |  | -0.0018 | 0.0013 | -1.36 | .175 | .249 |  |
| Insula | -0.0010 | 0.0023 | -0.43 | .667 | .709 |  | -0.0001 | 0.0021 | -0.05 | .963 | .963 |  |
| Inferior parietal | -0.0021 | 0.0013 | -1.68 | .092 | .202 |  | -0.0027 | 0.0013 | -2.08 | .037 | .106 |  |
| Inferior temporal | -0.0014 | 0.0012 | -1.21 | .228 | .310 |  | -0.0043 | 0.0013 | -3.35 | .001 | .005 | * |
| Lingual | -0.0002 | 0.0010 | -0.20 | .844 | .844 |  | 0.0016 | 0.0011 | 1.38 | .169 | .249 |  |
| Lateral orbitofrontal | -0.0051 | 0.0021 | -2.43 | .015 | .087 |  | -0.0071 | 0.0019 | -3.66 | .000 | .003 | * |
| Lateral occipital | 0.0016 | 0.0010 | 1.57 | .116 | .207 |  | 0.0018 | 0.0012 | 1.49 | .135 | .242 |  |
| Medial orbitofrontal | -0.0033 | 0.0023 | -1.43 | .152 | .246 |  | -0.0042 | 0.0023 | -1.85 | .064 | .162 |  |
| Middle temporal | -0.0014 | 0.0014 | -1.03 | .301 | .366 |  | -0.0040 | 0.0013 | -3.14 | .002 | .007 | * |
| Paracentral | -0.0023 | 0.0014 | -1.60 | .111 | .207 |  | -0.0021 | 0.0018 | -1.12 | .264 | .346 |  |
| Pericalcarine | 0.0020 | 0.0011 | 1.82 | .069 | .196 |  | 0.0055 | 0.0014 | 4.01 | .000 | .002 | * |
| Posterior cingulate | -0.0013 | 0.0010 | -1.21 | .226 | .310 |  | -0.0016 | 0.0012 | -1.35 | .176 | .249 |  |
| Frontal pole | -0.0050 | 0.0024 | -2.06 | .039 | .133 |  | -0.0049 | 0.0027 | -1.80 | .072 | .162 |  |
| Parahippocampal | 0.0011 | 0.0019 | 0.60 | .550 | .603 |  | -0.0011 | 0.0019 | -0.58 | .563 | .662 |  |
| Pars orbitalis | -0.0030 | 0.0014 | -2.17 | .030 | .113 |  | -0.0047 | 0.0016 | -2.90 | .004 | .014 | * |
| Postcentral | -0.0026 | 0.0016 | -1.67 | .095 | .202 |  | 0.0008 | 0.0015 | 0.55 | .584 | .662 |  |
| Pars opercularis | -0.0020 | 0.0015 | -1.32 | .186 | .274 |  | -0.0023 | 0.0017 | -1.37 | .171 | .249 |  |
| Precuneus | -0.0017 | 0.0010 | -1.72 | .086 | .202 |  | -0.0001 | 0.0013 | -0.07 | .942 | .963 |  |
| Precentral | -0.0028 | 0.0017 | -1.60 | .109 | .207 |  | -0.0009 | 0.0017 | -0.57 | .571 | .662 |  |
| Pars triangularis | -0.0041 | 0.0015 | -2.83 | .005 | .039 | * | -0.0025 | 0.0016 | -1.59 | .112 | .211 |  |
| Temporal pole | -0.0035 | 0.0023 | -1.53 | .125 | .213 |  | 0.0008 | 0.0030 | 0.25 | .800 | .850 |  |
| Rostral anterior cingulate | -0.0028 | 0.0016 | -1.74 | .083 | .202 |  | -0.0031 | 0.0017 | -1.75 | .080 | .169 |  |
| Rostral middle frontal | -0.0055 | 0.0016 | -3.39 | .001 | .024 | * | -0.0056 | 0.0017 | -3.34 | .001 | .005 | * |
| Superior frontal | -0.0046 | 0.0014 | -3.16 | .002 | .027 | * | -0.0051 | 0.0016 | -3.25 | .001 | .006 | * |
| Supramarginal | -0.0043 | 0.0015 | -2.76 | .006 | .039 | * | -0.0028 | 0.0015 | -1.82 | .069 | .162 |  |
| Superior parietal | -0.0016 | 0.0016 | -0.97 | .334 | .392 |  | 0.0031 | 0.0018 | 1.71 | .087 | .175 |  |
| Superior temporal | -0.0016 | 0.0013 | -1.18 | .239 | .313 |  | -0.0013 | 0.0013 | -1.01 | .314 | .395 |  |
| Transverse temporal | 0.0009 | 0.0014 | 0.60 | .546 | .603 |  | 0.0036 | 0.0015 | 2.36 | .018 | .056 |  |

Note. Statistics provided for age by baseline cognitive ability interactions in regional models of surface area and volume development adjusted for neighborhood disadvantage at baseline. All regional associations between fluid cognition and brain development observed in primary analyses were significant after adjusting for neighborhood disadvantage instead of income-to-needs ratio as the socioeconomic indicator. Fluid cognition indexed by NIH Toolbox. Neighbourhood disadvantage indexed by reverse-coded Area Deprivation Index. * Significant at false-discovery rate corrected p-value (p_FDR_)<.050 over 68 comparisons (34 regions each for area and volume).

# Table S24. Interaction between age and baseline matrix reasoning in models of fractional anisotropy development by bilateral white matter tract, adjusted for average parent educational attainment

| **White matter tract** | ***β*** | ***SE*** | ***t*** | ***p*** | ***p_FDR_*** |  |
| --- | --- | --- | --- | --- | --- | --- |
| Global | -0.0066 | 0.0027 | -2.42 | .015 | - |  |
| Anterior thalamic radiations | -0.0041 | 0.0037 | -1.09 | .276 | .383 |  |
| Cingulum (cingulate) | -0.0048 | 0.0030 | -1.60 | .110 | .241 |  |
| Cingulum (parahippocampal) | -0.0010 | 0.0029 | -0.35 | .726 | .726 |  |
| Corticospinal tract or pyramidal tract | -0.0091 | 0.0032 | -2.88 | .004 | .020 | * |
| Fornix | -0.0049 | 0.0034 | -1.44 | .151 | .251 |  |
| Inferior fronto-occipital fasciculus | -0.0019 | 0.0025 | -0.75 | .454 | .487 |  |
| Inferior frontal superior frontal cortex | -0.0071 | 0.0028 | -2.49 | .013 | .048 | * |
| Inferior longitudinal fasciculus | -0.0042 | 0.0027 | -1.59 | .112 | .241 |  |
| Superior corticostriate | -0.0108 | 0.0031 | -3.46 | .001 | .004 | * |
| Striatal inferior frontal cortex | 0.0020 | 0.0026 | 0.78 | .433 | .487 |  |
| Superior longitudinal fasciculus | -0.0099 | 0.0027 | -3.64 | .000 | .004 | * |
| Uncinate fasciculus | -0.0030 | 0.0028 | -1.08 | .281 | .383 |  |
| Corpus callosum | -0.0060 | 0.0025 | -2.38 | .017 | .052 |  |
| Forceps major | -0.0035 | 0.0024 | -1.48 | .138 | .251 |  |
| Forceps minor | -0.0025 | 0.0028 | -0.92 | .356 | .446 |  |

Note. Statistics provided for age by baseline general cognitive ability interactions in tract-wise models of fractional anisotropy development adjusted for mean parent education at baseline. All tract-wise associations between matrix reasoning and brain development observed in primary analyses were significant after adjusting for parent educational attainment instead of income-to-needs ratio as the socioeconomic indicator, except in the corpus callosum. Matrix reasoning indexed by Wechsler Intelligence Scale for Children. * Significant at false-discovery rate corrected p-value (*p_FDR_*) < .05 over 15 white matter tracts.

# Table S25. Interaction between age and baseline matrix reasoning in models of fractional anisotropy development by bilateral white matter tract, adjusted for neighborhood disadvantage

| **White matter tract** | ***β*** | ***SE*** | ***t*** | ***p*** | ***p_FDR_*** |  |
| --- | --- | --- | --- | --- | --- | --- |
| Global | -0.0074 | 0.0027 | -2.71 | .007 | - |  |
| Anterior thalamic radiations | -0.0072 | 0.0038 | -1.90 | .058 | .124 |  |
| Cingulum (cingulate) | -0.0044 | 0.0030 | -1.46 | .144 | .180 |  |
| Cingulum (parahippocampal) | 0.0003 | 0.0029 | 0.10 | .921 | .921 |  |
| Corticospinal tract or pyramidal tract | -0.0100 | 0.0032 | -3.14 | .002 | .006 | * |
| Fornix | -0.0053 | 0.0035 | -1.52 | .128 | .175 |  |
| Inferior fronto-occipital fasciculus | -0.0021 | 0.0026 | -0.82 | .414 | .478 |  |
| Inferior frontal superior frontal cortex | -0.0092 | 0.0029 | -3.20 | .001 | .006 | * |
| Inferior longitudinal fasciculus | -0.0047 | 0.0027 | -1.74 | .081 | .152 |  |
| Superior corticostriate | -0.0102 | 0.0032 | -3.24 | .001 | .006 | * |
| Striatal inferior frontal cortex | 0.0006 | 0.0026 | 0.22 | .827 | .886 |  |
| Superior longitudinal fasciculus | -0.0094 | 0.0028 | -3.42 | .001 | .006 | * |
| Uncinate fasciculus | -0.0043 | 0.0028 | -1.53 | .127 | .175 |  |
| Corpus callosum | -0.0075 | 0.0025 | -2.93 | .003 | .010 | * |
| Forceps major | -0.0037 | 0.0024 | -1.58 | .115 | .175 |  |
| Forceps minor | -0.0072 | 0.0028 | -2.58 | .010 | .025 | * |

Note. Statistics provided for age by baseline general cognitive ability interactions in tract-wise models of fractional anisotropy development adjusted for neighborhood disadvantage at baseline. All tract-wise associations between matrix reasoning and brain development observed in primary analyses were significant after adjusting for neighborhood disadvantage instead of income-to-needs ratio as the socioeconomic indicator. Matrix reasoning indexed by Wechsler Intelligence Scale for Children. Neighbourhood disadvantage indexed by reverse-coded Area Deprivation Index. * Significant at false-discovery rate corrected p-value (*p_FDR_*) < .05 over 15 white matter tracts.

# Table S26. Interaction between age and baseline general cognitive ability in models of between-network resting-state functional connectivity development by network pair, adjusted for average parent educational attainment

| **Network pair** | **WISC Matrix** | | | | | | **Fluid** | | | | | | **Crystallized** | | | | | | |
| --- | --- | --- | --- | --- | --- | --- | --- | --- | --- | --- | --- | --- | --- | --- | --- | --- | --- | --- | --- |
|  | ***β*** | ***SE*** | ***t*** | ***p*** | ***p_FDR_*** |  | ***β*** | ***SE*** | ***t*** | ***p*** | ***p_FDR_*** |  | ***β*** | ***SE*** | ***t*** | ***p*** | ***p_FDR_*** |  |  |
| Global | -0.0260 | 0.0057 | -4.52 | <.001 | - |  | -0.0135 | 0.0057 | -2.35 | .019 | - |  | -0.0235 | 0.0060 | -3.93 | <.001 | - |  |  |
| AN-CON | -0.0242 | 0.0061 | -3.96 | .000 | .001 | * | -0.0287 | 0.0061 | -4.72 | .000 | .000 | * | -0.0264 | 0.0063 | -4.16 | .000 | .000 | * |  |
| AN-CPN | -0.0188 | 0.0061 | -3.07 | .002 | .018 | * | -0.0145 | 0.0061 | -2.37 | .018 | .072 |  | -0.0017 | 0.0064 | -0.26 | .792 | .866 |  |  |
| AN-DMN | -0.0096 | 0.0058 | -1.64 | .100 | .313 |  | -0.0069 | 0.0058 | -1.18 | .236 | .402 |  | -0.0104 | 0.0061 | -1.72 | .086 | .211 |  |  |
| AN-DAN | 0.0026 | 0.0063 | 0.41 | .684 | .869 |  | -0.0088 | 0.0063 | -1.40 | .161 | .307 |  | 0.0141 | 0.0065 | 2.16 | .031 | .100 |  |  |
| AN-FPN | -0.0046 | 0.0058 | -0.80 | .424 | .649 |  | -0.0113 | 0.0058 | -1.97 | .049 | .148 |  | -0.0062 | 0.0060 | -1.03 | .305 | .506 |  |  |
| AN-RTN | 0.0005 | 0.0059 | 0.09 | .927 | .951 |  | 0.0220 | 0.0059 | 3.71 | .000 | .002 | * | 0.0064 | 0.0062 | 1.04 | .300 | .506 |  |  |
| AN-SN | -0.0268 | 0.0060 | -4.47 | .000 | .000 | * | -0.0294 | 0.0060 | -4.90 | .000 | .000 | * | -0.0350 | 0.0063 | -5.59 | .000 | .000 | * |  |
| AN-SMN (H) | -0.0016 | 0.0059 | -0.27 | .789 | .920 |  | 0.0079 | 0.0059 | 1.34 | .182 | .332 |  | -0.0045 | 0.0061 | -0.73 | .464 | .624 |  |  |
| AN-SMN (M) | 0.0021 | 0.0060 | 0.36 | .721 | .890 |  | 0.0060 | 0.0060 | 1.00 | .319 | .478 |  | -0.0054 | 0.0062 | -0.86 | .390 | .574 |  |  |
| AN-VAN | -0.0127 | 0.0057 | -2.22 | .027 | .122 |  | -0.0004 | 0.0057 | -0.06 | .949 | .961 |  | -0.0135 | 0.0060 | -2.27 | .023 | .083 |  |  |
| AN-VN | 0.0049 | 0.0059 | 0.83 | .405 | .645 |  | 0.0169 | 0.0059 | 2.85 | .004 | .029 | * | 0.0077 | 0.0062 | 1.25 | .210 | .399 |  |  |
| CON-CPN | 0.0056 | 0.0056 | 1.00 | .319 | .590 |  | 0.0111 | 0.0056 | 1.98 | .047 | .147 |  | 0.0052 | 0.0058 | 0.90 | .370 | .555 |  |  |
| CON-DMN | -0.0180 | 0.0055 | -3.24 | .001 | .015 | * | -0.0114 | 0.0056 | -2.05 | .041 | .132 |  | -0.0251 | 0.0058 | -4.35 | .000 | .000 | * |  |
| CON-DAN | 0.0130 | 0.0057 | 2.29 | .022 | .116 |  | -0.0010 | 0.0057 | -0.18 | .859 | .881 |  | 0.0145 | 0.0059 | 2.44 | .015 | .058 |  |  |
| CON-FPN | -0.0026 | 0.0056 | -0.46 | .646 | .869 |  | -0.0025 | 0.0056 | -0.45 | .655 | .752 |  | -0.0015 | 0.0059 | -0.25 | .799 | .866 |  |  |
| CON-RTN | -0.0012 | 0.0058 | -0.21 | .834 | .925 |  | 0.0050 | 0.0058 | 0.87 | .387 | .549 |  | -0.0110 | 0.0060 | -1.82 | .069 | .184 |  |  |
| CON-SN | -0.0171 | 0.0058 | -2.97 | .003 | .022 | * | -0.0120 | 0.0058 | -2.07 | .039 | .131 |  | -0.0295 | 0.0060 | -4.91 | .000 | .000 | * |  |
| CON-SMN (H) | -0.0191 | 0.0060 | -3.21 | .001 | .015 | * | -0.0236 | 0.0060 | -3.95 | .000 | .001 | * | -0.0195 | 0.0062 | -3.15 | .002 | .010 | * |  |
| CON-SMN (M) | -0.0112 | 0.0063 | -1.79 | .074 | .261 |  | 0.0013 | 0.0062 | 0.21 | .833 | .867 |  | -0.0013 | 0.0065 | -0.20 | .843 | .901 |  |  |
| CON-VAN | -0.0084 | 0.0053 | -1.57 | .116 | .321 |  | -0.0056 | 0.0053 | -1.05 | .292 | .446 |  | -0.0141 | 0.0056 | -2.53 | .011 | .049 | * |  |
| CON-VN | 0.0060 | 0.0062 | 0.98 | .329 | .590 |  | 0.0170 | 0.0062 | 2.75 | .006 | .036 | * | 0.0154 | 0.0064 | 2.40 | .017 | .062 |  |  |
| **Network pair** | **WISC Matrix** | | | | | | **Fluid** | | | | | | **Crystallized** | | | | | | |
|  | ***β*** | ***SE*** | ***t*** | ***p*** | ***p_FDR_*** |  | ***β*** | ***SE*** | ***t*** | ***p*** | ***p_FDR_*** |  | ***β*** | ***SE*** | ***t*** | ***p*** | ***p_FDR_*** |  |  |
| CPN-DMN | -0.0003 | 0.0052 | -0.05 | .961 | .961 |  | 0.0022 | 0.0053 | 0.42 | .673 | .757 |  | -0.0079 | 0.0055 | -1.43 | .151 | .337 |  |  |
| CPN-DAN | 0.0022 | 0.0055 | 0.40 | .691 | .869 |  | -0.0026 | 0.0055 | -0.47 | .641 | .752 |  | 0.0001 | 0.0057 | 0.02 | .987 | .987 |  |  |
| CPN-FPN | -0.0042 | 0.0056 | -0.75 | .453 | .680 |  | -0.0092 | 0.0056 | -1.63 | .103 | .218 |  | -0.0105 | 0.0058 | -1.80 | .072 | .188 |  |  |
| CPN-RTN | -0.0083 | 0.0053 | -1.56 | .119 | .321 |  | -0.0172 | 0.0053 | -3.22 | .001 | .010 | * | 0.0009 | 0.0055 | 0.17 | .867 | .913 |  |  |
| CPN-SN | 0.0044 | 0.0055 | 0.80 | .424 | .649 |  | 0.0148 | 0.0055 | 2.70 | .007 | .038 | * | 0.0082 | 0.0057 | 1.44 | .151 | .337 |  |  |
| CPN-SMN (H) | -0.0212 | 0.0062 | -3.44 | .001 | .009 | * | -0.0165 | 0.0062 | -2.65 | .008 | .041 | * | -0.0061 | 0.0065 | -0.94 | .350 | .547 |  |  |
| CPN-SMN (M) | -0.0199 | 0.0063 | -3.18 | .001 | .015 | * | -0.0208 | 0.0063 | -3.31 | .001 | .009 | * | -0.0137 | 0.0065 | -2.09 | .036 | .109 |  |  |
| CPN-VAN | 0.0026 | 0.0059 | 0.44 | .661 | .869 |  | 0.0038 | 0.0060 | 0.64 | .524 | .692 |  | 0.0001 | 0.0062 | 0.02 | .983 | .987 |  |  |
| CPN-VN | 0.0111 | 0.0053 | 2.11 | .035 | .136 |  | 0.0022 | 0.0053 | 0.41 | .679 | .757 |  | 0.0097 | 0.0055 | 1.78 | .076 | .190 |  |  |
| DMN-DAN | -0.0130 | 0.0053 | -2.43 | .015 | .085 |  | -0.0048 | 0.0054 | -0.91 | .365 | .527 |  | -0.0222 | 0.0056 | -3.99 | .000 | .001 | * |  |
| DMN-FPN | -0.0124 | 0.0056 | -2.23 | .026 | .122 |  | 0.0025 | 0.0056 | 0.45 | .653 | .752 |  | -0.0191 | 0.0058 | -3.29 | .001 | .007 | * |  |
| DMN-RTN | 0.0060 | 0.0056 | 1.08 | .279 | .548 |  | 0.0100 | 0.0056 | 1.80 | .072 | .182 |  | 0.0222 | 0.0058 | 3.82 | .000 | .001 | * |  |
| DMN-SN | -0.0050 | 0.0055 | -0.91 | .364 | .605 |  | 0.0034 | 0.0055 | 0.62 | .533 | .693 |  | -0.0019 | 0.0057 | -0.33 | .743 | .840 |  |  |
| DMN-SMN (H) | -0.0054 | 0.0057 | -0.93 | .352 | .596 |  | -0.0066 | 0.0058 | -1.15 | .251 | .407 |  | -0.0021 | 0.0060 | -0.34 | .732 | .840 |  |  |
| DMN-SMN (M) | -0.0130 | 0.0061 | -2.15 | .031 | .128 |  | -0.0143 | 0.0061 | -2.35 | .019 | .072 |  | -0.0155 | 0.0063 | -2.46 | .014 | .057 |  |  |
| DMN-VAN | -0.0143 | 0.0053 | -2.71 | .007 | .044 | * | -0.0092 | 0.0053 | -1.74 | .082 | .197 |  | -0.0145 | 0.0055 | -2.65 | .008 | .040 | * |  |
| DMN-VN | 0.0121 | 0.0056 | 2.16 | .031 | .128 |  | -0.0022 | 0.0056 | -0.39 | .697 | .765 |  | 0.0040 | 0.0059 | 0.68 | .498 | .636 |  |  |
| DAN-FPN | 0.0028 | 0.0053 | 0.53 | .599 | .834 |  | 0.0081 | 0.0053 | 1.53 | .127 | .249 |  | 0.0050 | 0.0055 | 0.90 | .369 | .555 |  |  |
| DAN-RTN | 0.0076 | 0.0056 | 1.36 | .173 | .409 |  | -0.0085 | 0.0056 | -1.52 | .128 | .249 |  | -0.0074 | 0.0058 | -1.28 | .200 | .391 |  |  |
| DAN-SN | 0.0009 | 0.0056 | 0.16 | .876 | .925 |  | -0.0032 | 0.0056 | -0.57 | .570 | .706 |  | -0.0150 | 0.0058 | -2.57 | .010 | .046 | * |  |
| DAN-SMN (H) | -0.0015 | 0.0061 | -0.25 | .802 | .920 |  | -0.0096 | 0.0061 | -1.57 | .117 | .240 |  | 0.0118 | 0.0064 | 1.86 | .064 | .177 |  |  |
| DAN-SMN (M) | 0.0053 | 0.0061 | 0.87 | .386 | .628 |  | 0.0103 | 0.0061 | 1.69 | .091 | .210 |  | 0.0212 | 0.0064 | 3.32 | .001 | .006 | * |  |
| DAN-VAN | 0.0011 | 0.0053 | 0.21 | .832 | .925 |  | 0.0003 | 0.0053 | 0.05 | .962 | .962 |  | -0.0043 | 0.0055 | -0.77 | .441 | .614 |  |  |
| DAN-VN | -0.0098 | 0.0057 | -1.71 | .087 | .284 |  | -0.0033 | 0.0058 | -0.57 | .567 | .706 |  | -0.0046 | 0.0060 | -0.77 | .438 | .614 |  |  |
| **Network pair** | **WISC Matrix** | | | | | | **Fluid** | | | | | | **Crystallized** | | | | | | |
|  | ***β*** | ***SE*** | ***t*** | ***p*** | ***p_FDR_*** |  | ***β*** | ***SE*** | ***t*** | ***p*** | ***p_FDR_*** |  | ***β*** | ***SE*** | ***t*** | ***p*** | ***p_FDR_*** |  |  |
| FPN-RTN | -0.0031 | 0.0059 | -0.53 | .598 | .834 |  | -0.0103 | 0.0059 | -1.73 | .083 | .197 |  | 0.0064 | 0.0062 | 1.04 | .299 | .506 |  |  |
| FPN-SN | 0.0058 | 0.0054 | 1.08 | .281 | .548 |  | 0.0103 | 0.0054 | 1.90 | .057 | .164 |  | -0.0025 | 0.0056 | -0.45 | .653 | .772 |  |  |
| FPN-SMN (H) | -0.0073 | 0.0058 | -1.25 | .212 | .472 |  | -0.0107 | 0.0058 | -1.83 | .067 | .175 |  | -0.0043 | 0.0061 | -0.71 | .480 | .624 |  |  |
| FPN-SMN (M) | -0.0115 | 0.0060 | -1.90 | .057 | .212 |  | -0.0158 | 0.0061 | -2.61 | .009 | .044 | * | -0.0181 | 0.0063 | -2.88 | .004 | .022 | * |  |
| FPN-VAN | 0.0014 | 0.0055 | 0.26 | .798 | .920 |  | 0.0061 | 0.0055 | 1.11 | .268 | .418 |  | -0.0027 | 0.0057 | -0.47 | .636 | .766 |  |  |
| FPN-VN | -0.0020 | 0.0057 | -0.35 | .730 | .890 |  | -0.0130 | 0.0057 | -2.28 | .023 | .084 |  | 0.0063 | 0.0059 | 1.06 | .291 | .506 |  |  |
| RTN-SN | -0.0008 | 0.0058 | -0.14 | .885 | .925 |  | 0.0021 | 0.0058 | 0.37 | .714 | .768 |  | 0.0072 | 0.0060 | 1.20 | .232 | .420 |  |  |
| RTN-SMN (H) | 0.0075 | 0.0061 | 1.23 | .218 | .472 |  | 0.0150 | 0.0061 | 2.46 | .014 | .060 |  | 0.0094 | 0.0064 | 1.48 | .138 | .325 |  |  |
| RTN-SMN (M) | -0.0062 | 0.0061 | -1.01 | .310 | .590 |  | 0.0050 | 0.0061 | 0.82 | .415 | .568 |  | -0.0007 | 0.0064 | -0.11 | .916 | .952 |  |  |
| RTN-VAN | -0.0042 | 0.0058 | -0.73 | .466 | .686 |  | 0.0095 | 0.0058 | 1.65 | .098 | .214 |  | 0.0001 | 0.0060 | 0.02 | .986 | .987 |  |  |
| RTN-VN | -0.0060 | 0.0054 | -1.12 | .263 | .548 |  | -0.0176 | 0.0053 | -3.29 | .001 | .009 | * | -0.0199 | 0.0056 | -3.58 | .000 | .003 | * |  |
| SN-SMN (H) | -0.0301 | 0.0062 | -4.84 | .000 | .000 | * | -0.0297 | 0.0062 | -4.77 | .000 | .000 | * | -0.0327 | 0.0065 | -5.04 | .000 | .000 | * |  |
| SN-SMN (M) | -0.0307 | 0.0063 | -4.86 | .000 | .000 | * | -0.0282 | 0.0063 | -4.46 | .000 | .000 | * | -0.0355 | 0.0066 | -5.39 | .000 | .000 | * |  |
| SN-VAN | 0.0008 | 0.0054 | 0.14 | .888 | .925 |  | -0.0064 | 0.0054 | -1.18 | .237 | .402 |  | -0.0080 | 0.0057 | -1.42 | .156 | .338 |  |  |
| SN-VN | -0.0008 | 0.0061 | -0.14 | .890 | .925 |  | -0.0040 | 0.0061 | -0.65 | .516 | .692 |  | 0.0059 | 0.0063 | 0.93 | .351 | .547 |  |  |
| SMN (H)-SMN (M) | 0.0167 | 0.0055 | 3.04 | .002 | .018 | * | 0.0245 | 0.0055 | 4.45 | .000 | .000 | * | 0.0158 | 0.0057 | 2.76 | .006 | .030 | * |  |
| SMN (H)-VAN | 0.0010 | 0.0060 | 0.16 | .870 | .925 |  | 0.0076 | 0.0060 | 1.26 | .207 | .368 |  | 0.0083 | 0.0063 | 1.32 | .186 | .383 |  |  |
| SMN (H)-VN | 0.0085 | 0.0058 | 1.46 | .144 | .362 |  | 0.0168 | 0.0058 | 2.89 | .004 | .028 | * | 0.0029 | 0.0061 | 0.47 | .638 | .766 |  |  |
| SMN (M)-VAN | -0.0098 | 0.0062 | -1.57 | .117 | .321 |  | 0.0037 | 0.0062 | 0.59 | .555 | .706 |  | -0.0046 | 0.0065 | -0.72 | .475 | .624 |  |  |
| SMN (M)-VN | 0.0095 | 0.0061 | 1.56 | .119 | .321 |  | 0.0056 | 0.0061 | 0.92 | .356 | .524 |  | 0.0121 | 0.0063 | 1.91 | .057 | .164 |  |  |
| VAN-VN | 0.0063 | 0.0058 | 1.09 | .276 | .548 |  | 0.0127 | 0.0058 | 2.19 | .029 | .101 |  | 0.0049 | 0.0061 | 0.81 | .417 | .602 |  |  |

Note. Statistics provided for age by baseline general cognitive ability interactions in models of resting-state functional connectivity development by network pair, after adjustment for mean parent educational attainment at baseline. All network pair associations between general cognitive ability and brain development observed in primary analyses were significant after adjusting for parent education instead of income-to-needs ratio as the socioeconomic indicator, except CON-DAN, CON-SMN (M), DMN-DAN and DMN-FPN for matrix reasoning, AN-FPN and RTN-SMN (H) for fluid cognition, AN-VAN, CON-DAN and CON-VN for crystallized cognition. * Significant at false-discovery rate corrected p-value (pFDR) < .050 over 66 network pairs for each cognitive measure. AN = Auditory network. CON = Cingulo-opercular network. CPN = Cingulo-parietal network. DMN = Default mode network. DAN = Dorsal attention network. FPN = Frontoparietal network. RTN = Retrosplenial network. SN = Salience network. SMN (H) = Somatomotor hand network. SMN (M) = Somatomotor mouth network. VAN = Ventral attention network. VN = Visual network.

# Table S27. Interaction between age and baseline general cognitive ability in models of between-network resting-state functional connectivity development by network pair, adjusted for neighborhood disadvantage

| **Network pair** | **WISC Matrix** | | | | | | **Fluid** | | | | | | **Crystallized** | | | | | | |
| --- | --- | --- | --- | --- | --- | --- | --- | --- | --- | --- | --- | --- | --- | --- | --- | --- | --- | --- | --- |
|  | ***β*** | ***SE*** | ***t*** | ***p*** | ***p_FDR_*** |  | ***β*** | ***SE*** | ***t*** | ***p*** | ***p_FDR_*** |  | ***β*** | ***SE*** | ***t*** | ***p*** | ***p_FDR_*** |  |  |
| Global | -0.0277 | 0.0058 | -4.80 | <.001 | - |  | -0.0127 | 0.0058 | -2.18 | .029 | - |  | -0.0240 | 0.0058 | -4.11 | <.001 | - |  |  |
| AN-CON | -0.0264 | 0.0061 | -4.30 | .000 | .000 | * | -0.0272 | 0.0062 | -4.38 | .000 | .000 | * | -0.0275 | 0.0062 | -4.42 | .000 | .000 | * |  |
| AN-CPN | -0.0174 | 0.0062 | -2.82 | .005 | .034 | * | -0.0120 | 0.0062 | -1.92 | .055 | .165 |  | -0.0031 | 0.0063 | -0.49 | .622 | .694 |  |  |
| AN-DMN | -0.0101 | 0.0059 | -1.71 | .087 | .227 |  | -0.0071 | 0.0059 | -1.19 | .233 | .435 |  | -0.0143 | 0.0060 | -2.40 | .017 | .052 |  |  |
| AN-DAN | 0.0030 | 0.0063 | 0.47 | .638 | .766 |  | -0.0090 | 0.0064 | -1.42 | .157 | .366 |  | 0.0168 | 0.0064 | 2.62 | .009 | .033 | * |  |
| AN-FPN | -0.0071 | 0.0058 | -1.22 | .222 | .427 |  | -0.0144 | 0.0059 | -2.45 | .014 | .058 |  | -0.0113 | 0.0059 | -1.92 | .055 | .130 |  |  |
| AN-RTN | 0.0005 | 0.0060 | 0.09 | .927 | .964 |  | 0.0202 | 0.0060 | 3.34 | .001 | .008 | * | 0.0055 | 0.0061 | 0.91 | .365 | .482 |  |  |
| AN-SN | -0.0283 | 0.0060 | -4.69 | .000 | .000 | * | -0.0267 | 0.0061 | -4.38 | .000 | .000 | * | -0.0351 | 0.0061 | -5.74 | .000 | .000 | * |  |
| AN-SMN (H) | 0.0017 | 0.0060 | 0.28 | .779 | .868 |  | 0.0074 | 0.0060 | 1.24 | .216 | .422 |  | 0.0003 | 0.0060 | 0.05 | .959 | .971 |  |  |
| AN-SMN (M) | 0.0054 | 0.0060 | 0.89 | .373 | .563 |  | 0.0080 | 0.0061 | 1.32 | .186 | .392 |  | -0.0010 | 0.0061 | -0.16 | .871 | .944 |  |  |
| AN-VAN | -0.0140 | 0.0058 | -2.41 | .016 | .075 |  | -0.0002 | 0.0059 | -0.04 | .972 | .988 |  | -0.0142 | 0.0059 | -2.42 | .016 | .050 |  |  |
| AN-VN | 0.0077 | 0.0059 | 1.29 | .198 | .396 |  | 0.0193 | 0.0060 | 3.22 | .001 | .008 | * | 0.0137 | 0.0060 | 2.28 | .023 | .066 |  |  |
| CON-CPN | 0.0055 | 0.0056 | 0.98 | .329 | .524 |  | 0.0131 | 0.0057 | 2.31 | .021 | .074 |  | 0.0064 | 0.0057 | 1.11 | .265 | .385 |  |  |
| CON-DMN | -0.0182 | 0.0056 | -3.25 | .001 | .011 | * | -0.0111 | 0.0057 | -1.95 | .051 | .160 |  | -0.0268 | 0.0057 | -4.72 | .000 | .000 | * |  |
| CON-DAN | 0.0142 | 0.0057 | 2.48 | .013 | .074 |  | -0.0009 | 0.0058 | -0.16 | .873 | .924 |  | 0.0169 | 0.0058 | 2.90 | .004 | .017 | * |  |
| CON-FPN | -0.0060 | 0.0057 | -1.06 | .291 | .491 |  | -0.0062 | 0.0058 | -1.07 | .285 | .463 |  | -0.0073 | 0.0058 | -1.26 | .209 | .320 |  |  |
| CON-RTN | -0.0023 | 0.0058 | -0.40 | .689 | .779 |  | 0.0048 | 0.0059 | 0.81 | .419 | .581 |  | -0.0106 | 0.0059 | -1.79 | .073 | .147 |  |  |
| CON-SN | -0.0193 | 0.0058 | -3.32 | .001 | .011 | * | -0.0118 | 0.0059 | -2.01 | .045 | .146 |  | -0.0305 | 0.0059 | -5.19 | .000 | .000 | * |  |
| CON-SMN (H) | -0.0192 | 0.0060 | -3.20 | .001 | .011 | * | -0.0218 | 0.0061 | -3.58 | .000 | .005 | * | -0.0158 | 0.0061 | -2.59 | .010 | .034 | * |  |
| CON-SMN (M) | -0.0119 | 0.0063 | -1.88 | .060 | .186 |  | 0.0017 | 0.0064 | 0.27 | .786 | .878 |  | -0.0019 | 0.0064 | -0.30 | .768 | .844 |  |  |
| CON-VAN | -0.0093 | 0.0054 | -1.73 | .084 | .227 |  | -0.0062 | 0.0055 | -1.13 | .259 | .449 |  | -0.0152 | 0.0055 | -2.78 | .005 | .022 | * |  |
| CON-VN | 0.0099 | 0.0062 | 1.59 | .112 | .264 |  | 0.0207 | 0.0063 | 3.28 | .001 | .008 | * | 0.0215 | 0.0063 | 3.41 | .001 | .005 | * |  |
| **Network pair** | **WISC Matrix** | | | | | | **Fluid** | | | | | | **Crystallized** | | | | | | |
|  | ***β*** | ***SE*** | ***t*** | ***p*** | ***p_FDR_*** |  | ***β*** | ***SE*** | ***t*** | ***p*** | ***p_FDR_*** |  | ***β*** | ***SE*** | ***t*** | ***p*** | ***p_FDR_*** |  |  |
| CPN-DMN | -0.0032 | 0.0053 | -0.60 | .549 | .726 |  | -0.0025 | 0.0054 | -0.46 | .644 | .784 |  | -0.0084 | 0.0054 | -1.56 | .119 | .221 |  |  |
| CPN-DAN | 0.0051 | 0.0055 | 0.93 | .355 | .554 |  | 0.0011 | 0.0056 | 0.20 | .842 | .924 |  | 0.0006 | 0.0056 | 0.11 | .909 | .948 |  |  |
| CPN-FPN | -0.0041 | 0.0057 | -0.72 | .471 | .667 |  | -0.0089 | 0.0057 | -1.54 | .122 | .298 |  | -0.0074 | 0.0058 | -1.28 | .200 | .311 |  |  |
| CPN-RTN | -0.0098 | 0.0054 | -1.83 | .068 | .196 |  | -0.0176 | 0.0054 | -3.25 | .001 | .008 | * | -0.0031 | 0.0054 | -0.58 | .564 | .637 |  |  |
| CPN-SN | 0.0029 | 0.0055 | 0.53 | .597 | .739 |  | 0.0139 | 0.0056 | 2.50 | .013 | .054 |  | 0.0084 | 0.0056 | 1.50 | .133 | .236 |  |  |
| CPN-SMN (H) | -0.0218 | 0.0062 | -3.50 | .000 | .007 | * | -0.0140 | 0.0063 | -2.20 | .028 | .094 |  | -0.0096 | 0.0064 | -1.51 | .131 | .236 |  |  |
| CPN-SMN (M) | -0.0203 | 0.0063 | -3.21 | .001 | .011 | * | -0.0186 | 0.0064 | -2.89 | .004 | .020 | * | -0.0132 | 0.0064 | -2.06 | .040 | .111 |  |  |
| CPN-VAN | 0.0000 | 0.0060 | 0.00 | .996 | .996 |  | -0.0009 | 0.0061 | -0.16 | .876 | .924 |  | -0.0001 | 0.0061 | -0.02 | .987 | .987 |  |  |
| CPN-VN | 0.0129 | 0.0053 | 2.42 | .016 | .075 |  | 0.0050 | 0.0054 | 0.93 | .355 | .512 |  | 0.0100 | 0.0054 | 1.85 | .064 | .135 |  |  |
| DMN-DAN | -0.0129 | 0.0054 | -2.40 | .016 | .075 |  | -0.0044 | 0.0055 | -0.80 | .424 | .581 |  | -0.0236 | 0.0055 | -4.34 | .000 | .000 | * |  |
| DMN-FPN | -0.0129 | 0.0056 | -2.30 | .021 | .093 |  | -0.0002 | 0.0057 | -0.03 | .975 | .988 |  | -0.0207 | 0.0057 | -3.64 | .000 | .002 | * |  |
| DMN-RTN | 0.0050 | 0.0056 | 0.89 | .375 | .563 |  | 0.0088 | 0.0057 | 1.55 | .122 | .298 |  | 0.0193 | 0.0057 | 3.38 | .001 | .005 | * |  |
| DMN-SN | -0.0073 | 0.0055 | -1.33 | .184 | .378 |  | 0.0001 | 0.0056 | 0.01 | .989 | .989 |  | -0.0045 | 0.0056 | -0.80 | .424 | .525 |  |  |
| DMN-SMN (H) | -0.0033 | 0.0058 | -0.57 | .569 | .739 |  | -0.0081 | 0.0059 | -1.38 | .167 | .372 |  | -0.0046 | 0.0059 | -0.78 | .436 | .532 |  |  |
| DMN-SMN (M) | -0.0132 | 0.0061 | -2.16 | .031 | .110 |  | -0.0154 | 0.0062 | -2.50 | .012 | .054 |  | -0.0162 | 0.0062 | -2.62 | .009 | .033 | * |  |
| DMN-VAN | -0.0141 | 0.0053 | -2.65 | .008 | .052 |  | -0.0102 | 0.0054 | -1.90 | .057 | .165 |  | -0.0171 | 0.0054 | -3.17 | .002 | .008 | * |  |
| DMN-VN | 0.0128 | 0.0057 | 2.25 | .025 | .097 |  | 0.0015 | 0.0058 | 0.27 | .788 | .878 |  | 0.0057 | 0.0058 | 0.98 | .326 | .446 |  |  |
| DAN-FPN | 0.0029 | 0.0053 | 0.55 | .580 | .739 |  | 0.0069 | 0.0054 | 1.28 | .202 | .415 |  | 0.0035 | 0.0054 | 0.65 | .516 | .601 |  |  |
| DAN-RTN | 0.0102 | 0.0056 | 1.83 | .067 | .196 |  | -0.0062 | 0.0057 | -1.09 | .277 | .459 |  | -0.0038 | 0.0057 | -0.67 | .505 | .597 |  |  |
| DAN-SN | 0.0002 | 0.0056 | 0.03 | .976 | .989 |  | -0.0041 | 0.0057 | -0.72 | .473 | .615 |  | -0.0163 | 0.0057 | -2.86 | .004 | .019 | * |  |
| DAN-SMN (H) | -0.0025 | 0.0061 | -0.41 | .684 | .779 |  | -0.0100 | 0.0062 | -1.61 | .107 | .279 |  | 0.0118 | 0.0062 | 1.90 | .058 | .132 |  |  |
| DAN-SMN (M) | 0.0029 | 0.0062 | 0.48 | .635 | .766 |  | 0.0071 | 0.0062 | 1.13 | .257 | .449 |  | 0.0185 | 0.0063 | 2.96 | .003 | .015 | * |  |
| DAN-VAN | -0.0022 | 0.0054 | -0.40 | .686 | .779 |  | -0.0024 | 0.0054 | -0.44 | .661 | .784 |  | -0.0078 | 0.0054 | -1.45 | .147 | .255 |  |  |
| DAN-VN | -0.0152 | 0.0058 | -2.62 | .009 | .053 |  | -0.0059 | 0.0059 | -0.99 | .320 | .489 |  | -0.0116 | 0.0059 | -1.97 | .049 | .120 |  |  |
| **Network pair** | **WISC Matrix** | | | | | | **Fluid** | | | | | | **Crystallized** | | | | | | |
|  | ***β*** | ***SE*** | ***t*** | ***p*** | ***p_FDR_*** |  | ***β*** | ***SE*** | ***t*** | ***p*** | ***p_FDR_*** |  | ***β*** | ***SE*** | ***t*** | ***p*** | ***p_FDR_*** |  |  |
| FPN-RTN | -0.0013 | 0.0060 | -0.22 | .828 | .897 |  | -0.0103 | 0.0060 | -1.70 | .089 | .239 |  | 0.0058 | 0.0061 | 0.96 | .340 | .457 |  |  |
| FPN-SN | 0.0035 | 0.0055 | 0.63 | .526 | .708 |  | 0.0058 | 0.0055 | 1.05 | .293 | .467 |  | -0.0079 | 0.0055 | -1.43 | .152 | .257 |  |  |
| FPN-SMN (H) | -0.0095 | 0.0059 | -1.61 | .108 | .264 |  | -0.0138 | 0.0060 | -2.31 | .021 | .074 |  | -0.0106 | 0.0060 | -1.77 | .077 | .150 |  |  |
| FPN-SMN (M) | -0.0127 | 0.0061 | -2.09 | .036 | .124 |  | -0.0178 | 0.0062 | -2.89 | .004 | .020 | * | -0.0211 | 0.0062 | -3.41 | .001 | .005 | * |  |
| FPN-VAN | 0.0010 | 0.0055 | 0.17 | .861 | .920 |  | 0.0041 | 0.0056 | 0.74 | .459 | .610 |  | -0.0046 | 0.0056 | -0.82 | .412 | .525 |  |  |
| FPN-VN | -0.0031 | 0.0057 | -0.54 | .592 | .739 |  | -0.0103 | 0.0058 | -1.78 | .075 | .209 |  | 0.0047 | 0.0058 | 0.80 | .422 | .525 |  |  |
| RTN-SN | -0.0026 | 0.0058 | -0.45 | .656 | .776 |  | 0.0030 | 0.0059 | 0.51 | .609 | .766 |  | 0.0073 | 0.0059 | 1.23 | .219 | .328 |  |  |
| RTN-SMN (H) | 0.0073 | 0.0062 | 1.18 | .238 | .431 |  | 0.0166 | 0.0062 | 2.67 | .008 | .038 | * | 0.0117 | 0.0062 | 1.87 | .061 | .135 |  |  |
| RTN-SMN (M) | -0.0074 | 0.0062 | -1.20 | .231 | .428 |  | 0.0027 | 0.0062 | 0.44 | .663 | .784 |  | -0.0004 | 0.0063 | -0.06 | .953 | .971 |  |  |
| RTN-VAN | -0.0099 | 0.0058 | -1.71 | .087 | .227 |  | 0.0061 | 0.0059 | 1.04 | .300 | .468 |  | -0.0060 | 0.0059 | -1.01 | .311 | .434 |  |  |
| RTN-VN | -0.0079 | 0.0054 | -1.45 | .148 | .330 |  | -0.0189 | 0.0055 | -3.45 | .001 | .006 | * | -0.0209 | 0.0055 | -3.82 | .000 | .001 | * |  |
| SN-SMN (H) | -0.0283 | 0.0062 | -4.53 | .000 | .000 | * | -0.0262 | 0.0063 | -4.15 | .000 | .001 | * | -0.0275 | 0.0063 | -4.34 | .000 | .000 | * |  |
| SN-SMN (M) | -0.0312 | 0.0064 | -4.90 | .000 | .000 | * | -0.0240 | 0.0064 | -3.74 | .000 | .003 | * | -0.0322 | 0.0064 | -5.00 | .000 | .000 | * |  |
| SN-VAN | 0.0014 | 0.0055 | 0.26 | .796 | .875 |  | -0.0078 | 0.0055 | -1.41 | .160 | .366 |  | -0.0074 | 0.0056 | -1.34 | .181 | .288 |  |  |
| SN-VN | 0.0040 | 0.0062 | 0.65 | .514 | .703 |  | 0.0019 | 0.0062 | 0.30 | .761 | .873 |  | 0.0126 | 0.0062 | 2.03 | .043 | .111 |  |  |
| SMN (H)-SMN (M) | 0.0177 | 0.0055 | 3.19 | .001 | .011 | * | 0.0230 | 0.0056 | 4.10 | .000 | .001 | * | 0.0174 | 0.0056 | 3.09 | .002 | .010 | * |  |
| SMN (H)-VAN | 0.0044 | 0.0061 | 0.73 | .467 | .667 |  | 0.0068 | 0.0061 | 1.11 | .266 | .452 |  | 0.0115 | 0.0062 | 1.86 | .063 | .135 |  |  |
| SMN (H)-VN | 0.0068 | 0.0058 | 1.17 | .243 | .431 |  | 0.0195 | 0.0059 | 3.31 | .001 | .008 | * | 0.0049 | 0.0059 | 0.83 | .405 | .525 |  |  |
| SMN (M)-VAN | -0.0065 | 0.0063 | -1.03 | .304 | .495 |  | 0.0062 | 0.0064 | 0.97 | .332 | .496 |  | -0.0007 | 0.0064 | -0.11 | .912 | .948 |  |  |
| SMN (M)-VN | 0.0087 | 0.0061 | 1.42 | .157 | .330 |  | 0.0071 | 0.0062 | 1.14 | .252 | .449 |  | 0.0122 | 0.0062 | 1.98 | .048 | .120 |  |  |
| VAN-VN | 0.0061 | 0.0059 | 1.05 | .296 | .491 |  | 0.0144 | 0.0059 | 2.42 | .016 | .061 |  | 0.0066 | 0.0060 | 1.11 | .266 | .385 |  |  |

Note. Statistics provided for age by baseline general cognitive ability interactions in models of resting-state functional connectivity development by network pair, after adjustment for neighborhood disadvantage at baseline. All network pair associations between general cognitive ability and brain development observed in primary analyses were significant after adjusting for neighborhood disadvantage instead of income-to-needs ratio as the socioeconomic indicator, except CON-DAN, CON-SMN (M), DMN-DAN and DMN-FPN for matrix reasoning, AN-FPN for fluid cognition, AN-VAN for crystallized cognition. Neighbourhood disadvantage indexed by reverse-coded Area Deprivation Index. * Significant at false-discovery rate corrected p-value (pFDR) < .050 over 66 network pairs for each cognitive measure. AN = Auditory network. CON = Cingulo-opercular network. CPN = Cingulo-parietal network. DMN = Default mode network. DAN = Dorsal attention network. FPN = Frontoparietal network. RTN = Retrosplenial network. SN = Salience network. SMN (H) = Somatomotor hand network. SMN (M) = Somatomotor mouth network. VAN = Ventral attention network. VN = Visual network.


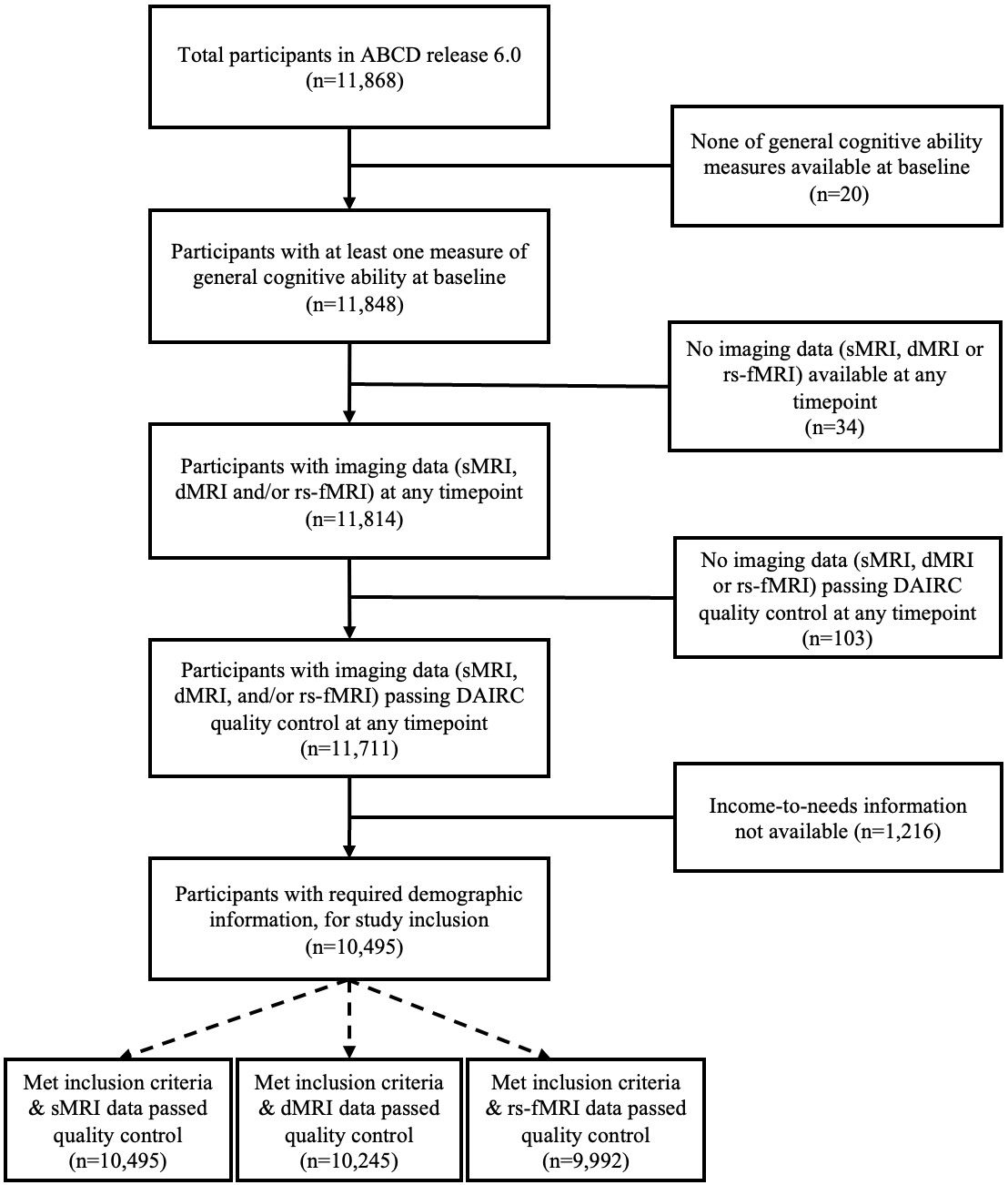


Figure S1. Participant flow diagram. Measures of general cognitive ability included matrix reasoning, fluid cognition, and crystallized cognition. Imaging modalities of interest included structural MRI (sMRI), diffusion MRI (dMRI), and resting-state functional MRI (rsfMRI). The recommended inclusion flag from the Adolescent Brain Cognitive Development Study (ABCD) Data Analysis, Informatics & Resource Center (DAIRC) was used to determine imaging inclusion status (Hagler et al., 2019). Demographic information included age, sex, income-to-needs ratio, study site and family ID. Note: calculation of income-to-needs ratio required valid reporting of household income and number of people in the household to be available. A maximum sample of 10,495 participants met inclusion criteria, of whom 10,495 had sMRI, 10,245 dMRI, and 9,992 rsfMRI data available and passing DAIRC quality control.

**A**

**B**

**
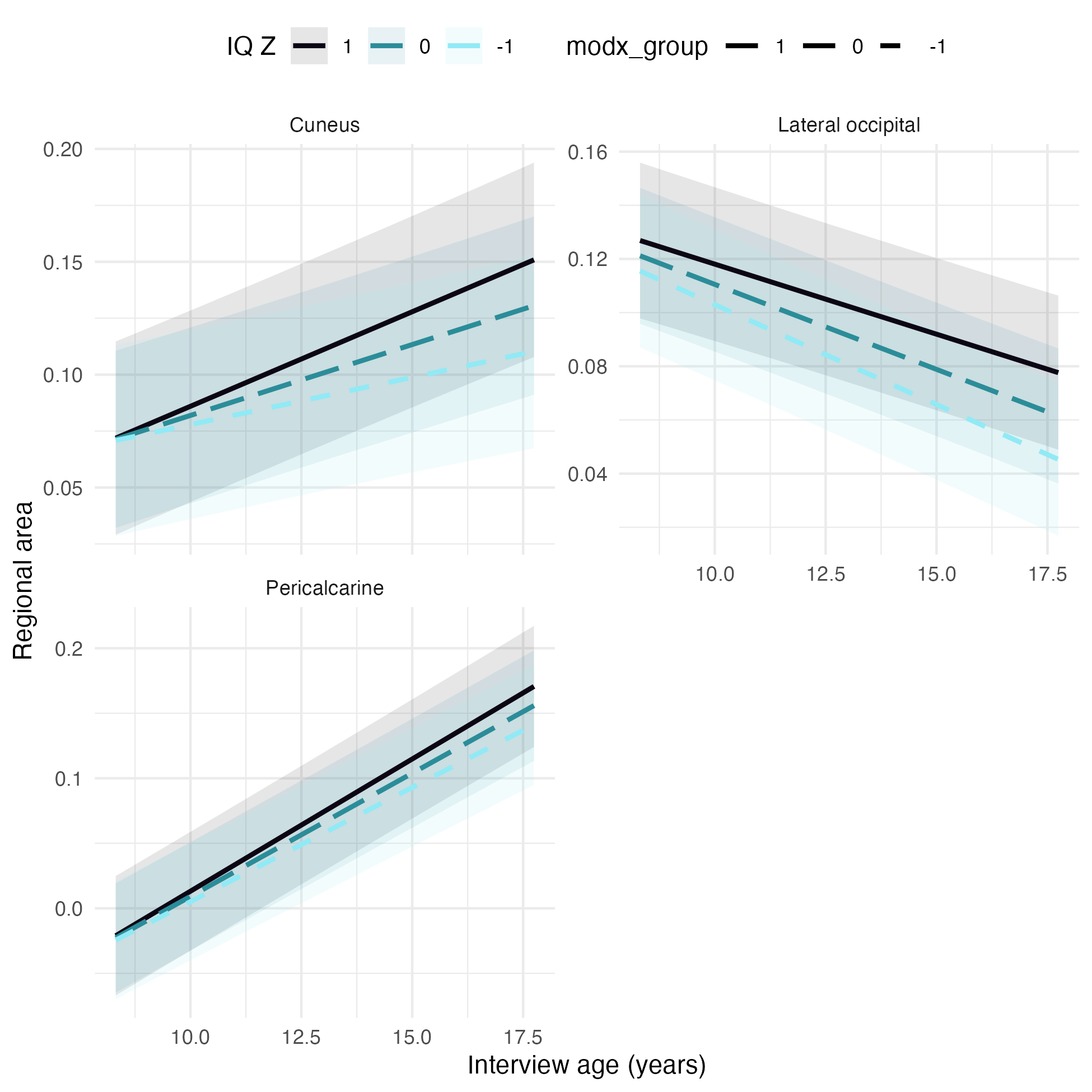

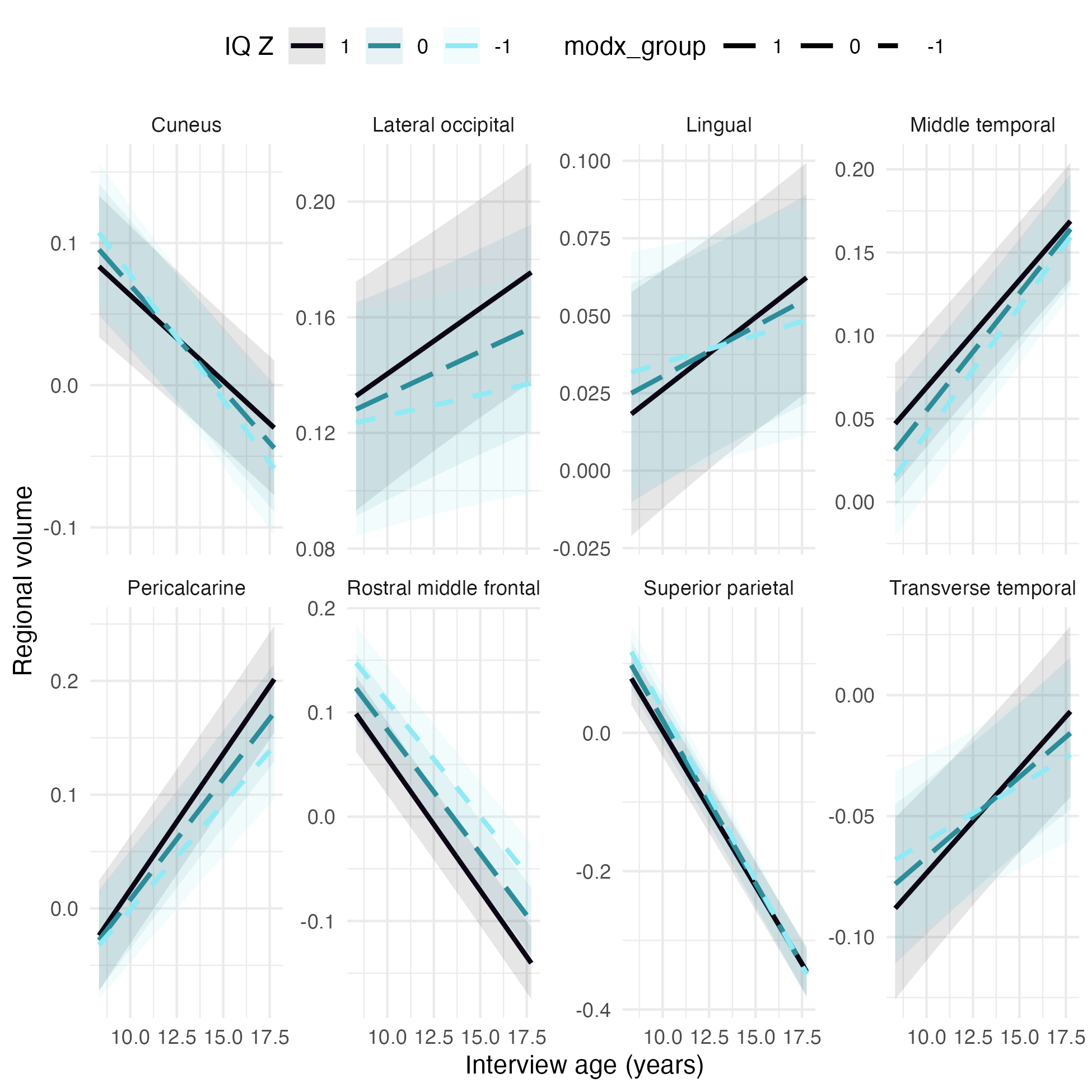
**

**Fluid cognition** Mean - 1 SD Mean Mean + 1 SD

Figure S2. Interaction between age and baseline fluid cognition in models of surface area and volume development by region, controlling for whole-brain total estimates. Association between childhood general cognitive ability indexed by fluid cognition (NIH Toolbox) and the development of regional **A)** surface area after correction for total surface area, and **B)** volume after correction for total volume. Relationships are displayed for regions in which longitudinal surface area trajectories, relative to the whole-brain trajectory, significantly differed by fluid cognition. Best fit lines were estimated and displayed for different baseline fluid cognition values (light teal=mean − 1 SD, dark teal=mean, black=mean + 1 SD).

**A – Matrix reasoning**

**B – Fluid cognition**

**C – Crystallized cognition**

**
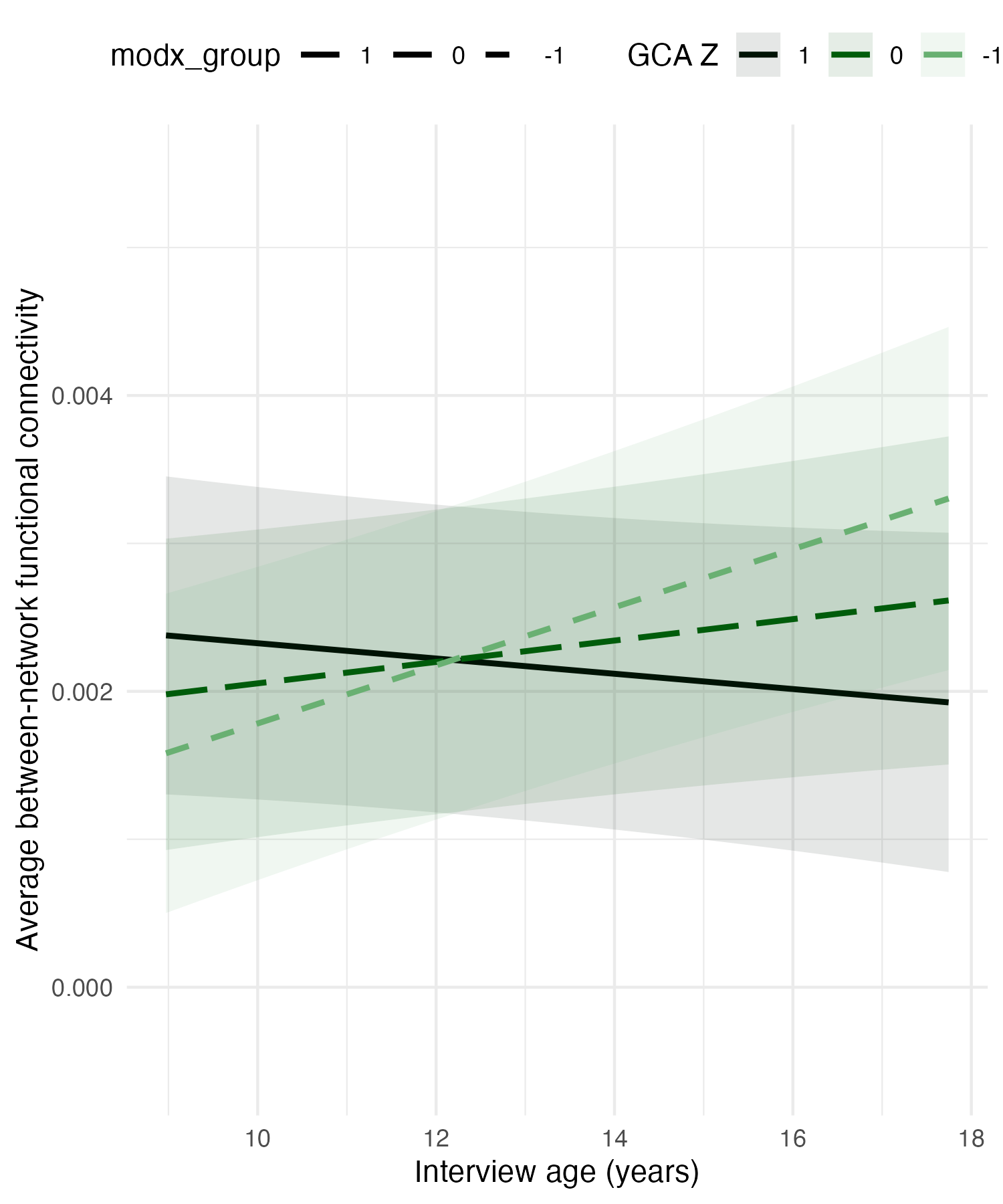

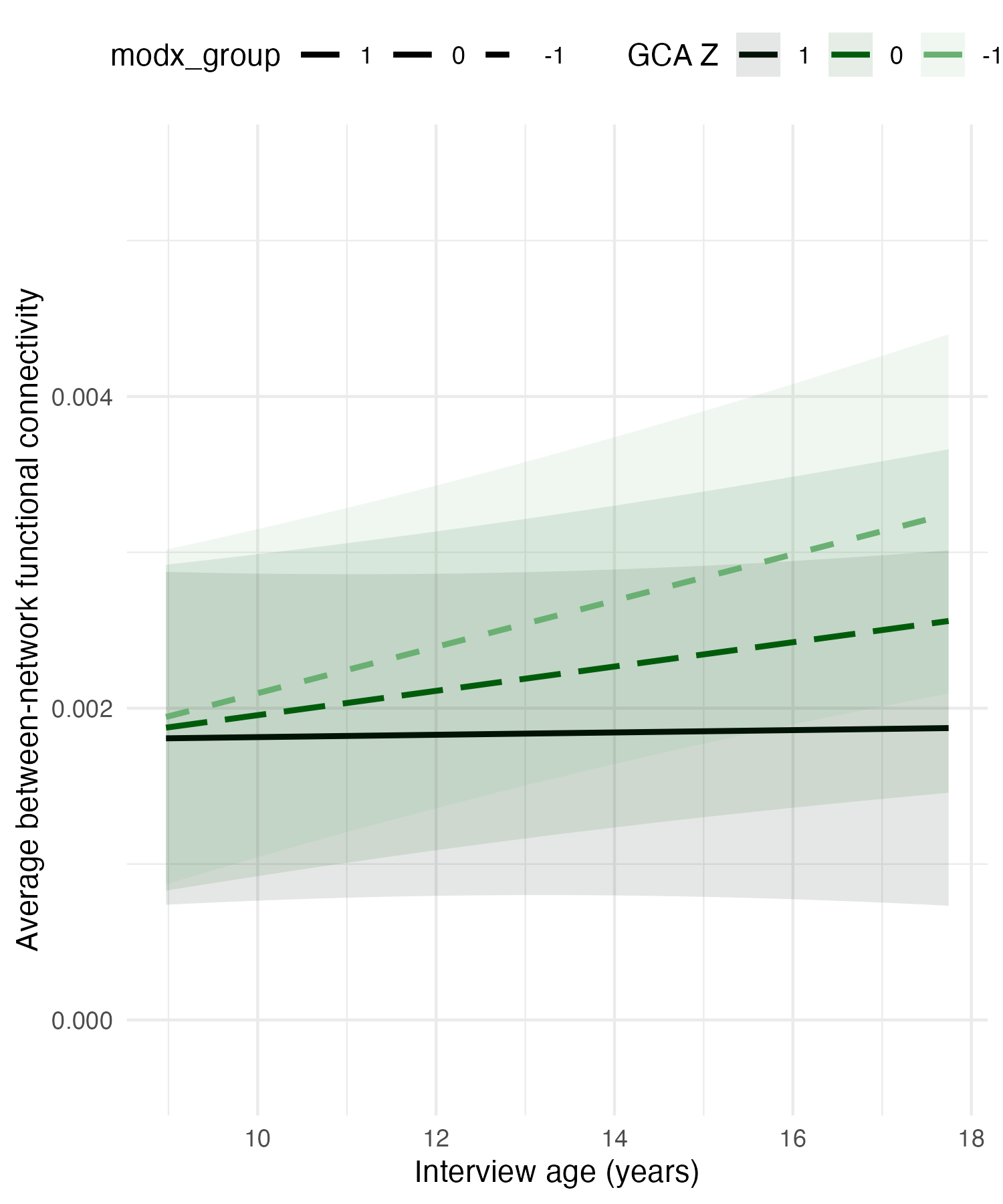

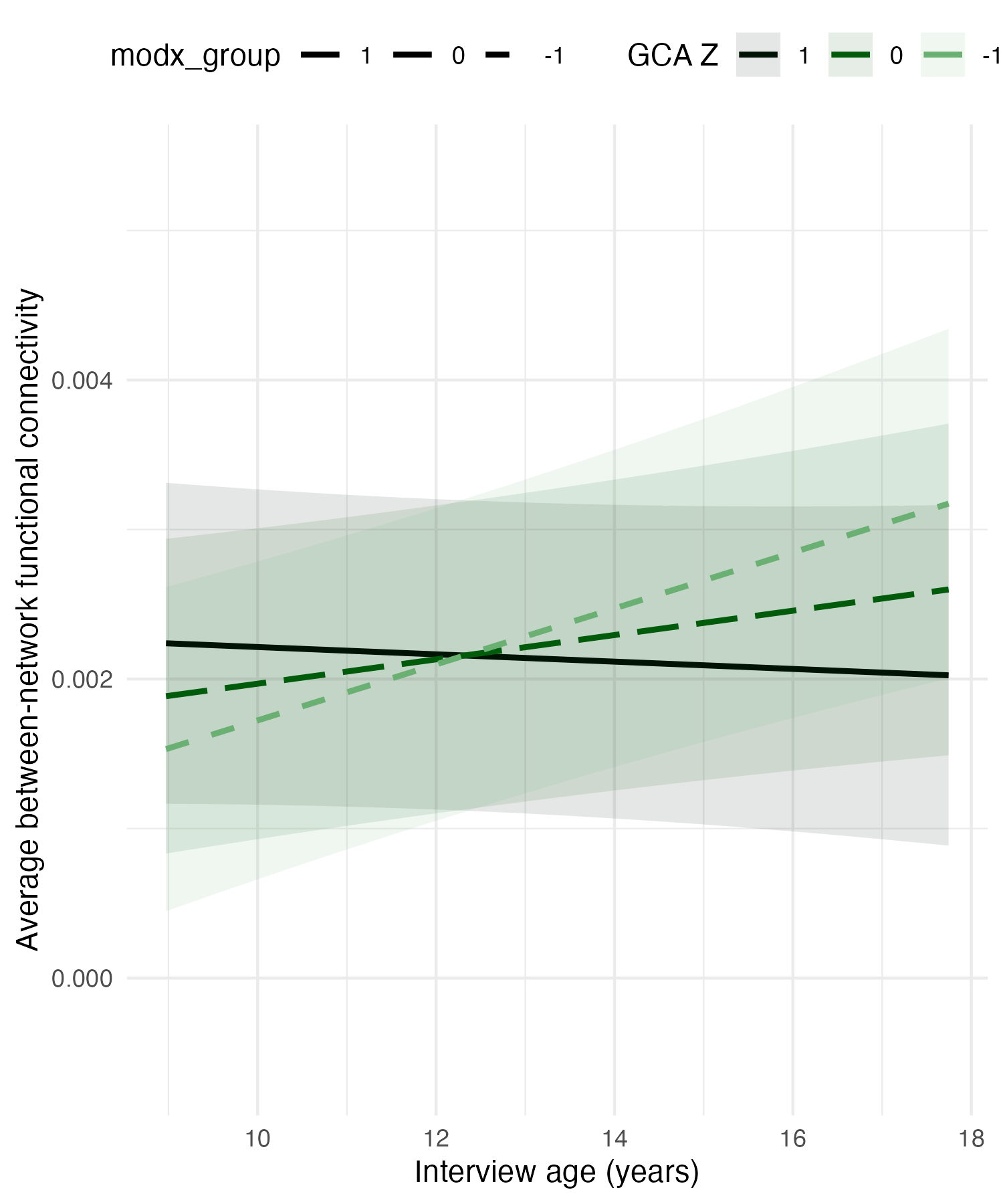
**

**Legend** Mean - 1 SD Mean Mean + 1 SD

Figure S3. Associations between cognition and global between-network rsFC development (unstandardized). Association between the development of between-network resting-state functional connectivity (rsFC) at the whole-brain level and childhood general cognitive ability indexed by: **A)** matrix reasoning (Wechsler Intelligence Scale for Children), **B)** fluid cognition (NIH Toolbox) and **C)** crystallized cognition (NIH Toolbox). Best fit lines for between-network rsFC development were estimated and displayed for different baseline cognition values (light green=mean − 1 SD, dark green=mean, black=mean + 1 SD).


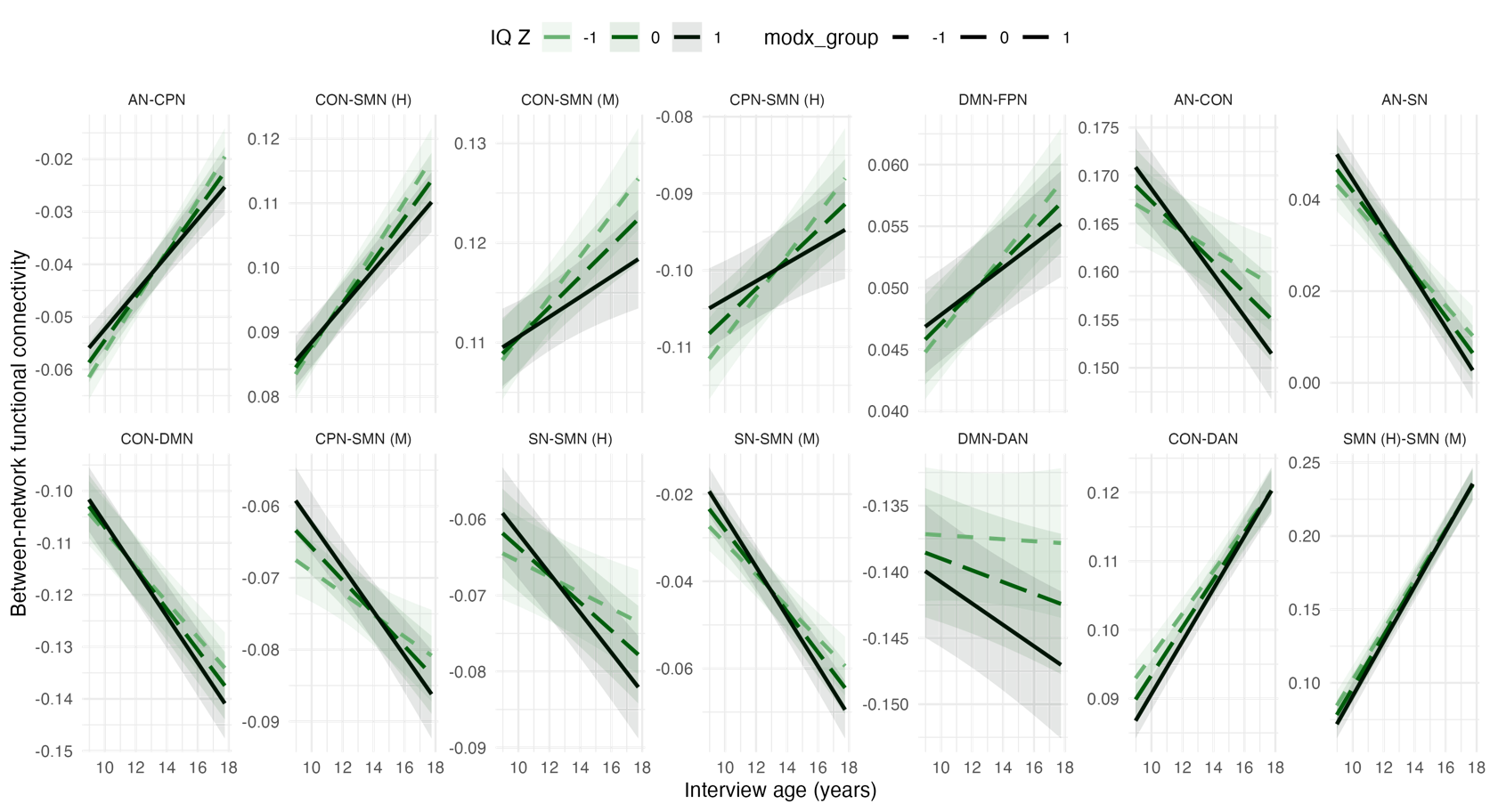


**Matrix reasoning** Mean - 1 SD Mean Mean + 1 SD

Figure S4. Associations between matrix reasoning and pairwise between-network rsFC development (unstandardized). Association between the development of resting-state functional connectivity (rsFC) between pairs of networks and childhood general cognitive ability indexed by matrix reasoning (Wechsler Intelligence Scale for Children). Relationships are displayed for networks pairs between which longitudinal between-network rsFC trajectories significantly differed by cognition. Best fit lines for shown for different baseline matrix reasoning values (light green=mean − 1 SD, dark green=mean, black=mean + 1 SD). AN = Auditory network. CON = Cingulo-opercular network. CPN = Cingulo-parietal network. DMN = Default mode network. DAN = Dorsal attention network. FPN = Frontoparietal network. SN = Salience network. SMN (H) = Somatomotor hand network. SMN (M) = Somatomotor mouth network.

**Crystallized cognition** Mean - 1 SD Mean Mean + 1 SD


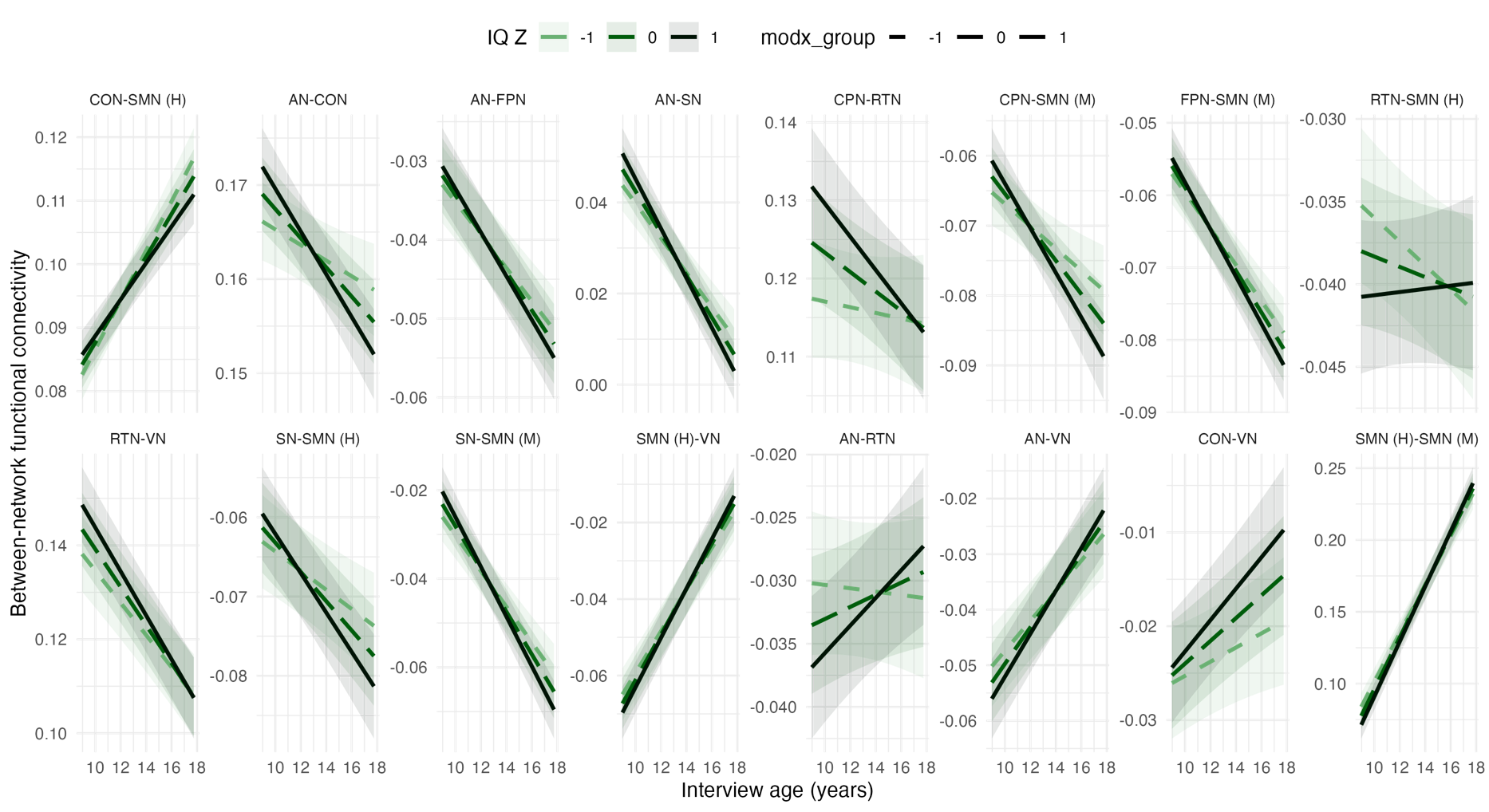


**Fluid cognition** Mean - 1 SD Mean Mean + 1 SD

Figure S5. Associations between fluid cognition and pairwise between-network rsFC development (unstandardized). Association between the development of resting-state functional connectivity (rsFC) between pairs of networks and childhood general cognitive ability indexed by indexed by fluid cognition (NIH Toolbox). Relationships are displayed for network pairs between which longitudinal between-network rsFC trajectories significantly differed by cognition. Best fit lines shown for different baseline fluid cognition values (light green=mean − 1 SD, dark green=mean, black=mean + 1 SD). AN = Auditory network. CON = Cingulo-opercular network. CPN = Cingulo-parietal network. RTN = Retrosplenial network. SN = Salience network. SMN (H) = Somatomotor hand network. SMN (M) = Somatomotor mouth network.VN = Visual network.


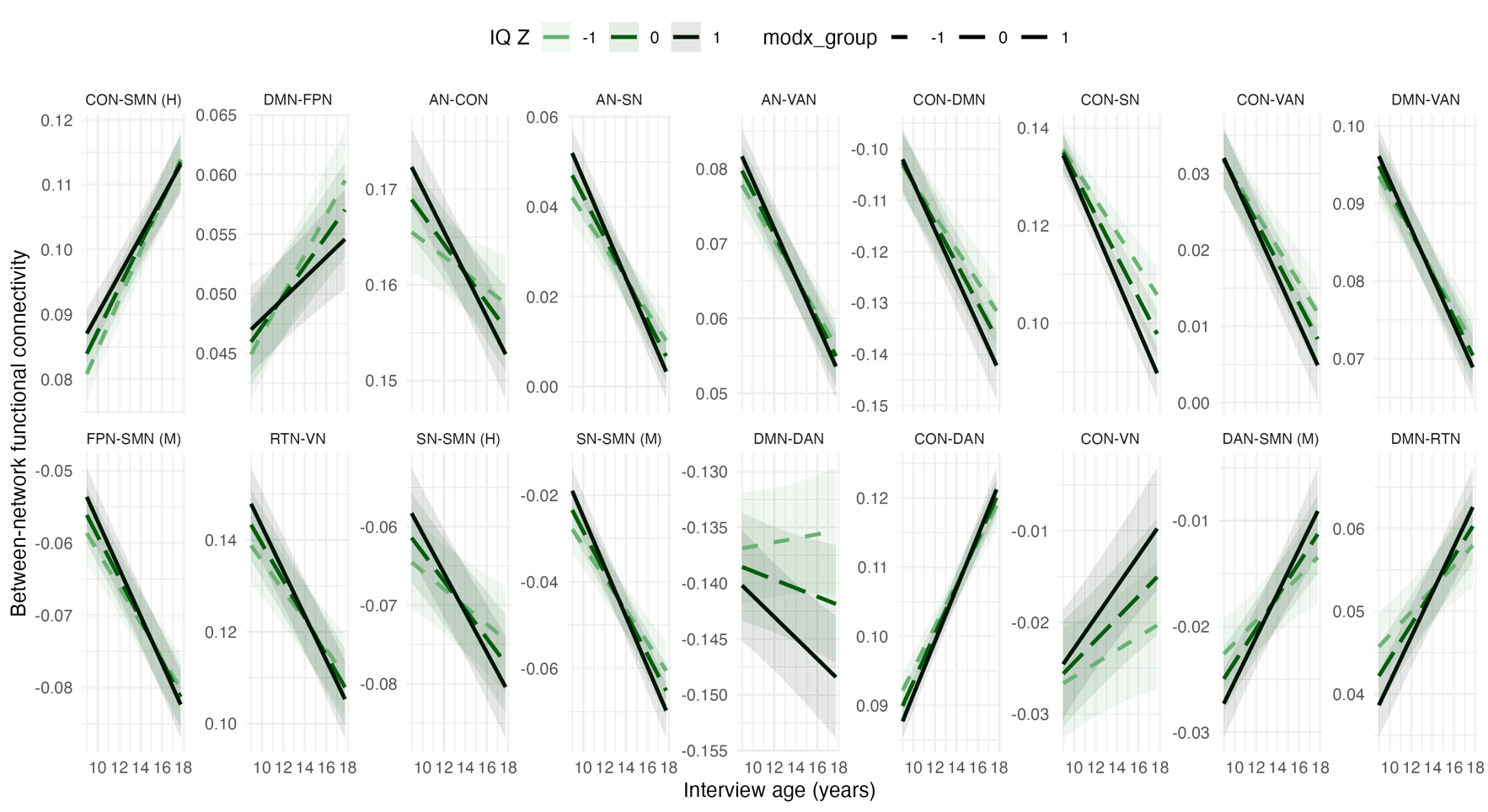


**Crystallized cognition** Mean - 1 SD Mean Mean + 1 SD

Figure S6. Associations between crystallized cognition and pairwise between-network rsFC development (unstandardized). Association between the development of resting-state functional connectivity (rsFC) between pairs of networks and childhood general cognitive ability indexed by indexed by crystallized cognition (NIH Toolbox). Relationships are displayed for network pairs between which longitudinal between-network rsFC trajectories significantly differed by cognition. Best fit lines shown for different baseline crystallized cognition values (light green=mean − 1 SD, dark green=mean, black=mean + 1 SD). AN = Auditory network. CON = Cingulo-opercular network. DMN = Default mode network. DAN = Dorsal attention network. FPN = Frontoparietal network. RTN = Retrosplenial network. SN = Salience network. SMN (H) = Somatomotor hand network. SMN (M) = Somatomotor mouth network. VAN = Ventral attention network. VN = Visual network.
